# Efficacy of a nurse-led patient education intervention in promoting safety skills of patients with inflammatory arthritis treated with biologics: a multicentre randomised clinical trial

**DOI:** 10.1136/rmdopen-2021-001828

**Published:** 2022-03-16

**Authors:** Catherine Beauvais, Françoise Fayet, Alexandra Rousseau, Christelle Sordet, Sophie Pouplin, Yves Maugars, Rose Marie Poilverd, Carine Savel, Véronique Ségard, Béatrice Godon, Christian L’amour, Aleth Perdriger, Fabienne Brin, Patricia Peyrard, Fabienne Chalier, Béatrice Pallot-Prades, Sophie Tuffet, Isabelle Griffoul, Laure Gossec

**Affiliations:** 1 Rheumatology Department, Centre Hospitalier Universitaire Saint Antoine, Sorbonne Université, APHP, Paris, France; 2 Rheumatology, Centre Hospitalier Universitaire de Clermont-Ferrand, Clermont-Ferrand, France; 3 Service de Pharmacologie Clinique et Plateforme de Recherche Clinique de l’Est Parisien, Centre Hospitalier Universitaire Saint Antoine, Sorbonne Université, APHP, Paris, France; 4 Rheumatology Department, Hôpitaux Universitaires de Strasbourg, Strasbourg, France; 5 Service de Rhumatologie, Centre Hospitalier Universitaire de Rouen, Rouen, France; 6 Rheumatology, Centre Hospitalier Universitaire de Nantes Hôpital Saint Jacques, Nantes, France; 7 Medical Faculty, Universite de Nantes Pole Sante, Nantes, France; 8 Rheumatology Department, Centre Hospitalier Universitaire Saint Antoine, Sorbonne Université, APHP, Paris, France; 9 Rheumatology Department, Centre Hospitalier Universitaire de Rouen, Rouen, France; 10 Rheumatology Department, Centre Hospitalier Universitaire Pitié Salpétrière, Sorbonne Université, APHP, Paris, France; 11 Rhumatologie, Centre Hospitalier Universitaire de Rennes, Rennes, France; 12 Rheumatology Department, Centre Hospitalier Universitaire de Nantes, Nantes, France; 13 Rheumatology Department, Centre Hospitalier Universitaire de Saint-Etienne, Saint-Etienne, France; 14 Rheumatology Department, Centre Hospitalier Régional Universitaire de Tours, Tours, France; 15 Rheumatology, Centre Hospitalier Regional Universitaire de Tours, Tours, France; 16 INSERM, Institut Pierre Louis d'Epidémiologie et de Santé Publique, INSERM, Sorbonne Universite, Paris, France; 17 APHP, Rheumatology Department, Hopital Universitaire Pitie Salpetriere, Paris, France

**Keywords:** arthritis, biological therapy, patient care team, nursing

## Abstract

**Objective:**

To evaluate the effect of a nurse-led patient education on safety skills of patients with inflammatory arthritis treated with biologic disease-modifying antirheumatic drugs (bDMARDs).

**Methods:**

This is a multicentre, open-labelled, randomised controlled trial comparing an intervention group (face-to-face education by a nurse at baseline and 3 months later) with a control group (usual care) at the introduction of a first subcutaneous bDMARD. The primary outcome was score on the BioSecure questionnaire at 6 months (0–100 scale), a validated questionnaire assessing competencies in dealing with fever, infections, vaccination and daily situations. The secondary outcomes were disease activity, coping, psychological well-being, beliefs about medication, self-efficacy and severe infection rate.

**Results:**

129 patients with rheumatoid arthritis and spondyloarthritis were enrolled in nine rheumatology departments; 122 completed the study; 127 were analysed; and 64 received the intervention (mean duration: 65 min at baseline and 44 min at 3 months). The primary outcome was met: the BioSecure score was 81.2±13.1 and 75.6±13.0 in the education and usual care groups (difference: +6.2, 95% CI 1.3 to 11.1, p=0.015), demonstrating higher safety skills in the education group. Exploratory analyses showed better skills regarding infections, greater willingness for vaccinations and greater adherence-related behaviours in the education group. Coping was significantly more improved by education; other secondary outcomes were improved in both groups, with no difference.

**Conclusions:**

Educating patients was effective in promoting patient behaviours for preventing adverse events with bDMARDs. An education session delivered to patients starting a first bDMARD can be useful to help them self-manage safety issues.

**Trial registration number:**

NCT02855320.

**Key messages**

**What is already known about this subject?**

- Biologic disease-modifying antirheumatic drugs (bDMARDs) are effective treatments for inflammatory arthritis but can lead to safety issues, which could be prevented by educating patients.
- Recommendations regarding safety exist for health professionals, such as vaccinations or dealing with situations at risk, but educating patients on safety matters has not been evaluated in controlled trials.

**What does this study add?**

- This multicentre trial showed that a face-to-face nurse-led patient education at baseline and at 3 months was effective in terms of patients’ safety competencies at 6 months, such as dealing with infections or willingness for vaccination.
- Feasibility was good with a 40–65 min duration of intervention at two time points.

**How might this impact on clinical practice or further developments?**

- A patient education session delivered to patients starting a first bDMARD can be useful to help manage safety.

## Introduction

Biologic disease-modifying antirheumatic drugs (bDMARDs) are effective treatments for inflammatory arthritis (IA), including rheumatoid arthritis (RA), spondyloarthritis (SpA) and psoriatic arthritis (PsA), to control disease activity, reduce functional disability and improve prognosis.^1–4^

However, patients receiving bDMARDs are at risk of adverse events, such as excess risk of infection, noted more in RA^5–7^ than in SpA,^8 9^ in particular due to comedication with glucocorticoids and/or high disease activity.^7 8 10^ The most common infections include bronchitis and pneumonia, pyelonephritis, bone/joint infections, and skin or soft tissue infections. Severe infections, that is, infections leading to hospitalisation or intravenous treatment, are mainly bacterial.^7 11 12^

Measures patients can take to decrease risks of adverse events include influenza and pneumococcal vaccinations, as recommended by EULAR.^13^ However, patients with IA have a suboptimal uptake of vaccinations, in part due to a low rate of referrals for vaccination by rheumatologists and patients’ fear of vaccination.^14 15^ Self-management of infectious situations also includes self-referral and bDMARD interruption.^16–18^ Furthermore, patients need to discuss other events such as surgery, dental care or pregnancy with their healthcare professionals (HCPs) and be aware that some drugs should be interrupted in these situations.^16–19^

Given all these everyday situations that require patients to be careful with their medications, safety training should be a significant part of patient education. Patient education is recommended in chronic disorders,^20^ specifically in IA,^21^ to help patients acquire specific skills to better manage their disease. Patients’ abilities to make decisions to preserve their own life and health, termed ‘life-saving self-care skills’,^20 22^ include cognitive, practical and behavioural knowledge, data interpretation, and problem-solving.^23^ Like other non-pharmacological interventions, patient education should be evaluated.^20 21^ Safety skills can be assessed by using the validated BioSecure questionnaire, which includes multiple-choice questions and scenarios of potential safety threats and assesses patients’ problem-solving abilities.^24^

Rheumatology nurses play a major role in patient education.^25^ With regard to infection prevention, controlled trials have shown the beneficial impact of rheumatology nurses in screening for comorbidities and increasing the prescription of vaccines by the rheumatologist or general practitioner (GP).^26 27^ Therefore, rheumatology nurses should be involved in safety matters.

In France, bDMARDs should only be prescribed in a hospital setting, which is done during face-to-face consultations between the patient and the rheumatologist. In some rheumatology departments, patients may have an interview with a nurse on safety issues after bDMARDs are prescribed. However, consultations with nurses are not available in all departments and most patients get information from the rheumatologist only. In this context, here we report the results of a randomised controlled trial to assess the efficacy of nurse-led education on patients’ safety skills related to bDMARDs.

## Methods

This was a multicentre, controlled, open-labelled, parallel-group, randomised trial with blinded assessment which followed the Consolidated Standards of Reporting Trials reporting guidelines. The original and final protocols are provided in online supplemental material 1. Substantial changes made to the methods after trial commencement are detailed in online supplemental eAppendix 1 in online supplemental material 2.

10.1136/rmdopen-2021-001828.supp1 Supplementary data

All patients gave their written informed consent before participation.

### Participants

#### Patients

Patients visiting nine secondary and/or tertiary care rheumatology departments in France were invited to participate and were enrolled by the rheumatologist from January 2017 to April 2018, with a final follow-up on 26 November 2018.

#### Inclusion criteria

The inclusion criteria were age 18–75 years; diagnosis of RA (fulfilling the 2010 American College of Rheumatology/EULAR classification criteria^28^) or diagnosis of axial or peripheral SpA, including PsA (fulfilling the 2009 Assessment of SpondyloArthritis international Society classification criteria^29 30^); bDMARD-naïve; eligible for a first subcutaneous bDMARD for active disease according to the rheumatologist’s opinion, referring to national recommendations (inadequate response to conventional medications or disease-modifying antirheumatic drugs and no contraindications to bDMARDs^2 4^); and able to complete self-administered questionnaires.

#### Exclusion criteria

The exclusion criteria were conditions that could alter patients’ understanding or adherence to treatment, such as cognitive impairment and previous education targeted to bDMARDs by a nurse. Prior generic patient education related to the disease was not an exclusion criterion.

#### Nurses

Participating nurses were recruited on a voluntary basis to perform patient education. The nurses had specific training in patient education according to the French regulations on educational programmes and were part of multidisciplinary hospital-based patient education teams.

### Randomisation

Patients were assigned in a 1:1 ratio to the intervention or control group. Centralised computer randomisation was performed at the end of the inclusion visit (CleanWEB Telemedicine Technologies SAS, Boulogne-Billancourt, France). A block balanced randomisation list was established by using permuted blocks of variable width not communicated to the investigators.

### Intervention and control groups

The intervention protocol, content and tools were established during a face-to-face meeting in December 2015 with the participating nurses, the two principal investigators (CB, LG) and one patient from a patient association.

The intervention consisted of two face-to-face education sessions, at baseline and 3 months later, focused on safety skills and self-injections according to the French Society of Rheumatology guidelines^2 4^ (online supplemental eAppendix 2 in online supplemental material 2). Each session was preceded by a nurse’s individualised assessment of patients’ expectations, concerns and motivation for the prescribed bDMARD (online supplemental eAppendix 3 and 4 in online supplemental material 2). The baseline session was supported by a booklet outlining relevant messages (online supplemental eAppendix 5 in online supplemental material 2). The intervention duration and completion were reported by the nurse.

Both groups received usual care, which consisted of information about bDMARDs given by the rheumatologist and usual care follow-up on an outpatient basis in hospital or in private care by the treating rheumatologist, with reference to management recommendations.^2 4^ The rheumatologist was informed of their patients who were participating in the study but was blinded to the randomisation group.

### Outcome measures and collected data

The primary outcome was the BioSecure questionnaire score assessed at 6 months. This validated questionnaire has good reproducibility^24^ and sensitivity to change^31^ and evaluates patients’ skills in managing risk situations: fever, infections, vaccinations, surgery, dental care and pregnancy. Additional questions relate to bDMARD adherence behaviours, in particular in case of remission. Adherence was considered a safety behaviour because discontinuation can lead to flares and high disease activity is associated with an increased risk of infections.^10^ The questionnaire contains multiple-choice questions and ‘situation scenarios’ of hypothetical life events. The BioSecure questionnaire consists of 55 questions. Each correct answer is associated with 1 point. A missing answer is considered a wrong answer. The global BioSecure score is calculated as the sum of the points obtained. It is then multiplied by 1.82 to relate to base 100, with higher scores indicating higher safety skills. The BioSecure questionnaire was not administered at baseline because completing this questionnaire could be considered in itself part of an educational process: this questionnaire is not a patient-related outcome, but an assessment tool containing educational questions and it was administered prior to randomisation. Therefore, there was a risk that the content of the questionnaire would be discussed during the patient interface not only with the nurse but also with the rheumatologist.

The comparability of the groups regarding patient knowledge was checked by collecting patients’ opinions about their level of information and their information sources.

At baseline, the collected data were as follows: sociodemographics, disease and treatment characteristics, type of follow-up, comorbidities, and number of severe infections in the 2 years before recruitment, defined as infections requiring hospitalisation or intravenous antibiotics.

Prespecified secondary outcomes were collected at baseline and at 6 months: disease activity,^32–34^ coping and psychological well-being measured by numeric rating scales (NRS) derived from the Rheumatoid Arthritis Impact of Disease (RAID) score,^35^ the Arthritis Helplessness Index (AHI)^36^ and the Beliefs About Medicines Questionnaire (BMQ).^37^ At 6 months, the number of severe infections during the 6 months of the study was collected.

Exploratory outcomes were the safety skills on the BioSecure questionnaire, which were gathered by key subscores related to six domains: infections, dental care and surgery, vaccinations, child conception, adherence-related behaviours and drug storage/cold chain preservation. These were analysed post-hoc to explore the skills most gained through patient education.

### Statistical analysis

The statistical analysis plan is available in online supplemental file 3. Sample size calculation was based on the national survey conducted in France,^38^ where the mean BioSecure score was 68.09±18.28 with usual care and 75.66±14.20 for people who had received some kind of education (7.57-point difference). In this trial enrolling patients who were bDMARD-naïve, 129 randomised patients were needed to achieve 80% power to detect a relative 10-point difference of the score in the intervention group, considering a two-sided alpha of 5% and 25% dropout rate.

10.1136/rmdopen-2021-001828.supp3 Supplementary data

Baseline characteristics were reported with numbers (%) for categorical variables and mean (SD) or median (IQR) for quantitative variables, depending on their distribution. The BioSecure score at 6 months was compared by Student’s t-test in the modified intention-to-treat population, including all randomised patients with confirmed eligibility for a first subcutaneous bDMARD. Missing responses on the questionnaire were considered a wrong answer. If the questionnaire was not completed, single imputation involved the 25th percentile value of the population with a completed questionnaire, a failure hypothesis. Sensitivity analysis was performed on the per-protocol population, excluding patients with a missing primary outcome value.

Changes in secondary outcomes were compared by a linear regression model adjusted on the baseline value of the outcome with normally distributed values or Wilcoxon rank-sum test with non-normally distributed values. For patients with available data, the BioSecure score was compared by sex and socioprofessional status with the Student’s t-test and by disease with analysis of variance. Correlation between the BioSecure score and age or disease duration was assessed by Spearman correlation analysis.

Additional analyses were performed to assess the impact of the intervention on the BioSecure score, adjusted on the degree of patient information about their treatments at inclusion (>7/10 or ≤7/10) using a linear regression model.

For post-hoc analysis, the difference in the proportion of good responses in the six key domains of the BioSecure questionnaire was calculated with its continuity-corrected Wald 95% CI. A centre effect was looked for using a linear mixed model considering each participating centre as a random effect.

All analyses were performed with SAS V.9.4. All tests were two-sided and p<0.05 indicated statistical significance. There was no correction for multiplicity analyses.

### Minimising bias and preserving parallel groups

Several measures were used to reduce bias related to the open-label design: the randomisation took place after the baseline assessments were completed; patients assigned to the control group did not meet the education nurses at any time during the study; patients were informed at baseline that they would benefit from an educational face-to-face interview with the nurse at 6 months; and the 6-month assessment was by an HCP blinded to the randomisation.

## Results

### Patients

**Figure 1:**
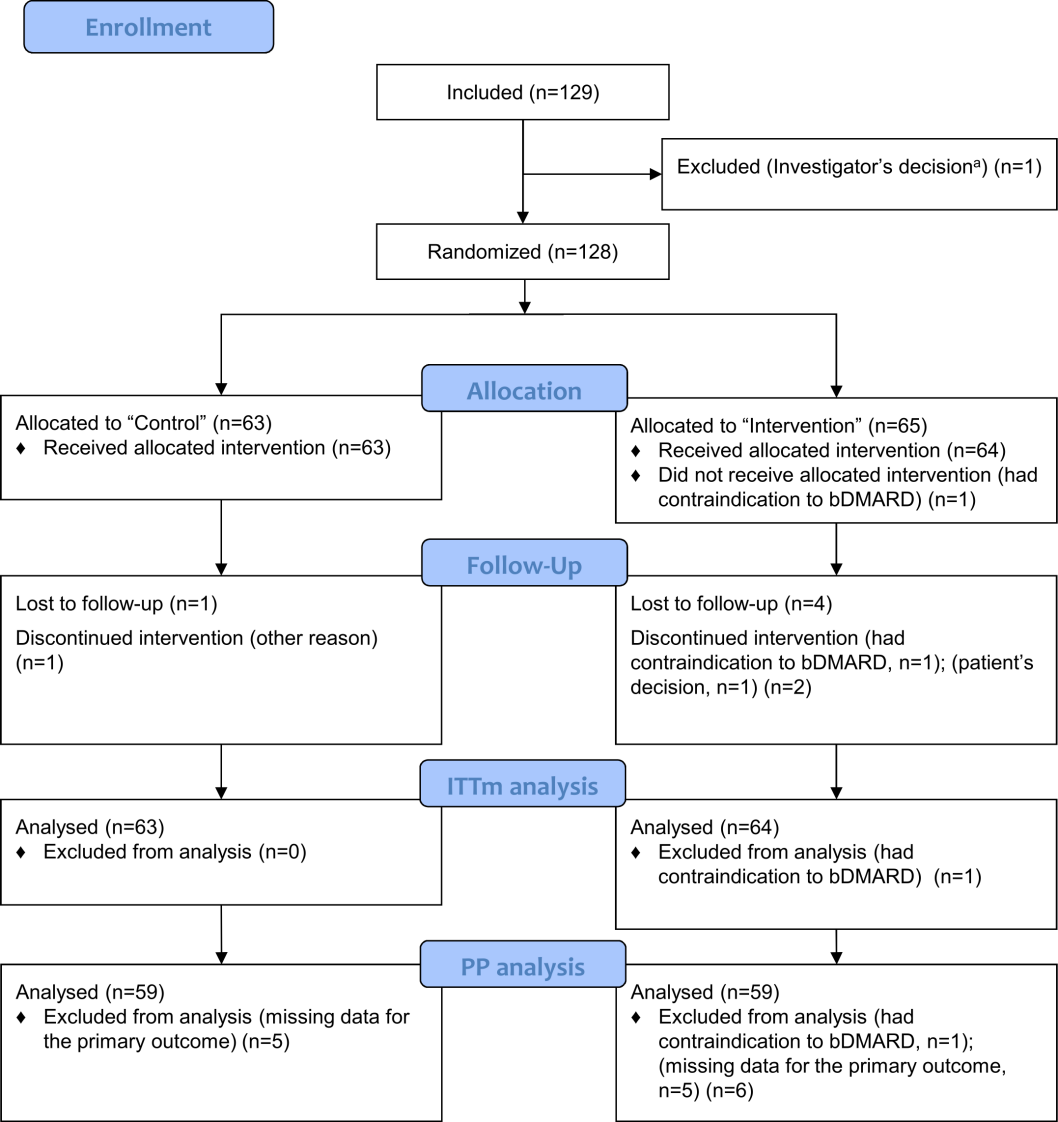
CONSORT 2010 flow diagram. ^a^Error regarding inclusion criteria. bDMARD, biologic disease-modifying antirheumatic drug; CONSORT, Consolidated Standards of Reporting Trials; ITT, intention-to-treat; PP, per protocol.

Of the 129 enrolled patients, only 128 were randomised (figure 1) due to an error regarding inclusion criteria and 1 patient had a contraindication to a bDMARD, which led to data for 127 patients analysed: 64 in the intervention group and 63 in the control group. Of these, 30.7% had RA and 69.3% had SpA. One patient was lost to follow-up in the control group and 4 patients in the intervention group; 122 of 127 patients (96%) completed the study.

The groups were similar in baseline characteristics and comorbidities (tables 1 and 2, online supplemental eAppendix 1 in online supplemental material 4), except for a higher number of patients with disability in the intervention than in the control group (25.8% vs 10.0%) and a higher frequency of patients with prior generic patient education in the intervention than in the control group (14.3% vs 9.8%).

**Table 1:** Baseline characteristics: demographics, patient information and education, and type of follow-up

	Control group (n=63)		Intervention group (n=64)	
	n*		n*	
Female, n (%)	63	44 (69.8)	64	40 (62.5)
Age, mean (SD), years	63	45.4 (13.0)	64	48.6 (12.6)
Professional activity	60		62	
Currently employed		46 (76.7)		35 (56.5)
Retired		8 (13.3)		11 (17.7)
On sick leave/disability		6 (10.0)		16 (25.8)
Socioprofessional status (SPS), n (%)	61		64	
Higher SPS		21 (34.4)		25 (39.1)
Lower SPS		36 (59.0)		36 (56.3)
Other		4 (6.6)		3 (4.7)
Size of place of residence (inhabitants), n (%)	62		63	
≥200 000		9 (14.5)		14 (22.2)
10 000–199 999		16 (25.8)		18 (28.6)
<10 000		37 (59.7)		31 (49.2)
Education level, n (%)	62		64	
High school or less		27 (43.5)		32 (50.0)
University		35 (56.5)		32 (50.0)
Family status, n (%)	62		64	
Living alone or single		12 (19.4)		10 (15.6)
Living with family/in a couple or family relationship		50 (80.6)		54 (84.4)
Current tobacco consumption, n (%)	62	13 (21.0)	64	25 (39.1)
Usual care follow-up, n (%)	63		64	
Only by rheumatologist in hospital		23 (36.5)		25 (39.1)
Only by rheumatologist in private practice		17 (27.0)		21 (32.8)
Both in hospital and private practice		23 (36.5)		18 (28.1)
Self-reported patients’ information about the disease† (0–10), median (IQR)	62	8.0 (6.0–9.0)	63	8.0 (6.0–9.0)
Self-reported patients’ information about treatment (including bDMARDs)† (0–10), median (IQR)	62	7.0 (5.0–8.0)	63	7.0 (6.0–9.0)
Patients’ information sources about disease or treatments, n (%)				
General practitioner	61	41 (67.2)	61	37 (60.7)
Rheumatologist in private care	61	36 (59.0)	60	39 (65.0)
Rheumatologist in hospital	62	55 (88.7)	62	56 (90.3)
Face-to-face generic patient education	61	6 (9.8)	56	8 (14.3)
Group patient education	60	1 (1.7)	56	4 (7.1)
Nurse	62	19 (30.6)	59	22 (37.3)
Pharmacist	61	9 (14.8)	58	13 (22.4)
Physiotherapist	61	16 (26.2)	60	11 (18.3)
Other health practitioner	60	1 (1.7)	57	5 (8.8)
Relatives	60	16 (26.7)	56	17 (30.4)
Patient association	60	5 (8.3)	56	6 (10.7)
Internet	62	41 (66.1)	60	44 (73.3)
Brochures or leaflets	62	39 (62.9)	58	32 (55.2)
Books	62	5 (8.1)	55	4 (7.3)
Television	61	7 (11.5)	57	12 (21.1)
Other	56	3 (5.4)	51	2 (3.9)
Information given by the patient’s doctor about bDMARDs (yes), n (%)	62	37 (59.7)	62	39 (62.9)

*Number of available data.

†High score indicates better score.

bDMARDs, biological disease-modifying antirheumatic drugs.

**Table 2:** Baseline characteristics: disease, treatments and outcome measures

	Control group (n=63)		Intervention group (n=64)	
	n*		n*	
Type of inflammatory arthritis, n (%)	63		64	
Rheumatoid arthritis		17 (27.0)		22 (34.4)
Axial spondyloarthritis		39 (61.9)		33 (51.6)
Peripheric spondyloarthritis		7 (11.1)		9 (14.1)
Disease duration, median (IQR), years	63	4.0 (0.8–10.0)	64	2.4 (0.8–6.8)
Treatments				
NSAIDs, n (%)	63	32 (50.8)	64	29 (45.3)
GCs, n (%)	63	13 (20.6)	64	18 (28.1)
GC current dosage (mg/day), median (IQR)	12	7.3 (4.0–10.0)	18	10.0 (7.5–20.0)
At least one current cDMARD, n (%)	62	23 (37.1)	64	27 (42.2)
Current methotrexate, n (%)	62	17 (27.4)	64	22 (34.4)
Current leflunomide, n (%)	62	2 (3.2)	64	2 (3.1)
Current sulfasalazine, n (%)	62	1 (1.6)	64	2 (3.1)
Other current cDMARD, n (%)	62	3 (4.8)	64	1 (1.6)
Number of cDMARDs (including current cDMARD), n (%)	62		64	
0		39 (62.9)		37 (57.8)
1		16 (25.8)		21 (32.8)
≥2		7 (11.3)		6 (9.4)
DAS28†, mean (SD)	17	3.9 (1.8)	26	4.1 (1.2)
BASDAI (0–10)†, mean (SD)	44	5.5 (1.9)	41	5.6 (2.1)
ASDAS†, mean (SD)	43	3.2 (0.9)	36	3.1 (0.8)
Coping (0–10)†‡, mean (SD)	62	4.3 (2.2)	63	4.7 (2.4)
Psychological well-being (0–10)†‡, mean (SD)	62	4.8 (2.4)	64	4.7 (2.4)
AHI (5–20)†, mean (SD)	58	13.0 (2.6)	56	12.5 (2.6)
BMQ necessity score (5–25)§, median (IQR)	55	20.0 (19.0–23.0)	60	21.0 (18.0–23.5)
BMQ concerns score (5–25)†, mean (SD)	56	15.2 (4.6)	57	15.4 (4.1)
Serious infections before enrolment¶, n (%)	63	2 (3.2)	64	4 (6.3)
bDMARDs prescribed, n (%)	63		64	
Anti-TNFα		61 (96.8)		59 (92.2)
Other		2 (3.2)		5 (7.8)

Control group: 1 pulmonary infection, 1 digestive infection; intervention group: 1 lung infection, 2 urinary tract infections and 1 undocumented.

*Number of available data.

†High score indicates poor score.

‡From the Rheumatoid Arthritis Impact of Disease.

§High score indicates good score.

¶Infections requiring hospitalisation or intravenous antibiotics; patients concerned had only one infection that required hospitalisation.

AHI, Arthritis Helplessness Index; Anti-TNFα, Anti-Tumor Necrosing Factor α; ASAS, Assessment of SpondyloArthritis international Society; ASDAS, ASAS-endorsed Disease Activity Score; BASDAI, Bath Ankylosing Spondylitis Disease Activity Index; bDMARDs, biologic disease-modifying antirheumatic drugs; BMQ, Beliefs About Medication Questionnaire; cDMARDs, conventional disease-modifying antirheumatic drugs; DAS28, Disease Activity Score in 28 joints; GCs, glucocorticoids; NSAIDs, non-steroidal anti-inflammatory drugs.

### Primary outcome

At 6 months, the BioSecure questionnaire was fully completed by 71 (55.9%) patients; 37 (29.1%) questionnaires had one missing response and 10 (7.9%) had more than one missing response. The least filled-in question (missing for 22.6% of patients) was the last question, which was an open-ended question. Finally, nine (7.1%) questionnaires were missing and the score was imputed as 70.98 (observed 25th percentile). The mean (SD) BioSecure score at 6 months was 81.2 (13.1) and 75.6 (13.0) in the intervention and control groups (difference: +5.6, 95% CI 1.1 to 10.2, p=0.016), showing better skills in the education than in the usual care group (table 3). Similar results were obtained in the per-protocol analysis: mean scores 82.1 (13.3) and 75.9 (13.3) in the intervention and control groups (difference: +6.2, 95% CI 1.3 to 11.1, p=0.013).

**Table 3:** Results of the primary outcome at 6 months and differences in both groups in secondary outcomes between baseline and 6 months

	Control group (n=63)		Intervention group (n=64)		Between-group difference (95% CI)	P value
	n*		n*			
Primary outcome						
BioSecure score† at 6 months, mean (SD)	63	75.6 (13.0)	64	81.2 (13.1)	5.6 (1.1 to 10.2)	0.016‡
Secondary outcomes						
DAS28§ difference (6 months–BL), mean (SD)	12	−1.3 (1.7)	20	−1.9 (1.3)	(−0.88 to 0.50)	0.5850¶
BASDAI§ difference (6 months–BL) (0–10), mean (SD)	43	−1.1 (1.8)	38	−1.6 (2.1)	(−1.29 to 0.42)	0.3211¶
ASDAS§ difference (6 months–BL), median (IQR)	39	−0.7 (−1.5 to 0.0)	32	−0.7 (−1.7 to −0.1)		0.8448**
Coping§ difference (6 months–BL) (0–10), mean (SD)	58	−0.7 (2.8)	57	−1.9 (2.3)	(−1.80 to −0.11)	0.0275¶
Psychological well-being§ difference (6 months–BL) (0–10), mean (SD)	58	−1.0 (3.0)	57	−1.4 (2.4)	(−1.40 to 0.36)	0.2453¶
AHI§ score difference (6 months–BL) (5–20), mean (SD)	48	−1.2 (2.6)	45	−1.4 (3.0)	(−1.29 to 0.72)	0.5702¶
BMQ necessity† difference (6 months–BL) (5–25), mean (SD)	46	0.2 (3.8)	49	0.5 (3.4)	(−0.85 to 1.72)	0.5001¶
BMQ concerns§ score difference (6 months–BL) (5–25), mean (SD)	49	−1.0 (3.5)	48	−1.5 (4.8)	(−2.12 to 0.78)	0.3623¶
Severe infections†† within 6 months, n (%)	61	1 (1.6)	58	0 (0)		1.0000‡‡

*Number of available data.

†High score indicates good score.

‡Student’s t-test.

§High score indicates poor score.

¶Linear regression adjusted on the initial score value.

**Wilcoxon test.

††Infections requiring hospitalisation or intravenous antibiotics.

‡‡Fisher’s exact test.

AHI, Arthritis Helplessness Index; ASAS, Assessment of SpondyloArthritis international Society; ASDAS, ASAS-endorsed Disease Activity Score; BASDAI, Bath Ankylosing Spondylitis Disease Activity Index; BL, Baseline; BMQ, Beliefs About Medication Questionnaire; DAS28, Disease Activity Score in 28 joints.

### Additional analyses on the primary outcome

The beneficial effect of the intervention on the BioSecure score was confirmed independent of the degree of patients’ information about their treatments at baseline (difference: +6.2, 95% CI 1.3 to 11.1, p=0.0146).

The mean BioSecure score was higher for women than men (81.0 (13.0) vs 74.9 (14.2), p=0.0215) and was slightly negatively correlated with age (r=−0.24, 95% CI −0.40 to −0.06). The score was not correlated with disease duration (r=0.04, 95% CI −0.15 to 0.22) and did not differ by socioprofessional status or type of IA (data not shown).

### Secondary outcomes

Both groups showed improvement in all secondary outcome measures (table 3), but the BMQ necessity score increased only slightly. Coping was significantly more improved in the intervention than in the control group (p=0.0275), but the groups did not differ in the other outcome measures. One patient in the control group had a severe infection requiring hospitalisation (febrile enteritis) and none in the intervention group.

### Post-hoc analyses

BioSecure subscores concerning infections, adherence-related behaviours and vaccines were higher for the intervention than for the control group (table 4), particularly willingness for influenza vaccine (+23.7%, 95% CI 8.2 to 39.3) or tetanus vaccine (+20.3%, 95% CI 1.4 to 39.2) (online supplemental eAppendix 2 in online supplemental material 4) and willingness to pursue bDMARDs in case of remission. The subscores did not differ between groups for child conception or surgery. No centre effect was found; the primary outcome results were similar with a +5.6% difference (95% CI 1.10% to 10.19%, p=0.0153).

10.1136/rmdopen-2021-001828.supp2 Supplementary data

**Table 4:** BioSecure questionnaire subscores at 6 months

Subscore variables*	Control group (n=63)	Intervention group (n=64)	Absolute difference IG–CG (95% CI)
	n=59†	n=59†	
Adherence-related score (0–3), n (%)			
No correct answer	3 (5.1)	2 (3.4)	−1.7 (−11.2 to 7.2)
1 correct answer	10 (16.9)	4 (6.8)	−10.2 (−23.2 to 1.9)
2 correct answers	24 (40.7)	16 (27.1)	−13.6 (−30.3 to 3.7)
3 correct answers	22 (37.3)	37 (62.7)	25.4 (5.6 to 42.9)
Child conception score (0–2), n (%)			
No correct answer	24 (40.7)	21 (35.6)	−5.1 (−22.6 to 12.7)
1 correct answer	15 (25.4)	14 (23.7)	−1.7 (−17.6 to 14.2)
2 correct answers	20 (33.9)	24 (40.7)	6.8 (−10.9 to 24.2)
Drug storage/cold chain maintenance score (0–2), n (%)			
No correct answer	2 (3.4)	2 (3.4)	0 (−8.7 to 8.7)
1 correct answer	21 (35.6)	13 (22.0)	−13.6 (−30.1 to 3.3)
2 correct answers	36 (61.0)	44 (74.6)	13.6 (−3.6 to 30.1)
Infection score (0–17), mean (SD)	12.9 (3.0)	14.1 (2.7)	1.17 (0.13 to 2.21)
Surgery and dental care score (0–9), mean (SD)	7.5 (1.7)	8.0 (1.4)	0.53 (−0.03 to 1.08)
Vaccine score (0–5), mean (SD)	3.4 (1.6)	3.9 (1.3)	0.54 (0.00 to 1.08)

Scores are the number of correct answers in each skill type.

*Higher score indicates better score.

†Number of available data.

CG, control group; IG, intervention group.

### Feasibility

The mean (SD) intervention duration was 65 (17) min at baseline and 44 (19) min at 3 months. The education session was fully carried out in 98% of patients, according to the nurses’ opinion (online supplemental eAppendix 3 in online supplemental material 4).

## Discussion

In this multicentre randomised controlled trial, a nurse-led education intervention in addition to usual consultation by the rheumatologist when introducing a first subcutaneous bDMARD significantly enhanced patients’ competencies in terms of preventable safety matters assessed by a validated outcome measure. Willingness for vaccinations was higher in the intervention than in the control group, as were skills related to infections and adherence-related behaviours. The duration of the intervention was not lengthy: a 1-hour mean duration at baseline and 45 min 3 months later.

Prevention of bDMARD adverse effects is an important issue and is usually addressed by recommendations and training of rheumatologists and HCPs.^13 39^ Published interventions, including nurse-led programmes, have focused on improving providers’ prescription of vaccines.^26 27 40^ However, apart from vaccinations, HCPs’ compliance with safety recommendations to manage situations at risk is not known. Moreover, usual infectious complications are managed in primary care and the number of patients with IA encountered by a single GP is low, approximately one new case of RA annually.^41^

Therefore, this study aimed to increase patients’ awareness of the risks of bDMARDs to help them make their own decisions about whether or not to contact their rheumatologist and to give them the knowledge to inform their GP or other HCPs so they can handle these risks. The increased willingness to be vaccinated is also an interesting result because French people are known to be reluctant to be vaccinated, with vaccine hesitancy found among patients, HCPs and GPs.^42–44^

Considering secondary outcomes, patients’ degree of coping with their IA was improved by the intervention. Coping measurement by an NRS is part of the validated RAID score, has been used in clinical trials^45^ and has been found reliable and sensitive to change.^46^ Improved coping has been described in other education interventions.^45^ Some qualitative studies have shown that nurses are more ‘easy to talk to’.^47^ This may have allowed patients to express their views and to perceive the empathy shown by the nurse, thus contributing to better coping.

Disease activity scores and psychological well-being scores were improved in both groups as a result of the powerful effect of bDMARDs. The BMQ necessity score was particularly high at baseline in these patients who had agreed to initiate bDMARDs. This finding may explain why the intervention had no additional effect on this outcome. Similarly, the BMQ concerns score and the AHI, which were moderate at baseline, slightly decreased (improved) in both groups.

The strengths of this study include the multicentre design, the use of a validated primary outcome measure and the low rate of patients lost to follow-up. Particular attention was paid to reducing the bias inherent in an open trial of a non-pharmacological intervention. However, we did not eliminate all biases; because the rheumatologists in hospitals were informed at baseline of the randomisation group, we are not sure whether they did not compensate for this by delivering additional information to the control group, thus leading to lower differences in outcomes. The input of patients in the protocol by a patient association representative was also valuable. In addition, the intervention was delivered by nurses trained in patient education, with assessment of patients’ needs by a consensus procedure. Standardised safety messages were delivered and self-assessment of the compliance with the protocol was checked. These precautions may have lessened the risk due to many different settings and persons involved since no centre effect was found. This can allow the trial’s replication by other teams interested in patient education.

The first limitation concerns the choice of the primary endpoint. Considering the aim of the study, the ideal primary endpoint would have been to compare the number of severe infections in both groups. In this study, only one severe infection occurred in the control group and none in the intervention group. However, because the severe infection rate related to bDMARDs is low and the impact of patient education in this area had not been investigated to date in controlled trials, we chose to first assess the surrogate marker of patients’ skills evaluated by the validated BioSecure questionnaire. Although no cut-off is available to interpret the BioSecure score, our results are consistent with non-controlled studies. A survey performed in France showed lower safety skills related to bDMARDs in patients who had not benefited from patient education or a consultation with a nurse^38^ and retrospective uncontrolled studies had similar findings.^48^ Another controlled trial had similar results on patients’ safety abilities after a pharmacist-led intervention.^49^

Another limitation is that patients in the control group had received significantly less information compared with those in the intervention group who had two more sessions with the nurse. However, the aim of the study was to determine the additional benefits of a nurse-led education in a real-life bDMARD safety management setting in which rheumatologists only have time to provide brief information. Other limitations include a potential cultural bias, because the trial was conducted only in France, and a recruitment bias; patient education was already delivered in routine care by the recruiting centres, so there was a risk of excluding patients considered at high risk of adverse effects, low literacy,^50^ low beliefs related to medication or low self-management abilities. By perhaps excluding patients who, in the opinion of rheumatologists, could not do without safety education, this recruitment bias may have underestimated the results in the intervention group and may explain why, although the results for the primary outcome were significantly in favour of the intervention, we could have expected a greater difference between both groups due to the inclusion of patients who were bDMARD-naïve.

In conclusion, this randomised trial represents a significant advance in the field of safety management by showing a beneficial effect of a nurse-led intervention to increase patients’ safety skills related to bDMARDs. Hence, delivering a patient education session may be useful to patients starting a first bDMARD. Other studies will be necessary to assess whether the rate of severe adverse events is lowered by such an intervention.

## Data Availability

Data are available upon reasonable request by contacting the principal investigator (CB; catherine.beauvais@aphp.fr) and/or the data manager (AR; alexandra.rousseau@aphp.fr). Deidentified participant data will be available upon request by contacting the principal investigator (CB; catherine.beauvais@aphp.fr) and/or the data manager (AR; alexandra.rousseau@aphp.fr). Reuse of data is permitted with a signed data access agreement.
