# Supplementary material for: Efficacy of a nurse-led patient education intervention in promoting safety skills of patients with inflammatory arthritis treated with biologics: a multicentre randomised clinical trial
Source: RMD Open. 2022 Mar 16;8(1):e001828. doi: 10.1136/rmdopen-2021-001828 (PMC8928395; doi:10.1136/rmdopen-2021-001828)
Supplement: Supplementary data [file rmdopen-2021-001828supp001.pdf]

Supplementary Material 1. Protocol

eAppendix 1. First version of the protocol (17.02.2016)

ASSISTANCE PUBLIQUE HÔPITAUX DE PARIS

Efficacy of a nurse-led patient education intervention in promoting safety skills of patients with inflammatory arthritis treated with biologics.

BIOSAFE

Research in Routine Care

Version N°1 of 02/17/2016

Project Code: K151201 / BCR ID No.: 2016-A00332-49

Principal Investigator: Dr. BEAUVAIS Catherine
Rheumatology Department
Saint Antoine Hospital
Tel: + 33 1 49 28 25 20
Fax : + 33 1 49 28 25 13
Email: catherine.beauvais@aphp.fr

Scientific Director: Pr GOSSEC Laure
Rheumatology Department
Pitié Salpêtrière Hospital
Tel : + 33 1 42 16 00 00
Fax : + 33 1 42 17 79 55
Email: laure.gossec@aphp.fr

List of physicians/referrals from participating centers: see Appendix 1.

Associated teams :
Methodology and statistical analysis: Pr SIMON Tabassome, A. ROUSSEAU, Dr L. BERARD
Unit for Clinical Research (UCR) of the Groupe hospitalier de l'Est Parisien (UCR- East)
Saint-Antoine Hospital
184 rue du Faubourg Saint-Antoine, 75571 Paris cedex 12, France
Phone: + 33 1 49 28 22 02 / Fax: + 33 1 49 28 28 13
Email: alexandra.rousseau@aphp.fr / laurence.berard@aphp.fr
Structure in charge of research follow-up: TOUATI Amel
Unit for Clinical Research UCR of the Groupe hospitalier de l'Est Parisien (UCR-East)
Saint-Antoine Hospital
184 rue du Faubourg Saint-Antoine, 75571 Paris cedex 12, France
Phone: + 33 1 49 28 22 02 / Fax: + 33 1 49 28 28 13
Email: amel.touati@aphp.fr

Study Manager: Aurélie ZINDJIRDJIAN
Tel : 01 44 84 17 64 / Fax : 01 44 84 17 01

Beauvais C, *et al. RMD Open* 2022; 8:e001828. doi: 10.1136/rmdopen-2021-001828

Email: aurelie.zindjirdjian@aphp.fr
Delegation for Clinical Research and Innovation
Saint Louis Hospital 75010 PARIS

SIGNATURE PAGE OF A RESEARCH PROTOCOL IN Routine Care
by the COORDINATING investigator and the MANAGER's representative

Page de SIGNATURE D'UN PROTOCOLE de recherche en soins courants
par l'investigateur COORDONNATEUR et le représentant du GESTIONNAIRE

Code de la Recherche en soins courants : K151201, BIOSAFE

Titre : Efficacité d'une éducation thérapeutique par un(e) Infirmier (ère) par l'acquisition des compétences de sécurité vis-à-vis des biothérapies par les patients traités pour un rhumatisme inflammatoire chronique

Version N°1.1 du : 09/05/2016

La recherche sera conduite conformément au protocole et aux dispositions législatives et réglementaires en vigueur.

L'investigateur coordonnateur :
Dr BEAUVAIS Catherine
Service de Rhumatologie
Hôpital Saint-Antoine
Tél : 01 49 28 25 20
Courriel : catherine.beauvais@aphp.fr

Date : 2 / 9 / 2016
Signature :

Le responsable scientifique :
Pr GOSSEC Laure
Service de Rhumatologie
Hôpital Pitié-Salpêtrière
Tél : 01 42 16 00 00
Courriel : laure.gossec@aphp.fr

Date : 2 / 9 / 2016
Signature :

La directrice du DRCD :
Mme Florence Favrel-Feuillade
Assistance Publique – Hôpitaux de Paris
Délégation Interrégionale à la recherche clinique
Hôpital Saint Louis
75010 PARIS

Date : 08 NOV. 2016
Signature :
Serge BUREAU
PÔLE PROMOTION
DRCD - APHP

La recherche a reçu un avis favorable du CPP Ile de France VI en date du 22/06/2016

Version 1 of 17/02/2016

TABLE OF CONTENTS

1. ABSTRACT 4
2. SCIENTIFIC RATIONALE – RATIONALE FOR THE STUDY 7
2.1 Current state of knowledge with regard to research 7
2.2 Qualification of the research 7
2.2.1 Evidence that the medical strategies that are the subject of the research, the practical procedures and methods used in the research are consistent with current practice. 7
2.2.2 Evidence that the specific monitoring methods added by the research involve minimal risk and constraints. 8
2.3 Recruitment potential 9
2.4 Statement that the research will be conducted in accordance with the protocol, good clinical practice and applicable laws and regulations. 9
3. OBJECTIVES AND OUTCOME CRITERIA OF THE RESEARCH 9
3.1 Primary outcome. 9
3.2 Primary outcome measure 9
3.3 Secondary outcomes 10
4. SELECTION CRITERIA 10
4.1 Inclusion criteria 10
4.2 Exclusion criteria 10
5. RESEARCH DESIGN 11
5.1 Type of study 11
5.2 Expected number of participating centers 11
5.3 National/international research 11
5.4 Duration of the research 11
5.5 Description of intended measures to reduce and avoid biases 11
5.5.1 Randomization 11
5.5.2 Blinding and methods for binding maintenance, procedures for unblinding. 11
5.6 Rheumatology nurses training in patient education 11
5.7 Simultaneous participation in other research 12
6. PROGRESS OF THE RESEARCH 13
6.1 Scheme of the study 13
6.2 Experimental design - implementation 13
6.2.1 Selection visit 13
6.2.2 Inclusion visit (Baseline) 14
6.2.3 Research follow-up visit at 3 months 14
6.2.4 End-of-research visit 15
6.2.5 Summary table of patient follow-up 16
6.2.6 Management of examinations and samples 16
6.2.7 Building a biological collection 16
6.3 Rules for permanent or temporary cessation of research 17
6.3.1 Discontinuing an individual's participation in research 17
6.3.2 Discontinuation of part or all of the research 17
6.3.3 Methods and schedule for collecting data 17
6.3.4 Methods of follow-up 17
7. AUTHORIZED AND PROHIBITED DRUGS AND TREATMENTS 17
8. DATA MANAGEMENT 17
8.1 Access right to data and documents sources 17
8.1.1 Data Access and Confidentiality 17
8.1.2 Source documents and data 18
8.2 Quality control and quality assurance 18
8.2.1 Guidelines for data collection 18
8.2.2 Quality Control 18

8.2.3 Audit......*19*
8.3 Data processing and conservation of documents and research data......19
8.3.1 Data processing......*19*
8.3.2 Retention of Research Documents and Data......*19*
9. STATISTICS......19
9.1 Justification of sample size......19
9.2 Description of the planned statistical methods, including the schedule of planned interim analyses.....20
9.3 Consideration of missing data......21
9.4 Populations for analysis......21
10. MANAGEMENT OF ADVERSE EVENTS......22
10.1 Adverse events (i.e., complications) related to medical procedures, combinations of procedures or prevention strategies, diagnosis or treatment that are part of the routine care (excluding medication).......22
10.1.1 Adverse events (i.e. complications) related to the care evaluated in the research......*22*
10.1.2 Circuit for reporting adverse events (i.e. complications) related to care......*22*
10.2 Adverse events with special follow-up by the manager......22
10.3 Adverse events related to special monitoring added by research......22
11. PERSONS INFORMATION METHODS......22
11.1 Information of the person.......22
12. ETHICAL AND LEGAL ASPECTS......22
12.1 Declaration that the research will be conducted in accordance with the protocol, current laws and regulations.......22
12.2 Ethical evaluation of the specific monitoring methods provided for in the protocol......23
12.3 Legal requirements (Role of the manager, CPP, CCTIRS, CNIL)......23
12.3.1 Role of the manager......*23*
12.3.2 Submission to the CPP......*23*
12.3.3 Notice from CCTIRS and authorization from the CNIL......*23*
12.3.4 Substantial modification to the protocol......*23*
12.3.5 Final research report or publication......*24*
12.3.6 Data ownership......*24*
12.3.7 Publication rules......*24*
13. BIBLIOGRAPHICAL REFERENCES......24
14. APPENDICES......27
14.1 Appendix 1: List of participating centers in June 12, 2017......27
14.2 Appendix 2: Questionnaires......29
14.2.1 BioSecure Questionnaire......*29*
14.2.2 SF 12......*33*
14.2.3 MMAS Morisky modified adherence scale. BMQ......*35*
14.2.4 RAID: Rheumatoid Arthritis Impact of Disease......*35*
14.2.5 Arthritis helplessness index......*37*
14.2.6 Disease activity score DAS 28......*38*
14.2.7 BASDAI Bath Ankylosing Spondylitis Disease Activity Index......*38*
14.2.8 ASDAS......*39*
14.3 Appendix 3: Nurse Education Booklet......40

1. ABSTRACT

Introduction: Chronic inflammatory rheumatic diseases (inflammatory arthritis [IA]) (rheumatoid arthritis (RA) and spondyloarthritis (SpA) are serious and disabling conditions that affect approximately 600,000 people in France. The prognosis of IA has been substantially improved by biologic disease-modifying anti-rheumatic drugs (bDMARDs), such as anti-TNF alpha, but these bDMARDs can have

serious complications, particularly infections of the upper respiratory tract and pneumonia, with risk of tuberculosis and rare cases of opportunistic infections. The risk of serious infections is 5% per patient-year and is maximal in the first 6 months after the prescription of a first bDMARD. Therapeutic patient education (TPE) enables patients to acquire safety skills for managing infectious risks (e.g., stopping biologic therapy in case of fever). Skills acquisition is evaluated by the validated “BioSecure” questionnaire. Patient education on bDMARDs is mainly carried out by nurses and seemed to be effective in the acquisition of these skills in 2 uncontrolled studies. In 2010, a national survey of 677 patients showed that risk of giving wrong answers in the BioSecure questionnaire was 4-fold greater for patients who did not have TPE than those who had benefited from a consultation with a nurse or an educational approach (OR=3.8 95% CI: [1.68- 8.8]).

Hypotheses: Our hypothesis is that a face-to-face nurse-led patient education will allow for better acquisition of safety skills regarding bDMARDs at 6 months as compared with usual care (medical information during consultation at the time of bDMARD prescription).

Primary outcome: Acquisition at 6 months by IA patients of safety skills with regard to subcutaneously injected bDMARDs.

Secondary outcomes: Effect at 6 months of the nurse-led education on quality of life, disease activity, “coping” with the disease, psychological well-being, occurrence of serious infections related to treatment.

Primary outcome measurement: Response rate to the BioSecure validated questionnaire comprising 55 items, developed by the Patient education Group Section of the French Rheumatology Society, including safety skills concerning infections, vaccinations, and everyday life situations (travel, surgery, child conception etc.).

Secondary outcome measures: Adherence : MMAS 4 Modified Morisky Adhrence scale Quality of life: SF-12; rate of severe infections defined as infections requiring hospitalization or intravenous antibiotics; coping with disease and psychological well-being (numeric rating scales) adapted from Rheumatoid Arthritis Impact (RAID) and Arthritis Helplesness Index (AHI); Disease Activity Score, ASDAS and BASDAI activity index.

Methods: Type of study and experimental design: Randomized open-label randomized controlled trial with blinded assessment.

The protocol will be proposed by the rheumatologist during a routine consultation when the decision is made to start bDMARDs. The self-administered questionnaire will be completed after receipt of non-opposition to the survey and before randomization.

Intervention group: usual consultation and information by the doctor + 2 TPE sessions by a trained nurse at a 3-month interval and follow-up by the treating rheumatologist. The first TPE session at baseline (BL), lasting a maximum of 1.5 hr, will include evaluation of the patient’s experience and knowledge, internal and external resources to deal with the disease and treatments, teaching of the subcutaneous (SC) self-injection, what to do in case of fever, and adjusting bDMARD treatment in risk situations. At 3 months, the second TPE session will include adaptation of educational messages to the patient’s feedback and acquisitions. The TPE intervention will be standardized by consensus and a brochure will be provided based on the results of the BioSecure study.

Control group: usual consultation and information from the doctor and follow-up by the treating rheumatologist.

The primary outcome (BioSecure score) and self-administered questionnaire data will be assessed at the end of the study at 6 months in all patients during a consultation at the hospital by a health professional different from the nurse who has performed patient education. A TPE session will be offered at 6 months apart from the protocol for patients in the control group.

Legal characteristics of the project: research in routine care

Number of patients needed: 120

Inclusion criteria: Age 18 to 75 years,

Rheumatoid arthritis (ACR/EULAR criteria) or axial or peripheral spondyloarthritis (ASAS criteria) including psoriatic arthritis. Indication for a first subcutaneous bDMARD (according to the current French recommendations for IA management), patients bDMARDS naïve,

Ability to complete a questionnaire, collection of non-opposition, beneficiary of a social security.

Exclusion criteria:

Severe psychiatric disorders or cognitive impairment.

Total study duration: 18 months (12 months inclusion and 6 months patient participation)

Inclusion period: 12 months

Length of participation for one patient: 6 months

Number of participating centers: 10

Average number of inclusions per month per center: 1

2. SCIENTIFIC RATIONALE – RATIONALE FOR THE STUDY

2.1 *Current state of knowledge with regard to research*

Chronic inflammatory arthritis (IA) (rheumatoid arthritis [RA] and spondyloarthritis [SpA]) affect approximately 600,000 people in France. When insufficiently controlled by treatment, these painful and disabling conditions affect quality of life and ability to work, eliminating patients from employment. bDMARDs (biological disease-modifying anti-rheumatic drugs) are highly effective treatments for IA and are increasingly used. In 2010, an estimated 30% IA [1] patients in France were treated with bDMARDs, mainly with TNF alpha blockers administered in SC injections. The bDMARDs' treatment rate in IA population is certainly higher nowadays. However, bDMARDs are at risk of serious complications, particularly infections, such as pulmonary infections, tuberculosis and some cases of opportunistic infections [2]. The risk of infections related to bDMARDs is estimated to 2-folds risk vs conventional DMARDs [2]. The risk of severe infection is estimated at 5%/patient-years and is highest in the 6 months following the first bDMARD [3].

Therapeutic patient education (TPE) is recommended to help patients acquire specific skills to better manage their chronic disease in everyday life [4]. TPE is also recommended in the management of IA [5,6]. Among the required skills are safety skills aimed at preserving the patient's life by avoiding complications termed 'life-saving self-care skills' [4]. Safety skills can be assessed by the validated BioSecure questionnaire, a 55-item questionnaire assessing patients' skills in managing risk situations: fever, infections, vaccinations, travel, surgery, pregnancy [7]. The BioSecure questionnaire is also widely used in routine care in the management of IA. TPE on bDMARDs seems to be effective on safety skills in one uncontrolled (abstract) study [8], while another uncontrolled study showed results in favor of group TPE [9]. Moreover, in 2010 a national survey showed that the number of wrong answers to the BioSecure questionnaire was 4-folds higher in patients who did not benefit from TPE (at least a consultation by a nurse) (OR=3.8 IC95% [1.6-8.8]) [10,11]. In this 2010 study, only 30% of patients had received a nurse-led TPE and 11% had access to TPE sessions [12].

Since 2010, the number of nursing consultations dedicated to IA has increased (see below) but no study has evaluated the benefit of this approach on safety skills.

In this context, the aim of this single-blind, multicenter, randomized trial is to evaluate the impact of an education by a nurse on safety skills with regard to bDMARDs. Our hypothesis is that the education group will have better skills at 6 months than the control group.

2.2 *Qualification of the research*

This protocol is part of a research project aimed at evaluating routine care as defined by law n°2004-806 of August 9, 2004 relating to public health policy and by its application decree (n° 2006-477) of April 26, 2006. (Reference texts: articles L.1121-1, 2nd paragraph and R1121-3 of the Public Health Code).

2.2.1 Evidence that the medical strategies that are the subject of the research, the practical procedures and methods used in the research are consistent with current practice.

TPE is recommended in the management of chronic diseases [4] such as IA, and specifically by the French Rheumatology Society [5,6] for rheumatoid arthritis and spondyloarthritis. Although TPE is still

insufficiently developed [10], IA patients are frequently included in TPE rheumatology programs in France. Indeed, of the 165 TPE programs authorized in rheumatology and listed in 2012 by Regional Health Agencies, 80 were targeted to IA patients [12]. However, evaluation of the efficacy of these programs has not been performed to date and is one of the barriers to the development of TPE for these patients.

The TPE nurse-led consultation usually lasts a maximum of 1.5 hours and follows the medical decision for a bDMARD and initial information by the doctor on benefits/risks of the treatment, the expected positive effects and the main adverse events. TPE is usually carried out by trained nurses.
The consultation by the nurse, usually done with a semi-structured interview schedule, uses a framework in accordance with the recommendations of the High Health authority:

- Evaluation of the patient's experience and knowledge of his/her illness.
- His/her internal and external resources to deal with the disease.
- Information and education on bDMARDs including teaching self-injections and safety warning signs.
- If the patient does not feel able to perform self-injection, the nurse will suggests that the doctor prescribes a nurse in private practice for the first injections.

In routine care, bDMARDs are prescribed by the rheumatologist during the consultation in hospital. The rheumatologist provides information on the treatment benefits/risks, on the expected positive effects and the main side effects and warning signs. The rheumatologist prescribes the first injections by a nurse if necessary.

After initiation of the bDMARD, the patient is routinely followed-up by the treaty physician at 3 months for evaluation of treatment efficacy according to EULAR (European Alliance of Associations for Rheumatology) response criteria. In case of efficacy, the treatment is continued until 6 months when a new evaluation is performed. In case of insufficient response or non-response, a switch to another bDMARD is provided at 3 months.

2.2.2 Evidence that the specific monitoring methods added by the research involve minimal risk and constraints.

As part of the proposed research, will be added :

- one TPE consultation by a nurse at baseline (BL) and at 3 months in the intervention group
- the filling-up in of self-administered questionnaires in both groups at BL and at 6 months.

All of the nursing consultations and self-administered questionnaires filling-up will be carried out during the usual consultations scheduled for these patients as part of the follow-up of their disease according to the recommendations of the French Rheumatology Society [5,6].

**Monitoring procedures in the usual care framework**	**Monitoring procedures <u>added by the research</u>** (Additional procedures compared to the usual care)
-Baseline : medical clinical examination<br>-3 months : clinical and biological monitoring examination with regard of bDMARDs by the treating rheumatologist	-In the intervention group: TPE consultation by a nurse at baseline and 3 months.<br>-In both groups: filling-in of self-administered questionnaires at baseline and 6 months.

-6 months: clinical and biological monitoring examination with regard of bDMARDs by the treating rheumatologist<br>-	

Table 1: Specific monitoring modalities carried out as part of usual care / added by research

2.3 *Recruitment potential*

Last name, First name	Country, City, Hospital	Specialty	Recruitment / month	Total	**Center number**
Catherine Beauvais	France, Paris, CHU Saint Antoine	Rheumatology	*1*	*12*	**001**
Laure Gossec	France, Paris, CHU Pitié Salpétrière Hospital	Rheumatology	*1*	*12*	**002**
Françoise Fayet<br>Martin Soubrier	France, Clermont-Ferrand, CHU Gabriel Montpied Hospital	Rheumatology	*1*	*12*	**003**
Laurent Grange	France, Hopital Sud Echirolles, CHU Grenoble	Rheumatology	*0.5*	*6*	**004**
Anne-christine Rat	France, Nancy, CHU Brabois	Rheumatology	*0.5*	*6*	**005**
Yves Maugars<br>Marie Pierre Aubert	France, Nantes, CHU	Rheumatology	*1*	*12*	**006**
Aleth Perdriger	France, Rennes, CHRU Pontchaillou	Rheumatology	*1*	*12*	**007**
Sophie Pouplin	France, Rouen, CHU University Hospital of Rouen,	Rheumatology	*1*	*12*	**008**
Christelle Sordet.	France, Strasbourg, CHU Strasbourg	Rheumatology	*1*	*12*	**009**
Béatrice Pallot Prades	France, Saint Etienne, CHU Saint Etienne , Bellevue Hospital	Rheumatology	*1*	*12*	**010**
Total			10	120	

2.4 *Statement that the research will be conducted in accordance with the protocol, good clinical practice and applicable laws and regulations.*

I, the undersigned, Dr Catherine BEAUVAIS, certify that the research that I will coordinate, will be conducted in accordance with the protocol, the good clinical practices and the legislative and regulations in France.

3. OBJECTIVES AND OUTCOME CRITERIA OF THE RESEARCH

3.1 *Primary outcome.*

Evaluate the efficacy at 6 months of a nurse-led education session on the acquisition of safety skills by IA patients regarding bDMARDs.

3.2 *Primary outcome measure*

Safety skills measured at 6 months by the BioSecure validated questionnaire comprising 55 items, developed by the therapeutic education Section of the French Society of Rheumatology.
This questionnaire assesses safety skills related to infections, vaccinations, and everyday life situations (travel, surgery, child conception) [Appendix].

3.3 Secondary outcomes

1) Secondary outcomes
    - Efficacy of TPE with regard of to the official objectives of the High Health Authority that is quality of life, adherence
    - Severe infections related to treatment
    - "Coping" with the disease, psychological well-being
    - Correlations between gained skills and patient characteristics

2) Outcome measures

    - Adherence to Biologics assessed at 6 months by the MMAS 4 Modified Morisky Adherence [14]
    - Quality of life: difference between baseline and 6 months of MOS-SF-12 questionnaire SF 12 [15]
    - Severe infections rate occurring within 6 months of initiation of bDMARDs, defined as infections requiring hospitalization or intravenous antibiotics.
    - Coping with disease: difference between baseline and 6 months in coping (from Rheumatoid Arthritis Impact of Disease [RAID]) measured by a numeric rating scale (NRS) [16] and the Arthritis Helplessness Index (AHI) [17].
    - Psychological well-being: difference between baseline and 6 months in psychological well-being (from RAID) measured by an NRS [16]
    - Association between responses to the BioSecure questionnaire and patient characteristics: sex, age, education level, disease duration, underlying disease (RA, axial SpA).
    - Opinions regarding the treatment evaluated by the Beliefs About Medicines Questionnaire (BMQ) [26].

4. SELECTION CRITERIA

4.1 Inclusion criteria

- Age from 18 to 75 years
- RA (fulfilling the 2010 American College of Rheumatology [ACR]/ European League Against Rheumatism [EULAR] classification criteria ACR/EULAR criteria) or axial or peripheral SpA (fulfilling the 2009 Assessment of SpondyloArthritis international Society classification [ASAS] criteria) [24,25]
- Indication for a first subcutaneous bDMARD (according to the usual French recommendations of care), therefore bDMARD-naïve
- Ability to complete a questionnaire
- Collection of non-opposition to participation in the study
- Beneficiary of social protection or an entitled person

4.2 Exclusion criteria

- Any condition that may affect understanding or adherence to treatment (chronic alcoholism, language barrier, severe psychiatric disorders)
- Intension for intravenous bDMARDs
- Already received therapeutic education about bDMARDs

5. RESEARCH DESIGN

5.1 Type of study

Randomized, multicenter, open-label, study with blinded evaluation of the primary outcome.

5.2 Expected number of participating centers

Multicentric. 10 participating centers.

5.3 National/international research

National Research

5.4 Duration of the research

Total study duration: 18 months
Inclusion period: 12 months
Participation duration for one patient: 6 months

5.5 Description of intended measures to reduce and avoid biases

5.5.1 Randomization

The unit of randomization will be the patient. The randomization will be carried out during the medical consultation, after the collection of the non-opposition and after the filling-in of the self-administered questionnaires, by Internet via the Cleanweb software.
Randomization will be stratified by center and balanced by blocks that will not be communicated to the investigating team. The ratio will be 1:1.
A randomization number will be assigned (consisting of 2 random letters and 6 random numbers) will be reported in the CRF (case report from).

5.5.2 Blinding and methods for binding maintenance, procedures for unblinding.

Due to the type of intervention, double-blinding will be impossible. However, patients in the intervention group will not be in contact with the education nurse during the 6-month assessment to ensure that the primary outcome is blinded to the randomization arm.

5.6 Rheumatology nurses training in patient education

In order to ensure that all nurses have the experience and qualifications required to conduct the TPE sessions in accordance with the High Health Authority and French Society of Rheumatology recommendations, it has been ensured that at least 1 nurse from 8/10 of the participating centers has a university degree in TPE. This degree in TPE for osteoarticular diseases (Université Pierre et Marie Curie - Paris VI) was developed by the TPE Section of the French Society of Rheumatology. Other nurses will have a TPE training of at least 40 hours.
In addition, all investigating centers will prove an authorization to conduct TPE programs for chronic inflammatory rheumatic diseases, delivered by Regional Health Agencies.

This will ensure TPE standardization among the participating centers.

In addition, a semi-structured interview brochure will be provided to all nurses. This brochure is derived from the semi-structured schedule questionnaire called "de Rouen" developed by the Rheumatology Department of the Rouen University Hospital and widely used.

5.7 Simultaneous participation in other research

Participation in non-interventional patient research is possible.
There is no exclusion period for participation in intervention research after the end of participation in the Biosafe study.

6. PROGRESS OF THE RESEARCH

6.1 Scheme of the study

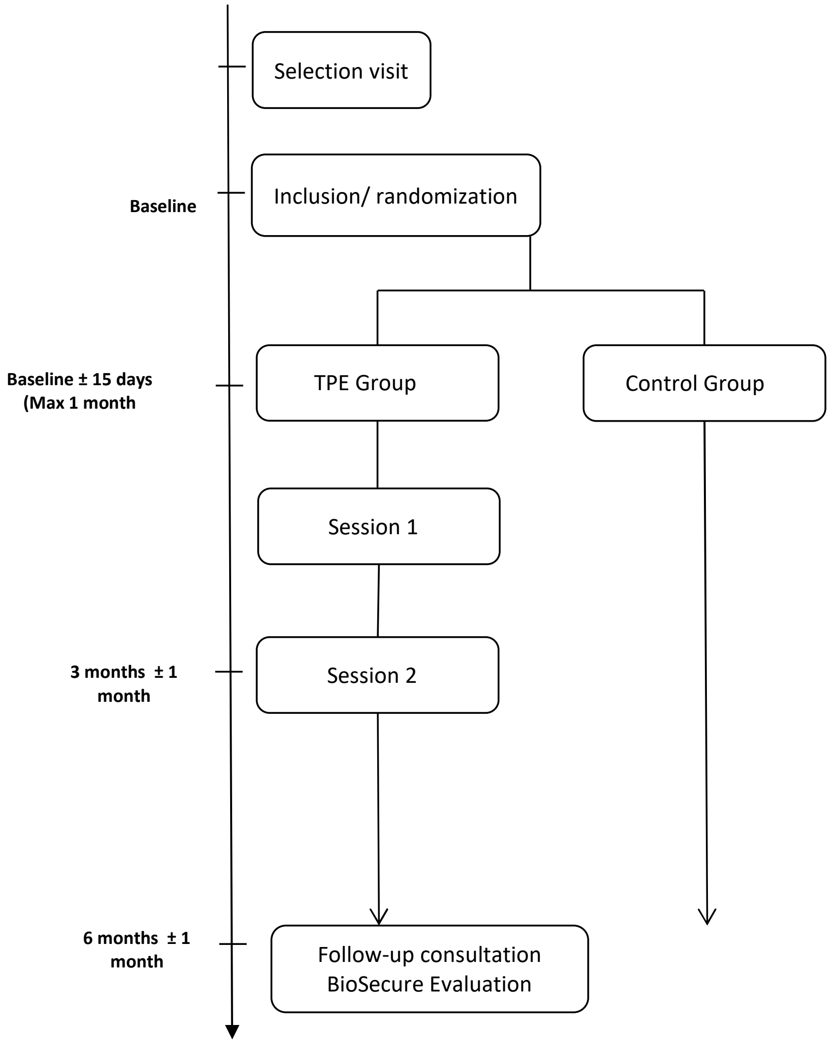

6.2 Experimental design - implementation

6.2.1 Selection visit

The pre-inclusion visit will take place during a medical consultation, i.e. either a regular consultation or a consultation during a day care hospitalization known as a "pre-biologic check-up" carried out by many investigating centers.

During the pre-inclusion visit, the physician will check the eligibility criteria (inclusion and exclusion criteria), propose to participate in the study and provide the written information note about the protocol. At the pre-inclusion visit, the physician will ensure that the health check-up for bDMARDS is scheduled for the bDMARD initiation at baseline. Otherwise, the physician will prescribe a biologic sample including at least Erythrocyte sedimentation rate and C reactive protein dosage for the baseline visit.

At baseline, Erythrocyte Sedimentation Rate and C reactive protein results will be collected, dating from less than 1 month. If not available, these dosages will be performed within 1 week, according to usual care before the initiation of a bDMARD.

6.2.2 Inclusion visit (Baseline)

The baseline inclusion visit will take place at the time of or after the pre-inclusion visit depending on the department's procedures and the patients' preferences.
In addition to the usual clinical examination, the inclusion visit will include:

- Information by the physician on bDMARD and safety according to standard care.
- Patient's non-opposition after checking of the inclusion and exclusion criteria, particularly the absence of counterindications to bDMARDs according to the results of biological sample less than one month old.
- Patient completion of self-administered questionnaires

The patient will then be randomized (via the Cleanweb software) into one of 2 groups: Intervention Group or Control Group.

The rheumatologist will then prescribe, as usual, bDMARD and the usual biological follow-up of bDMARDs, systematically including Erythrocyte sedimentation rate and C reactive protein dosage at 6 months.

Depending on the patient's randomization group, information on bDMARD will be different.

- Intervention Group

The nurse's intervention at baseline will take place on the day of inclusion or within 2 weeks after the inclusion visit, maximum 4 weeks later.
The baseline education session will last a maximum of 1h30. It will be standardized between centers and will include the following:
-Patients' evaluation using a semi-structured questionnaire and interview.
-Messages to deliver about the safety of bDMARDs and appropriate behaviors in everyday situations.
-Motivation for bDMARDs and potential difficulties
-Sub-cutaneous injection only according to the centers' procedures. If the nurse concludes that the patient will not be able to perform self-injections, he/she will suggest a prescription for a nurse in private practice.

- Control Group

There will be no nurse intervention. A prescription for a nurse in private practice will be made according to the physician's habits, which will be collected in the Case report form (CRF) at 3 months.

6.2.3 Research follow-up visit at 3 months

All patients will be seen at 3 months according to usual care recommendations including monitoring of potential adverse events.
In the intervention group a nursing interview will be conducted (TPE at 3 months).
The visit at 3 months will be performed 3 months (± 15 days) after the baseline visit.
Education session will be standardized between centers and will include the following

- Free collection of the patient's experience and difficulties in the past 3 months.
- Review and reassurance of security messages
- Motivation enhancing

The intervention will be face-to-face. However, in case of repeated refusal by the patient for a face-to-face interview and close to the 4-month deadline, the visit at 3 months will be done by telephone call with the patient's consent.
If possible, the interview at 3 months will be done by the same nurse as at baseline.

6.2.4 End-of-research visit

The 6-month visit will concern both groups. It will take place at 6 months (±1 month) after inclusion.
In addition to the usual-care clinical examination, it will include:
- Completion of self-administered questionnaires by all patients. The questionnaires will be given to the patient by a member of the team blinded to the patient's randomization arm, i.e. a health professional other than the nurse and doctor seen at baseline and 3 months; the health professional can be, for example, another nurse, another doctor, etc. A check that all the items in the Biosecure questionnaire have been fully completed will be done by the health professional collecting the questionnaire. If the patient does not meet the minimum safety skills at 6 months, another TPE session will be scheduled as part of the usual care.
- Collecting of other data will be done in the same way as well as self-administrated questionnaires.
At the end of this visit, the patient's participation in the study will end.

The participating centers will then offer, apart from the protocol, an education session on bDMARDs to patients in the control group and/or will at least assess the patient's knowledge on bDMARDs to offer this session to patients with insufficient knowledge. It will be possible to perform this session after the end-of-research visit, apart from the protocol, only after the 6-months assessment has been completed.

If the patient does not attend to the end-of-research visit, the missing data at 6 months will be collected by the investigators and will include the following:

- New appointment scheduled within 1 month
- Or sending questionnaires by mail
- And collecting of the C-reactive protein sample and DAS28 score from the treating rheumatologists because these data are available as part of usual-care follow-up

6.2.5 Summary table of patient follow-up

**Data collection**	**BL-1**	**BL**	**3 months**	**6 months**
**Visits**	**V0**	**V1**	**V2**	**V3**
	**SELECTION**	**INCLUSION Randomization**		**Evaluation**
**Protocol information**	R			
**Selection criteria**	R	R		
**Collection of the non-opposition form**		R		
**Randomization in both groups**		R		
Socio-demographic data<br>Current treatment<br><br>Biological examination (usual care)<br>ESR and CRP		 UC<br>UC<br><br>UC		 <br><br><br><br>UC
Clinical examination		UC		UC
Severe infections		UC (within the last 2 years)		UC (since BL)
**Self-administered questionnaires:**<br>overall level of information<br><br>Disease Activity<br>Patient opinion on disease activity for DAS 28 and<br>ASDAS calculation<br>BASDAI for all SpA		 <br><br><br>R<br>R<br>R		 <br><br><br>R<br>R<br>R
**BioSecure: primary outcome**<br>bDMARDs Injection by the patient her/himself				R<br><br>R
Adherence to biologics: MMAS 4<br>Quality of Life SF12 (Short form)<br>Coping NRS<br>Psychological well-being NRS<br>AIH<br>BMQ		 R<br>R<br>R<br>R<br>R		R<br>R<br>R<br>R<br>R<br>R

Abbreviations: BL, baseline; R, research ; US, Usual Care; ESR, erythrocyte sedimentation rate; CRP, C reactive protein; DAS28, Disease activity score 28 joints; ASDAS, ASAS-endorsed disease activity score ;BASDAI, Bath Ankylosing Spondylitis Disease Activity Index SF12, MOS SF12 ; NRS, numeric rating scale; AHI, Arthritis Helplessness Index; BMQ, Beliefs about medication questionnaire; MMAS modified Moritsky Adherence Scale

Table 2: Summary table of research timeline for the patient

6.2.6 Management of examinations and samples

The trial does not involve research-specific specimens; specimens collected for routine patient follow-up will be collected and routed through the usual channels at each center.

6.2.7 Building a biological collection

Not applicable

6.3 Rules for permanent or temporary cessation of research

6.3.1 Discontinuing an individual's participation in research

Any individual may withdraw prematurely his/her participation in the research at any time for whatever reasons.

The treating physician may temporarily or permanently discontinue a subject's participation in research for whatever reason in the best interests of the subject.

In case of the following:

- Temporary discontinuation from the research: the patient will continue the study, the physician following the patient will document the discontinuation reason.
- Definitive withdrawal from the research: the patient will continue the research follow-up, until his/her participation ends; the investigating center or the treating rheumatologist will document the termination reason.

6.3.2 Discontinuation of part or all of the research

The manager of AP-HP (Assistance publique hôpitaux de Paris) will reserve the right to permanently suspend inclusions, at any time, if the objectives are not met in terms of number of inclusions.

6.3.3 Methods and schedule for collecting data

The data collected will be analyzed

6.3.4 Methods of follow-up

Patients will continue follow-up according to the usual care by their treating rheumatologists.

7. AUTHORIZED AND PROHIBITED DRUGS AND TREATMENTS

The drugs authorized and prohibited in this research are the same as those used in the usual care for IA.

8. DATA MANAGEMENT

The data will be collected on a paper CRF in each center. Duplicates of the CRFs will be sent to the UCR-East as soon as the patient will have completed the study with an envelope provided to each center.

The CRFs will be sent grouped and the data will be entered by a Clinical Research Technician (CRT) in a CleanWeb Telemedicine database accessible via the internet and developed by a data manager of Unit for Clinical Research (UCR-East).

8.1 Access right to data and documents sources

8.1.1 Data Access and Confidentiality

The research manager will ensure that the protocol and the information form to persons included in the research state that:

- the documents and individual data are strictly necessary for the follow-up, quality control and audit of research aimed at evaluating routine care.
- these documents and data will be available only to persons individually mandated for this purpose by the research manager.

The research manager will also ensure that each person involved in the research has not objected to access to his/her individual data.

All research activities must obey strict rules of confidentiality. The persons mandated for the research are bound by professional secrecy, in particular under the conditions defined by articles 226-13 and 226-14 of the Penal Code, in the same way as the investigators themselves.

During and at the end of the research, individual collected data that will be transmitted to the manager by the investigators (or any other research collaborators) will be coded. In no case should they clearly show the name of the persons concerned, nor their address, nor any other information allowing direct identification.

8.1.2 Source documents and data

The self-administered questionnaires (including BioSafe) will be considered as source data as well as the patient's medical record.

8.2 Quality control and quality assurance

8.2.1 Guidelines for data collection

All information required by the protocol must be recorded into the CRFs. These data must be collected and recorded as soon as they are obtained, and transcribed into the CRF in an accurate, complete and legible way.
Each missing data must be explained. Erroneous data found in the CRF will be clearly crossed out and the new data will be copied, next to the crossed-out information, with initials, date and possibly a justification by the person conducting and monitoring the research or the authorized person who made the correction.

Paper CRF

All the information required by the protocol will be provided into the CRF and an explanation will be given by the physician for each missing data. Data will be transferred into the CRF as they are obtained, whether clinical or paraclinical.
The data will be copied neatly and legibly with a black ballpoint pen into the CRFs to facilitate duplication and computer input.
Incorrect data found in the CRF will be clearly crossed out and the new data will be copied with initials and date by the research team member who will make the correction.
Subjects' anonymity will be ensured by a code number and the subject's initials will be ensured in all documents needed by the research, or by deleting by appropriate means nominative data from source documents.
The computerized data on file will be declared to the French data protection authority (Commission Informatique et libertés (CNIL)) according to the current procedures.

8.2.2 Quality Control

The investigator will make the documents and individual data strictly needed for the monitoring, quality control and audit of this research available to the persons in charge of quality control and duly mandated by the research manager.

The person(s) mandated by the research manager will regularly visit each center, during the research implementation, one or more times during the research according to the rhythm of inclusions and at the end of the research.

During these visits, the following items will be collected:

- Subjects' protection and safety,

- compliance with the research protocol and procedures defined therein and by the current regulatory legislation.
- data quality collected in the CRF: accuracy, missing data, data consistency with source documents (medical records, appointment books, original laboratory results, etc.),
- management of products and samples.

All visits will be monitored with a written report.

8.2.3 Audit

An audit will eventually be carried out at any time by persons mandated by the manager independently of the research investigators. Its purpose will is to ensure the research quality, the results validity and compliance with the current regulatory legislation.

Those conducting and monitoring the research will agree to comply with requirements from the manager and authorities and will agree with an audit or inspection.

The audit can happen at all stages of the research, from protocol development to results' publication and data classification used or produced by the research.

8.3 Data processing and conservation of documents and research data

8.3.1 Data processing

Clinical data will be collected at each visit by the patient's physician in a paper CRF.
The CRF duplicate and questionnaires' copy will be mailed to the UCR-East) for data entry. The data will be entered into a CleanWeb database by a Clinical Research Technician (CRT) in the URC-East.

Data management (creation of the database on CleanWeb and data control according to a predefined validation planning via the SAS software) will be carried out by a URC-Est data manager.

The patient will be identified by the inclusion center number, his/her inclusion number and initials. The patient reference will be: center number - center inclusion number - last name initial - first name initial (|__|__|__|-|__|__|__|__|__|__|__|-S-N). (Surname, Name)

A randomization number will be assigned and reported in the CRF (consisting of 2 random letters and 6 random numbers).

Data processing will be under the responsibility of the UCR-East.

8.3.2 Retention of Research Documents and Data

Routine care research data and documents will be stored at the end of the research by the investigator and the manager for 15 years.

9. STATISTICS

9.1 Justification of sample size

The national survey conducted in 2010 [11] found an average BioSecure score in the non-TPE group of 68.09±18.28. Patients who reported they had had TPE had a score of 75.66±14.20. In our study, we expect a difference of 10 points or 15% increase in score. Indeed, patients in the national survey were already treated with bDMARDs whereas patients included in our study will be bDMARDs naïve, with inclusion taking place at the initiation of treatment. The expected scores in the control group will therefore be lower. Under the hypothesis of a 15% relative increase in score with TPE, with alpha=5%, beta=20% and 25% non-assessable patients, 120 subjects should be included .

9.2 Description of the planned statistical methods, including the schedule of planned interim analyses

No intermediate analysis will be planned.
The analysis will be performed after the database freezing and blinded to the randomization group.
The analysis will be conducted in intention to treat population. The primary outcome will then be analyzed in per protocol population.
The analysis will be performed with SAS V9.3 software.

Description of the baseline characteristics

The patients baseline characteristics will be described in total and by randomization group. Qualitative data will be described by frequencies and numbers, and quantitative variables will be described by their means and standard deviations, ranges or medians and interquartile ranges.

Analysis of the primary outcome:
The primary outcome will be the patient's BioSecure score [7] at 6 months. The BioSafe questionnaire consists of 24 competencies assessed in two parts, one part common to patients with intravenous (IV) and subcutaneous (SC) bDMARDs and the second part only for patients with SC bDMARD.
Part 1, includes 8 questions and 6 case scenarios.
Part 2 includes 1 question and 1 additional case.
Each correct answer is associated with 1 point.
The competency score is calculated as the sum of the points obtained, reported to 100.

The mean value of the BioSecure score at 6 months (with distinction between SC and IV patients) will be compared by a Student t-test or a non-parametric test if necessary.

Secondary analysis
Secondary outcomes

The criteria will be quality of life assessed by the SF12 questionnaire at BL and 6 months and adherence to biotherapy assessed by the MMAS 4 questionnaire at 6 months.

- The MMAS4 score is composed of 4 items with a yes or no response modality, yes being rated at 1. The score is calculated by the sum of the responses by items.
Adherence, a categorical variable with three classes, is then defined as follows:
MMAS 4 score = 0 : Very adherent patient
MMAS 4 score = 1 or 2 : Moderately adherent patient
MMAS 4 score = 3 or 4: Non-adherent patient
Adherence at M6 will be described globally and by group and will be compared between groups by a Pearson chi-square test or a Fisher exact test.

- The MOS-SF-12 questionnaire [15] is derived from the SF36 questionnaire.
It includes 12 items divided into eight dimensions (physical function, physical limitation, physical pain, emotional limitation, mental health, vitality, social functioning, overall perceived health).

The Mental Quality of Life (MQL) score and the Physical Quality of Life (PQL) score are then calculated only if all 12 items have been completed. Scores range from 0 to 100.
The scores at BL and 6 months will be described by groups.
Each score differences between BL and 6 months will be calculated and compared between groups, by a linear regression taking into account the BL score value.

Other Secondary Criteria

- Severe infections, defined as infections requiring hospitalization and/or intravenous antibiotics.

Treatment-related severe infections rate occurring within 6 months after initiating bDMARDS will be described and compared between groups using an accurate Fisher's test.

- Coping with the disease and psychological well-being will be assessed by a NRS. The obtained score will therefore be an integer between 0 and 10.

Scores will be described at BL and 6 months.
The difference between BL and 6 months will be calculated and compared between groups by a linear regression with account for the initial value. If difference distribution does not follow a normal distribution, the percentage of change will be calculated and compared between groups by a nonparametric Wilcoxon-Man Whitney test.

- Opinions about treatment, evaluated by the BMQ belief about medicine questionnaire

- The value of the BioSecure score will be described according to the type of bDMARD received (IV, SC).
- The value of the BioSecure score will be described according to: gender, age, education level, disease duration and underlying disease (Rheumatoid Arthritis or Axial Spondyloarthritis). The score values will be compared by a t-test or a non-parametric test for the qualitative variables. The correlation between scores and age will be assessed either by calculating a Pearson or by Spearman correlation coefficient if applicable. A coding of age into classes will be considered.

These last two analyses will be performed on the population per protocol.

9.3 Consideration of missing data

If the BioSecure score cannot be calculated (due to not collected questionnaires or questionnaire missing data), the patient will be considered a failure regardless of the randomization group. The score will be replaced by the 25 percentile value, calculated on the total population.

9.4 Populations for analysis

The ITT (Intention To Treat) population will consist of all randomized patients with no major protocol deviation. The per-protocol population will consist of randomized patients who have fully participated in the study, with no protocol deviation and whose BioSecure total score will allow calculations.

10. MANAGEMENT OF ADVERSE EVENTS

10.1 Adverse events (i.e., complications) related to medical procedures, combinations of procedures or prevention strategies, diagnosis or treatment that are part of the routine care (excluding medication).

In the context of the research aimed at evaluating routine care, medical procedures and strategies which are the subject of the research, will be part of usual care and delivered in accordance with their indications. Potential adverse events are therefore those related to the patient's usual care (care-related) and do not require specific reporting by the research manager.

10.1.1 Adverse events (i.e. complications) related to the care evaluated in the research.

10.1.2 Circuit for reporting adverse events (i.e. complications) related to care

These events will follow the usual reporting circuit provided by the current regulations.
For example:

- Adverse reactions that may be drug-related should be reported to the Regional Pharmacovigilance Centre.

10.2 Adverse events with special follow-up by the manager

Not applicable

10.3 Adverse events related to special monitoring added by research

Monitoring added by research to assess routine care involve negligible risks and constraints for the research subject. Therefore, no procedures for managing severe adverse events are imposed by the research.

11. PERSONS INFORMATION METHODS

In accordance with ***Law No. 2004-806 of August 9, 2004 on public health policy,*** the investigator has an ***obligation to inform individuals*** prior to their participation in research aimed at evaluating routine care.

11.1 Information of the person.

In accordance with article R. 1121-3 of the Code for Public Health, information for persons who entering into research is needed by delivering a written form, submitted in advance to the Ethical Committee (Committee for the Protection of the Persons CPP).
The patient will be informed (with a written form) by the rheumatologist in usual care. After a potential reflection time, the non-opposition form will be collected during the inclusion visit.

When research will be completed, the person participating in the research will be informed of the research overall results in the way specified in the information brochure.

12. ETHICAL AND LEGAL ASPECTS

12.1 Declaration that the research will be conducted in accordance with the protocol, current laws and regulations.

The manager and the person(s) directing and supervising the research will commit to ensure that this research is carried out in accordance with the law n°2004-806 of August 9, 2004 relating to public health

policy and the current regulations. (Articles L1121-1, 2° paragraph and R1121-3 of the Public Health Code).

Research recorded data will be subject to computerized processing in compliance with the modified law n°78-17 of January 6, 1978 relating to data processing, files and freedom.

The research will be conducted in accordance with this protocol.

12.2 Ethical evaluation of the specific monitoring methods provided for in the protocol

The specific monitoring methods added by the research have been examined for ethical evaluation by the Ethical Committee (Committee for the Protection of the Persons CPP) Ile de France VI. They entail only negligible risks and constraints for the persons involved in the research.

12.3 Legal requirements (Role of the manager, CPP, CCTIRS, CNIL)

12.3.1 Role of the manager

The Assistance Publique des Hôpitaux de Paris (AP-HP) will be the manager of this research and be represented by the Delegation for Research and Innovation (DRCI).

The so-called manager will take the initiative of this research, will ensure its management and will check that the funding is provided. The manager will submit the protocol for approbation to the Committee for the Protection of Persons (CPP).

In accordance with amended law n°78-17 of January 6, 1978, the manager will send an opinion request to the Consultative Committee on data collection in Health Research (Comité consultatif sur le traitement de l'information en matière de recherche dans le domaine de la Santé (CCTIRS) and an authorization request to the National Data Protection Commission (Commission Nationale pour Informatique et Liberté (CNIL)) for the data processing during the research.

12.3.2 Submission to the CPP

This research obtained the favorable decision of the Comité de Protection des Personnes CPP Ile de France IV (Pitié-salpétrière) on 22/06/2016.

The opinion of the above-mentioned committee was notified in the information form to the persons concerned.

12.3.3 Notice from CCTIRS and authorization from the CNIL

This research is subject to the modified law n°78-17 of January 6, 1978 relating to data collection, files and freedom.

Consequently, the data collected in the context of multi-center research needs to be referred for advice to the Consultative Committee on data collection in Health Research (CCTIRS) and for authorization to the National Data Protection commission (CNIL).

For monocentric research, only a regular declaration to the CNIL is necessary.

Information on the persons rights who will participate in the research is included in the information form for the patient: right of access and rectification, right to oppose the transmission of data covered by professional secrecy that may be used in the context of this research.

12.3.4 Substantial modification to the protocol

The investigator or coordinator will inform the Delegation for Clinical research and Innovation (DRCI) of any intended changes to the protocol. Any substantial change will be submitted for advice to the CPP by the research manager.

12.3.5 Final research report or publication

The final research report or publication will be submitted for comments to every participating centers. The final version will be sent to the manager as soon as possible after the effective research end.

12.3.6 Data ownership

AP-HP is the data owner and no use or transmission to a third party will be authorized without prior consent.

12.3.7 Publication rules

You must mention the AP-HP in the affiliations of the author(s) of the publications resulting from your research and mention the AP-HP Manager (DRCI).

1. ***Mention of AP-HP affiliation for projects managed by PA-HP***
   - *If an author has several affiliations, the order in which the institutions (AP-HP, University, INSERM...) are cited is not important.*
   - *Each of these affiliations must be identified by an address separated by a semicolon (;).*
   - *The AP-HP institution must appear under the abbreviation "**AP-HP**" first in the address followed precisely by **AP-HP**, hospital, department, city, postal code, France*
2. ***Mention of the AP-HP manager (DRCI)** in **the acknowledgments of the** manuscript*
   - *"The sponsor was Assistance Publique - Hôpitaux de Paris (Department of Clinical Research and Development)".*

This search is registered on the http://clinicaltrials.gov/ website under the n°NCT02855320

13. BIBLIOGRAPHICAL REFERENCES

1. Saraux A, Combe B, Blin P et al, Survey of the therapeutic management of rheumatoid arthritis in France: the OPALE study. Clin Exp Rheumatol, 2010, 28(3):325-32. Epub 2010 Jun 23

2. Singh JA, Cameron C, Noorbaloochi S et al, Risk of serious infection in biological treatment of patients with rheumatoid arthritis: a systematic review and meta-analysis. Lancet, 2015 May 11. pii: S0140-6736(14)61704-9. doi: 10.1016/S0140-6736(14)61704-9. [Epub ahead of print]

3. Dixon WG, Symmons DP, Lunt M et al Serious infection following anti-tumor necrosis factor alpha therapy in patients with rheumatoid arthritis: lessons from interpreting data from observational studies. Arthritis Rheum. 2007 Sep;56(9):2896-904.

4. http//www.has.fr. Structuring a therapeutic patient education (TPE) program in the field of chronic diseases [Therapeutic patient education (TPE) Definition, goals, and organization]. Recommendations Haute Autorité de Santé 2007.

5. Gaujoux-Viala C, Gossec L, Cantagrel A et al, Recommandations of 81(4):287-97.the French Society of Rheumatology for managing rheumatoid arthritis. Joint Bone spine, 2014, 81(4):287-97.

6. Wendling D, Lukas C, Paccou et al, Recommendations of the French Society for Rheumatology (SFR) on the everyday management of patients with spondyloarthritis. Joint Bone Spine. 2014 Jan;81(1):6-14. doi: 10.1016/j.jbspin.2013.12.002. Epub 2014 Jan 10 http://www.ncbi.nlm.nih.gov/pubmed/24412120

7. Gossec L, Fautrel B, Flipon É, Lecoq d'André F, Marguerie L, Nataf H, Pallot Prades B, Piperno M, Poilverd RM, Rat AC, Sadji F, Sordet C, Thevenot C, Beauvais C. Safety of biologics: elaboration

and validation of a questionnaire assessing patients' self-care safety skills: the BioSecure questionnaire. An initiative of the French Rheumatology Society Therapeutic Education section. Joint Bone Spine. 2013 Oct;80(5):471-6. doi: 10.1016/j.jbspin.2012.11.009. Epub 2013 Aug 20.

8. L. Pfeffer, A. Janoly-Dumenil, A. Donjon. Effects of an educational program on the safety knowledge and skills of patients with rheumatoid arthritis treated by biologics. Ann Rheum Dis 2013;72(Suppls3):150

9. Berard L , J.-F. Menard JF , J. Benichou J , T. Lequerre T , Vittecoq O , S. Pouplin S Effectiveness of collective educational intervention in the management of sub cutaneous biological agents in patients with inflammatory rheumatism. Ann Rheum Dis 2013;72(Suppls3):355

10. Orefice D, Beauvais C, Gossec L et al Cross-sectional study of self-care safety skills in 677 patients on biodrugs for inflammatory joint disease. Joint Bone Spine. 2014 Dec;81(6):502-7. doi: 10.1016/j.jbspin.2014.04.010. Epub 2014 Jun 23.

11. Rat AC, Fautrel B, Flipon E, Gossec L, Marguerie L, Nataf H, Pallot Prades B, Poilvert RM, Royant V, Sadji F, Sordet C, Thevenot C, Beauvais C. "Factors associated with knowledge and safety skills of arthritis patients receiving biologics: a survey of 677 patients". Soumis à publication à Rheumatology.

12. Beauvais C, Perdriger A. Diversity and territorial distribution of therapeutic education programs for osteoarticular diseases: a census of regional health agencies. Rev Rhum, 2012, 79, A301

14. Morisky DE, Green LW, Levine DM. Concurrent and predictive validity of a self-reported measure of medication adherence. Med Care. 1986 Jan;24(1):67-74

15. J N Burdine, M R Felix, A L Abel et al The SF-12 as a population health measure: an exploratory examination of potential for application. Health Serv Res. 2000 Oct; 35(4): 885-904.

16. Gossec L, Dougados M, Rincheval N et al. Elaboration of the preliminary Rheumatoid Arthritis Impact of Disease (RAID) score: a EULAR initiative. Ann Rheum Dis. 2009 Nov;68(11):1680-5. doi: 10.1136/ard.2008.100271. Epub 2008 Dec 3.

17. Nicassio PM, Wallston KA, Callahan LF, Herbert M, Pincus T: The measurement of helplessness in rheumatoid arthritis. The development of the arthritis helplessness index. J Rheumatol 1985, 12:462-467

18. L. Prevoo, M.A. van't Hof, H.H. Kuper, et al.Modified disease activity scores that include twenty-eight-joint counts. Development and validation in a prospective longitudinal study of patients with rheumatoid arthritis.Arthritis Rheum, 38 (1995), pp. 44-48

19. Fransen J, van Riel PL. The Disease Activity Score and the EULAR response criteria. Clin Exp Rheumatol. 2005 t;23(5 Suppl 39):S93-9

20. Machado P, Landewé R, Lie E et al Ankylosing Spondylitis Disease Activity Score (ASDAS): defining cut-off values for disease activity states and improvement scores. Assessment of SpondyloArthritis international Society. Ann Rheum Dis. 2011 Jan;70(1):47-53. doi: 10.1136/ard.2010.138594. Epub 2010 Nov 10

21. Garrett S, Jenkinson T, Kennedy LG et al A new approach to defining disease status in ankylosing spondylitis: the Bath Ankylosing Spondylitis Disease Activity Index. J Rheumatol. 1994 Dec;21(12):2286-91.

22. Lukas C, Landewé R, Sieper J et al Development of an ASAS-endorsed disease activity score (ASDAS) in patients with ankylosing spondylitis. Ann Rheum Dis. 2009 Jan;68:18-24. doi: 10.1136/ard.2008.094870. Epub 2008 Jul 14.

23. Aletaha D, Neogi T, Silman AJ et al, 2010 rheumatoid arthritis classification criteria: an American College of Rheumatology/European League Against Rheumatism collaborative initiative. Arthritis Rheum. 2010;62:2569–2581

24. Rudwaleit M, van der Heijde D, Landewé R et al, The development of Assessment of SpondyloArthritis international Society classification criteria for axial spondyloarthritis (part II): validation and final selection. Ann Rheum Dis. 2009 Jun;68(6):777-83. doi: 10.1136/ard.2009.108233. Epub 2009 Mar 17

25. Rudwaleit M1, van der Heijde D, Landewé R et al The Assessment of SpondyloArthritis International Society classification criteria for peripheral spondyloarthritis and for spondyloarthritis in general. Ann Rheum Dis. 2011 Jan;70(1):25-31. doi: 10.1136/ard.2010.133645. Epub 2010 Nov 24.

26. Neame R, Hammond A. Beliefs about medications: a questionnaire survey of people with rheumatoid arthritis. Rheumatology 2005 ;44(6):762-7.

14. APPENDICES

14.1 Appendix 1: List of participating centers in June 12, 2017

N°	DEPARTMENT	FULL NAME AND ADDRESS	INVESTIGATORS	CONTACT DETAILS
**001**	Rheumatology	CHU Saint Antoine, 184, rue Faubourg Saint Antoine, 75012 Paris, France	Catherine Beauvais	Tel: 01 49 28 25 20<br>Email: catherine.beauvais@aphp.fr
**002**	Rheumatology	CHU Pitié Salpétrière, 47-83 Boulevard de l'Hôpital, 75013 Paris.	Laure Gossec	Tel: 01 42 16 00 00<br>Email: laure.gossec@aphp.fr
**003**	Rheumatology	CHU Clermont Ferrand Hospital Gabriel Montpied 58 rue Montalembert. 63003 Clermont-Ferrand.	Martin Soubrier (IP)<br>Françoise Fayet	Tel: 04 73 75 07 50<br>Email: ideetp@chu-clermontferrand.fr<br>msoubrier@chu-clermontferrand.fr
**004**	Rheumatology	CHU Grenoble Hospital South, Avenue de Kimberley<br>CS 90338 - 38434 CHIPS	Laurent Grange	Tel: 04 76 76 75 75<br>Email: LGrange@chu-grenoble.fr
**005**	Rheumatology	CHU Brabois, rue du Morvan 54511 VANDOEUVRE LES NANCY.	Anne Christine Rat	Tel: 03 83 85 11 88<br>Email: ac.rat@chu-nancy.fr
**006**	Rheumatology	CHU Rouen, 1 rue de Germont 76031 Rouen, France	Sophie Pouplin	Tel: 02 32 88 89 90<br>Email: Sophie.Pouplin@chu-rouen.fr
**007**	Rheumatology	CHU Nantes Hôtel Dieu, Place Alexis Ricordeau 44093 Nantes cedex 1	Yves Maugars (IP)	Tel: 02.40.08.33.33<br>Email: yves.maugars@chu-nantes.fr
**008**	Rheumatology	University Hospitals Strasbourg, 1 Av	Christelle Sordet	Tel: 03 88 11 67 68

		Moliere 67098 Strasbourg Cedex, France		Email: Christelle.SORDET@chru-strasbourg.fr
**009**	Rheumatology	CHRU Pontchaillou, 2 rue Henri Le Guilloux 35033 RENNES	Aleth Perdriger	Tel : 02 99 28 43 21 Email: aleth.perdriger@chu-rennes.fr
**010**	Rheumatology	CHU Saint Etienne Hospital of Bellevue	Béatrice Pallot Prades	Email: beatrice.pallotprades@chu-st-etienne.fr

14.2 Appendix 2: Questionnaires

14.2.1 BioSecure Questionnaire

This questionnaire contains important information about your knowledge of the biologic treatment you take for your arthritis. Please answer the following questions, even if you feel they do not concern you. For each question, please tick the answer which best corresponds to what you think or feel Thank you

Your name: ……………………………………………………………………

Today's date: …………………………………………….

<u>Questions.</u>

1. What is your current biologic treatment? *Please tick one answer only*

□ Enbrel (etanercept)
□ Humira (adalimumab)
□ Remicade (infliximab)
□ Mabthera (rituximab)
□ Orencia (abatacept)
□ RoActemra or Actemra (tocilizumab)
□ Cimzia (certolizumab)
□ Other ……………………………
□ I don't know

2. I can stop my biologic treatment if my arthritis is completely under control (in remission). *Please tick one answer only.*

Yes □ No □ I don't know □

3. Infections are more common during biologic treatment. *Please tick one answer only.*

Yes □ No □ I don't know □

4. Among the following situations, which ones require special precautions or a change of your biologic treatment? *Please tick one answer for each situation.*

	Yes	No	I don't know
4.1. Drinking milk	□	□	□
4.2. Foreign travel	□	□	□
4.3. Having an operation	□	□	□
4.4. Running	□	□	□
4.5. Having a tooth extraction	□	□	□
4.6. Drinking a glass of wine	□	□	□
4.7. Eating organic food	□	□	□
4.8. Planning having a baby	□	□	□

5. Who do I need to tell about my biologic treatment? *Please tick one answer for each person.*

	Yes	No	I don't know
5.1. My doctor (general practitioner)	□	□	□
5.2. My employer	□	□	□
5.3. My dentist	□	□	□
5.4. The anesthetist in case of surgery	□	□	□

5.5. My fitness instructor or sports coach	□	□	□
5.6. The lifeguard at the swimming pool	□	□	□
5.7. My bank manager.	□	□	□

6. When taking a biologic, all vaccinations should be avoided. *Please tick one answer only.*

true □ *false*□ *I don't know*□

7. When using biologics, a woman must use effective contraception. *Please tick one answer only.*

true □ *false*□ *I don't know*□

8. Which of the following situations lead to special precautions or to modifications in the management of biologic treatment? *Please tick the right answer for each situation.*

	Yes	No	I don't know
8.1. High temperature / fever	□	□	□
8.2. Frequent need of urinating	□	□	□
8.3. A sprained ankle	□	□	□
8.4. A cough	□	□	□
8.5. Out of breath for no apparent reason	□	□	□
8.6. Constipation	□	□	□
8.7. Have a burning sensation while urinating	□	□	□
8.8. A weight gain of 3 kilograms (6 pounds)	□	□	□
8.9. A weight loss of 1 kilogram (2 pounds)	□	□	□

Clinical situations

Biologic treatment may be given by sub-cutaneous injections at home or infusions at hospital. The following situations refer to both administration type. Please answer the questions by referring to your own situation.

Case no.1

Cathy is receiving biologic treatment for rheumatoid arthritis. During the Christmas holidays, her husband and daughter have fever with a temperature of 38°5 C (101 degrees Fahrenheit), they have a cough and a runny nose. Their doctor gave them some treatment but said they did not need antibiotics since it was only a viral infection. One week later, Cathy develops the same symptoms: fever, a cough and a runny nose. Which of Cathy's decisions (below) do you agree with? *(several possibilities)*

		Yes	No	I don't know
1	Cathy takes the treatment which was prescribed for her husband, since it was effective for him.	□	□	□
2	She waits a few days before contacting the doctor because her husband and daughter recovered in a few days.	□	□	□

3	She has her biologic treatment (injection or infusion) because it's only a virus.	□	□	□

Case no. 2

Paul's arthritis is treated with biologics. The day before his biologic treatment (injection or infusion) he has bronchitis with a barking cough and a temperature of 38°5 C (101 degrees Farhenheit). His doctor prescribes antibiotics which Paul starts the same evening. Paul decides he should not have his biologic treatment (not perform the injection or tell the infusion centre not to perform the infusion). Which of the following statements do you agree with? *(several possibilities)*

		True	False	I don't know
1	Paul was right not to take his biologic treatment.			
2	Paul was right to start antibiotics as soon as possible.			
3	If Paul has bronchitis again, he will know which antibiotics he can take in case his doctor is not available.			
4	Paul can have his biologic treatment tomorrow if he starts the antibiotics today.			
5	Paul was right to call his doctor.			

Case no. 3

This summer, Christine went on holiday with her family. During her stay, she rested a lot and her arthritis was much better. She only needed anti-inflammatory drugs for a few days and she decided not to carry on with her biologic therapy (injection or infusion). When she came back, one of her friends asks her "Why should you start your biologic again, as you no longer have pain?"

Which of Christine's answers (below) do you agree with? *(several possibilities)*

		Yes	No	I don't know
1	I will start my biologic again.	□	□	□
2	If my arthritis has not been painful for 3 weeks, it probably means that it is cured.	□	□	□

Case no. 4

In October, Jane, aged 53, learns that the flu vaccine is now available. She has been treated by biologics for 6 months. She is not sure whether or not she should have the flu vaccine. With which of Jane's opinions (below) do you agree with? *(several possibilities)*

		Yes	No	I don't know
1	I will get the flu vaccine.	□	□	□
2	I am more likely to have a reaction to the flu jab due to my biologic treatment.	□	□	□
3	I have to avoid the flu jab because of my biologic treatment.	□	□	□

4	I will talk about it with my doctor.	□	□	□

Case no. 5

Bill is treated by biologics for arthritis; he likes gardening. Bill has cut his index finger while planting a rosebush. Which of the following statements (below) do you agree with? *(several possibilities)*

		Yes	No	I don't know
1	The wound needs to be cleansed and dressed straight away.	□	□	□
2	The wound is more likely to go septic because of the biologic therapy.	□	□	□
3	Bill must take antibiotics straight away.	□	□	□
4	Bill can have the tetanus vaccine, even though he is treated with a biologic.	□	□	□

Case no.6

Sarah is treated with biologics. She has an appointment with a surgeon to plan cataract surgery. The surgeon proposes to operate in 10 days.
Which of the following statements (below) do you agree with? *(several possibilities)*

		Yes	No	I don't know
1	The surgery should definitely be avoided.	□	□	□
2	Sarah agrees with the scheduled date for the operation, the sooner the better.	□	□	□
3	Sarah refuses the scheduled date because she needs to think about stopping her biologic therapy first.	□	□	□
4	Sarah informs the surgeon about her biologic therapy.	□	□	□
5	Sarah informs the anesthetist about her biologic therapy.	□	□	□

If you have subcutaneous biologic therapy, please answer the following questions:

Question 9

Please tick one answer only.
The biologic treatment must be stored:
- in the refrigerator □

- in the freezer □
- at room temperature □
- I don't know □

Case no. 7

Alice is about to do her biologics sub cutaneous injection. Which of Alice's actions was incorrect? *Tick one only.*

- □ She gets the biologic out and waits a little before injection
- □ She disinfects the skin
- □ She injects herself in the abdomen or the thigh
- □ She puts the used syringe and needle in the wastebin.

2. In your opinion, is there anything Alice has forgotten to do? *(Only one sentence)*

Thank you for filling in this questionnaire.

14.2.2 SF 12

The following questions ask for your views about your health. Your answers will help monitor the condition of your health and to know how well you are able to do your usual activities. Answer all of the following questions by following the instructions you have been given. If you are unsure, please give the best answer you can.

1.In general, would you say your health is:
(mark one answer only)

- Excellent ....❑
- Very good...❑
- Good...❑
- Fair ....❑
- Poor ....❑

2.The following is a list of activities you might do during a typical day. For each of these, indicate whether your health now limits you in these activities. (mark one answer only per line).

	Limited a lot	Limited a little	Not limited at all
A.Moderate physical activities such as moving a table, pushing a vacuum cleaner, bowling	❑	❑	❑
B.Climbing several flights of stairs	❑	❑	❑

3.During the past 4 weeks, due to your physical condition, (mark one answer only per line)

	All the time	Most of the time	Some of the time	A little of the time	None of the time
A.Have you accomplished less than you would like?	❑	❑	❑	❑	❑
B.Have you had to stop doing certain things?	❑	❑	❑	❑	❑

4.During the past 4 weeks, due to your emotional state (such as feeling sad, nervous or depressed) (mark one answer only per line)

	All the time	Most of the time	Some of the time	A little of the time	None of the time
A.Have you accomplished less than you would like?	❑	❑	❑	❑	❑
B.have you had difficulties in doing what you had to do with as much care and attention as usual?	❑	❑	❑	❑	❑

5.During the past 4 weeks, how much did your physical pain interfere with your work or housework? (mark one answer only)

- Not at all ....❑
- A little bit..❑
- Moderately ....❑
- Quite a bit ....❑
- Extremely ....❑

6.The following questions are related to how you have felt over the past 4 weeks. For each question, please indicate the response you feel is most appropriate.

During the past 4 weeks, have there been times when: (mark one answer only per line)

	All the time	Most of the time	Some of the time	A little of the time	None of the time
A.Have you felt calm & peaceful?	❑	❑	❑	❑	❑
B.Have you felt full of energy?	❑	❑	❑	❑	❑
C.Have you felt down-hearted and blue?	❑	❑	❑	❑	❑

7.During the past 4 weeks, how much of the time has your physical health or emotional problems interfered with your social activities and relationships with others (family, friends, etc.)? (mark one answer only)

- All the time ....❑
- Most of the time ....❑
- From time to time ....❑
- Rarely ....❑
-Never ……………………………………….. ❑

14.2.3 MMAS Morisky modified adherence scale

Please check one box on each line:

1.Do you ever forget to take your biologic treatment ?	❑ yes	❑ no
2.Do you ever have problems remembering to take your biologic treatment	❑ yes	❑ no
3.When you feel better, do you sometimes stop taking your biologic treatment ?	❑ yes	❑ no
4.Sometimes if you feel worse when you take your biologic treatment (name of health condition) medicine, do you stop taking it?	❑ yes	❑ no

BMQ

I would like to ask you about your personal views about medicines prescribed for your arthritis. These are statements other people have made about their arthritis medication. Please indicate the extent to which you agree or disagree with them by placing a cross in the appropriate box. Please only cross one box per question.

	Strongly agree	Agree	Uncertain	Disagree	Strongly disagree
My health, at present, depends on my medicines					
My life would be impossible without my medicines					
Without my medicines I would become very ill					
My health in the future will depend on my medicines					
My medicines protect me from becoming worse					
Having to take medicines worries me					
I sometimes worry about the long-term effects of my medicines					
My medicines are a mystery to me					
My medicines disrupt my life					
I sometimes worry about becoming too dependent on my medicines					

14.2.4 RAID: Rheumatoid Arthritis Impact of Disease

1. Pain

Circle the number that best describes the pain you felt due to your rheumatoid arthritis during the last week:

None 0 1 2 3 4 5 6 7 8 9 10 Extreme

2. Functional disability assessment

Circle the number that best describes the difficulty you had in doing daily physical activities due to your rheumatoid arthritis during the last week.

No difficulty	0	1	2	3	4	5	6	7	8	9	10	Extreme difficulty

3. Fatigue

Circle the number that best describes how much fatigue you felt due to your rheumatoid arthritis during the last week.

No fatigue	0	1	2	3	4	5	6	7	8	9	10	Totally exhausted

4. Sleep

Circle the number that best describes the sleep difficulties (i.e., resting at night) you felt due to your rheumatoid arthritis during the last week.

No difficulty	0	1	2	3	4	5	6	7	8	9	10	Extreme difficulty

5. Physical well-being

Considering your arthritis overall, how would you rate your level of physical well-being during the past week? Circle the number that best describes your level of physical well-being.

Very good	0	1	2	3	4	5	6	7	8	9	10	Very bad

6. Emotional well-being

Considering your arthritis overall, how would you rate your level of emotional well-being during the past week? Circle the number that best describes your level of emotional well-being.

Very good	0	1	2	3	4	5	6	7	8	9	10	Very bad

7. Coping

Considering your arthritis overall, how well did you cope (manage, deal, make do) with your disease during the last week?

Very well	0	1	2	3	4	5	6	7	8	9	10	Very poorly

14.2.5 Arthritis helplessness index

	Strongly disagree	Disagree	Agree	Strongly agree
My condition is controlling my life	❑	❑	❑	❑
No matter what I do, or no matter hard I try, I just can't seem to get relief from my pain.	❑	❑	❑	❑
I am coping effectively with my condition	❑	❑	❑	❑
I would feel helpless if I couldn't rely on other people for help with my condition	❑	❑	❑	❑
It seems as though fate and other factors beyond my control affect my condition	❑	❑	❑	❑

14.2.6 Disease activity score DAS 28

Number of tender joints NAD (0-28)
Number of swollen joints NAG (0-28)
Patient global assessment 0-10 (question1)
Erythrocyte sedimentation rate (mm)
C reactive protein) mg/ml

Question1 : Considering all the ways your arthritis has affected you, how active do you feel your arthritis is … ?

14.2.7 BASDAI Bath Ankylosing Spondylitis Disease Activity Index

Please place a mark on each line below to indicate your answer to each question relating to the past week

1.How would you describe the overall level of fatigue/tiredness you have experienced?

None 0 1 2 3 4 5 6 7 8 9 10 Very severe

2.How would you describe the overall level of AS neck, back or hip pain you have had?

None 0 1 2 3 4 5 6 7 8 9 10 Very severe

3.How would you describe the overall level of pain/swelling in joints other than neck, back or hips you have had?”

None 0 1 2 3 4 5 6 7 8 9 10 Very severe

4.How would you describe the overall level of discomfort you have had from any areas tender to touch or pressure?

None 0 1 2 3 4 5 6 7 8 9 10 Very severe

5.How would you describe the overall level of morning stiffness you have had from the time you wake up?

None 0 1 2 3 4 5 6 7 8 9 10 Very severe

6.How long does your morning stiffness last from the time you wake up?

0 hrs----------------------½-----------------------1---------------------½-----------------2 or more

14.2.8 ASDAS

Please place a mark on each line below to indicate your answer to each question relating to the past week
1.How would you describe the overall level of AS neck, back or hip pain you have had?

None	0	1	2	3	4	5	6	7	8	9	10	Very severe

2.How long does your morning stiffness last from the time you wake up?

0 hrs---------------------½----------------------1--------------------½----------------2or more

3.How would you describe the overall level of pain/swelling in joints other than neck, back or hips you have had?"

None	0	1	2	3	4	5	6	7	8	9	10	Very severe

4. How active was your spondylitis on average during the last week?

Not at all	0	1	2	3	4	5	6	7	8	9	10	Very much

14.3 Appendix 3: Nurse Education Booklet

BIOSAFE PROTOCOL
EDUCATION BOOKLET

This booklet includes

A. At Baseline

1) A patient self-administered questionnaire (document 1) to be completed prior to the nurse intervention. This patient self-administered questionnaire will remain in the investigating center and is complementary to the patient study questionnaire that the nurse will have imperatively consulted before the interview.
2) A semi-structured interview schedule (document 2). This schedule will explore the patient's expectations, concerns and motivation for treatment. It will remain in the investigating center. Since all nurses will be trained in TPE, it will be left to the discretion of each nurse whether or not to use this guide.
3) In total, the interview should have addressed a number of domains listed in the interview guide. To check the intervention quality , the nurse will fill in the following form: the interview checklist
4) Interview checklist (document 3) attesting that the interview domains have been addressed. This checklist will only be filled-in by nurses who will not have used the interview schedule. This checklist will remain in the center. Then the nurse will fill in;
5) Intervention self-assessment (document 4) on the quality of the intervention or possible difficulties which will be sent to the Unit for Clinical Research (UCR). This self-assessment is not a value judgment but a record of the intervention’s feasibility.
6) Safety checklist (document 5) will include important safety messages to be taught to the patient. This checklist will remain in the investigating center.
7) Self-assessment by the patient of the skills he/she has acquired that will remain in the investigating center (document 6).
8) The total duration of the intervention will be collected.
9) Skills evaluation (document 7) will be send to the UCR.
10) Interview free summary will remain in the investigating center

At the end of the intervention the BioSecure brochure will be provided

In total, all personal patient data collected in this booklet will be used for patient education and will not be collected for the study.

For intervention quality checking, only the self-assessments documents 4 and 7 will be sent to the UCR as well as the total intervention duration.

B. A 3 months

11) Semi-structured interview for events and patient’s feedback since BL(document 8).

The nurse will inquire about events since BL, possible patients’ difficulties, and their degree of motivation. Since all nurses will be trained in therapeutic education, it will be left to the discretion of each nurse whether to use this guide or not. In total, the interview should have addressed a certain number of themes listed in the interview guide. To check the intervention quality, the nurse will fill in the following form: Interview checklist

12) Interview checklist (document 9) attesting that the domains have been addressed. This checklist will only be completed by nurses who did not use the interview guide. This checklist will remain in the investigating center.
13) Intervention self-assessment (document 10) for on the intervention quality or potential difficulties that will be sent to the UCR. This self-assessment is not a value judgment but a record of the intervention’s feasibility.
14) Safety checklist (document 5) i.e the same as at BL with repeated messages. In this checklist, the nurse will ask open-ended questions to see if the patient has acquired the knowledge and skills needed for bDMARD.

15) Skills evaluation (document 7) i.e the same as at BL on the intervention quality or possible difficulties which will be sent to the UCR.
16) The total duration of the intervention will be collated.
17) Free summary of the interview
18) Reporting to the physician of situations requiring his/her intervention

Baseline

1) Document 1 : patient self-administered questionnaire

(will remain in the center)

This questionnaire should be provided prior to the interview in order to explore the patient's educational background.

Nurse will have access to the self-completed questionnaire for other aspects of patient characteristics.

Dear Sir/Madam, you are going to meet a nurse to learn how to manage your new treatment.

To know more about you and your personal needs, please fill out the questionnaire below.

FILLING DATE I__I__I I__I__I I_2_I_0_I__I__I

Dear Sir/Madam,
you are going to meet a nurse to learn how to manage your new treatment.
To know more about you and your personal needs, please fill out the questionnaire below.

Your perception of your disease

How did you feel when you were diagnosed?
How do you perceive the progression of your disease, its severity?
Do you think that certain factors may have triggered the progression of your disease?
□ Yes □ No
If so, which ones?
Your comments:

Your perception of your treatments

Do you know the side effects of your current medications? □ Yes □ No
Do you know what to do if one of these effects happens? □ Yes □ No
Do you have problems understanding and taking your current treatment? □ Yes □ No
Do you have any concerns about your treatment in general? □ Yes □ No
Do you have any concerns about the biologic you have been prescribed? □ Yes □ No
If so, which ones?
Did you experience non-pharmacological treatment or alternate medicines ?

- Physiotherapy □ Yes □ No
- Psychological support □ Yes □ No
- Homeopathy □ Yes □ No
- Acupuncture □ Yes □ No
- Diet for your arthritis □ Yes □ No
- Relaxation □ Yes □ No
- Other □ Yes □ No
- If so, which one?

Your comments:

The impact of your disease on your family life, social life and professional activities

Do you find help with family or friends? □ Yes □ No
Are you currently doing physical activity, sports or leisure activities? □ Yes □ No
Do you meet difficulties in your family and social life? □ Yes □ No

Do you meet difficulties in professional activity? □ Yes □ No
Do you find help with colleagues or your employer? □ Yes □ No

Your care by the health services system

Do you feel that your difficulties are being considered by health professionals? □ Yes □ No
Are you a member of a patient association? □ Yes □ No
Your comments:

Your attitudes towards your disease and its impact on your mood
What are your feelings about your disease?

□ Fear □ Lassitude
□ Anger □ Depression
□ Sense of injustice □ Indifference
□ Anxiety □ Serenity
□ Distress □ Other
□ Revolt

Did you find personal resources?

□ Energy
□ Fighting spirit
□ be able to put things into perspective
□ be able make choices, priorities
□ be able to react
Other

Do you have plans for your life in the near or medium term? □ Yes □ No
If so, which ones?
Your comments:

Thank you for completing this questionnaire

2) Document 2 : BL semi-structured interview schedule (will remain in the center)

4a. Semi-directive schedule with open questioning.

The nurse will refer to the answers of the self-administered questionnaire to feed the interview content.
Use open questions. Use active listening and reflective techniques. Always encourage the patient.

Have you ever heard of biologics?

What do you expect from this treatment?

Do you have any concerns about the biologic that has been prescribed for you?

Do you think it will be easy for you to take your medication?

Do you think you will be able to make the injection yourself?

You are about to start your treatment: in case of problems, do you know who to turn to? Provide the telephone number and email address of the rheumatology department

3) **<u>Document 3 Interview checklist</u> (will remain in the center) filled out only for nurses who did not use the interview guide**

The following points have been discussed:

Patients' perceptions and opinions of biologics □ Yes □ No
Patients' expectations of biologics □ Yes □ No
Concerns about biologics □ Yes □ No
Degree of motivation for biologics □ Yes □ No

Degree of motivation for self-injection of biologics □ Yes □ No

Anticipating possible problems and what to do in this case
- In case of problems who to turn to □ Yes □ No
- Proposal to contact the rheumatology nurse □ Yes □ No

At the end of the interview, all the boxes must be ticked, unless there is a particular difficulty (see self-assessment).

4) **<u>Document 4 intervention self-assessment (</u>will be sent to the CRU)**

Type of interview carried out by the nurse : free❑semi-structured❑

The semi-structured interview was carried out completely yes❑no❑

If not, it was difficult to address the following themes (multiple responses possible)
❑ Beliefs and opinions
❑ Expectations
❑ Concerns
❑ Motivation for bDMARDS
❑ Motivation for self-injection
❑ Anticipation of potential difficulties

5) <u>Document 5 Safety checklist **will**</u> **remain in the center**

The following educational elements should have been covered in the interview:

Skills to discuss with the patient	Done
Know the name of bDMARD	❑
Be aware that bDMARDs **should not be stopped or modified without the rheumatologist's agreement, except for safety reasons.**<br>*Message: The objevtive of bDMARDs is to stabilize the inflammatory disease and in the case of peripheral arthritis, also to prevent joints from being damaged. In RA, bDMARDs are most often combined with methotrexate to increase the treatment efficacy.*	❑
Know that bDMARDs **are associated with an increased risk of infection.**<br>*Message: Infections are globally doubled in frequency. The most frequent infections are upper respiratory tract infections, bronchitis, pneumonia and, in women, urinary tract infections. This is why it is important (1) to frequently wash your hands (2) to be up to date for vaccinations, especially pneumococcal and influenza (3) for women to drink enough water*	❑
Know that bDMARDs **should not be administered if you have a fever.**<br>*Message: in case of fever above 38°C and particularly above 38°5C it is imperative not to inject.*	❑
**Know when to call the doctor if you have a fever.**<br>*Message: in case of fever >38°C, you should see a doctor. In case of nasopharyngitis, without fever, delay the injection for 24-48 hours and in case of fever see the doctor.*	❑
**Know that you should not self-medicate with antibiotics.**<br>*Message: There are different types of antibiotics depending on the type of infection, so don't take an antibiotic that was effective on a previous infection because it may be ineffective on the current infection.*<br>*It is necessary to wait until the infection has been treated before starting the bDMARD again: generally you should wait 48 hours after stopping the antibiotics in agreement with the doctor.*	❑
Know that **fever should make you call a doctor**<br>*Message: in case of fever >38°C, you should see a doctor.*	❑
**Be aware that a frequent need to urinate or burning while urinating should lead to see a doctor**<br>*Message: these are symptoms suggestive of a urinary tract infection.*	❑
**Know that coughing should lead to consult a doctor**<br>*Message: This is a symptom suggestive of a lung infection or bronchitis, especially in case of wet or unusual cough.*	❑
**Know that shortness of breath should lead you to seek medical advice.**<br>*Message: this is a symptom suggestive of an infection or complication, such as a lung infection or a heart disease. In general, unusual symptoms should lead to a consultation, such as a significant loss of appetite, or a weight loss of several kg (a loss of 1 kg is not important).*	❑
**Know that all wounds must be disinfected:**<br>*Message: when you are treated with a bDMARD, a wound is more likely to get infected. It is recommended to delay the injection for 24-48 hours in case of a large wound, and to disinfect the wound with a standard disinfectant. It is not needed to take antibiotics if the wound is not infected. Do not self-medicate with antibiotics. The tetanus vaccine is usually checked before starting a bDMARD. Tetanus vaccine is administered every 10 years and is permitted when taking bDMARDs.*	❑
**Know that certain vaccines are contraindicated:**<br>*Message: live vaccines are contraindicated: these are yellow fever, chickenpox, measles-rubella-mumps, Koch's tuberculosis vaccine and oral poliomyelitis. When bDMARDs have already been started, talk to your doctor if you need to get these vaccines because certain bDMARDs will need to be stopped before vaccination.*<br>*Inactivated/killed/ vaccines are permitted. Updating vaccines helps prevent infections.*<br>*Influenza vaccine is recommended annually and pneumococcal vaccine is recommended before starting bDMARDs or if it has not been administered before, then every 5 years. Diphtheria Tetanus Polio vaccination is repeated every 10 years.*<br>*Concerning permitted vaccines, there is no risk of having greater adverse reactions when treated with bDMARDs.*	❑

**Be aware that tooth extractions require the discontinuation of bDMARDs.**<br>*Message: in case of usual cavity treatment or regular scaling: no need stop bDMARDs. The dentist will decide whether to prescribe antibioprophylaxis. In the case of an implantation, no need to stop bDMARDs, antibioprophylaxis will be discussed.*<br>*In the case of extraction, bDMARDs will be stopped in advance. After the procedure, bDMARD will be started again after complete healing and in the absence of infection.*	❑
**Know that the dentist must be informed about bDMARDs treatment.**<br>*Message: provide a leaflet to the dentist*	❑
**Know that scheduled surgery requires the discontinuation of the bDMARD.**<br>*Message: the delay before the surgery depends on the type of bDMARD and the type of surgery. Ask the rheumatologist. In case of emergency inform the surgeon and the anesthetist of the increased risk of infection*	❑
**Know that the surgeon must be informed in case of surgery when taking bDMARDs.**<br>*Message: in case of scheduled surgery, the bDMARD must be stopped. The rheumatologist is best able to tell the delay depending on the type of surgery and the type of bDMARDs.*<br>*After surgery, bDMARD will be started again when the scar has completely healed and in the absence of infection.*	❑
**Know that the anesthetist must be informed of the bDMARDs treatment.**<br>*Message: see surgery.*	❑
**Know that contraception is necessary for the couple.**<br>*Message: a pregnancy must be planned.*<br>*For women, anti TNF can be maintained until conception. For abatacept, Tocilizumab, Rituximab, stop before conception according to the current state of knowledge, so this recommendation should be updated by discussing it with your rheumatologist. Methotrexate should be stopped at least 1 month (one menstrual cycle) before conception.*<br>*For men, anti-TNF drugs can be maintained. For Abatacept, Tocilizumab, Rituximab: stop before conception according to the current state of knowledge, so this recommendation should be updated by discussing it with your rheumatologist. Methotrexate should be stopped at least 3 months before conception.*<br>*Overall, when a pregnancy is planned, discuss this with the rheumatologist as knowledge is evolving rapidly.*	❑
**How to plan a pregnancy under bDMARD.**<br>*Message: a pregnancy must be planned. See above.*	❑
**Know the rules of asepsia when injecting subcutaneous bDMARD.**<br>*Message: to make the self-injection: take the drug out of the refrigerator for 30 minutes if the ambient temperature is above 25° and 3/4h-1 hour if the temperature is below 25°. Wash your hands well, disinfect the skin, inject on the abdomen or thighs, change injection sites regularly; throw the used needle or pen into the collector, note the date of the injection. Do not inject where there is a wound or spots on the skin.*	❑
**Know how to maintain the cold chain.**<br>*Message: bDMARD must be stored between 3° and 8°. It should not be put in the freezer. The products usually last for several hours outside the refrigerator, ask for information.*<br>*Message: In case of travel, there are refrigerated cases for transport (provide it), French-English medical certificates for customs passage. For air travel, contact the airline company if necessary.*	❑
**Know how to dispose of used needles.**<br>*Message: Throw the used needle or pen into the collection container and bring the container to the pharmacy or the waste disposal center.*	❑
**Know who to contact in case of a problem when taking treatment**.<br>*Message: the health professionals to contact are: the attending physician who, if necessary, will contact the rheumatologist, the rheumatologist, the rheumatology nurse: provide the telephone number and/or e-mail of the department.*	❑

**Know how to communicate with the rheumatologist about joint pain, stiffness and swelling for the follow-up of rheumatism.**<br>*Message: bDMARDs have a delay of action of up to 3 months and sometimes 4 to 6 months. bDMARDs require regular monitoring by the rheumatologist. Only the rheumatologist can renew prescriptions.*	❑
**Know how to inform of bDMARD treatment any health professional.**<br>*Message: It is necessary to inform health professionals about treatment with a bDMARD. Only health professionals should be informed of the treatment and not the employer or other person.*	❑

6) **Document 6 Patient's Self-assessment (will stay in the center)**

At the end of the interview, the nurse will orally ensure that the patient has understood the notions. For this she will ask the patient to fill in the following small form. The presentation of this table will not be that of an "academic" audit but a self-assessment by the patient himself allowing him to ask additional questions.

"At the end of this interview, here are the messages we discussed together. Do you think that you have understood or would you like more information?

Competencies addressed	I think I understand	I would like more information
Naming biologic treatment	❑	❑
Fever, infections, antibiotics	❑	❑
Vaccines	❑	❑
Dental care	❑	❑
Surgery	❑	❑
Child conception	❑	❑
Who to call	❑	❑
Follow-up	❑	❑
Make the self- injection, aseptic measures	❑	❑
Travel	❑	❑
Cold chain maintenance	❑	❑

7) **Document 7 Skills evaluation (will be sent to the Research clinical Unit)**

The education session was carried out completely yes❑no❑

Competencies addressed	discussed	acquired
Naming biologic treatment	*❑yes no❑*	*❑yes no ❑❑to be re-evaluated*
Fever, infections, antibiotics	*❑yes no❑*	*❑yes no ❑❑to be re-evaluated*
Vaccines	*❑yes no❑*	*❑yes no ❑❑to be re-evaluated*
Dental care	*❑yes no❑*	*❑yes no ❑❑to be re-evaluated*
Surgery	*❑yes no❑*	*❑yes no ❑❑to be re-evaluated*
Child conception	*❑yes no❑*	*❑yes no ❑❑to be re-evaluated*
Who to call	*❑yes no❑*	*❑yes no ❑❑to be re-evaluated*
Follow-up	*❑yes no❑*	*❑yes no ❑❑to be re-evaluated*
Making the self- injection, aseptic measures	*❑yes no❑*	*❑yes no ❑❑to be re-evaluated*
Travel	*❑yes no❑*	*❑yes no ❑❑to be re-evaluated*

Cold chain maintenance	*❑yes no❑*	*❑yes no ❑❑to be re-evaluated*

At the end of the interview, the nurse advises :

- self-injection by the patient himself yes❑no❑
- the doctor's prescription for the injection by a private or hospital nurse yes❑no❑
-

8) Total length of educational session I__I__I minutes

9) BioSafe brochure delivery yes❑no❑

10) Free summary of the interview

At 3 months

11) Document 8 semi-structured Interview schedule at 3 months will remain in the center)

The nurse will inquire about events since BL, effect of bDMARDs, the patient feedback according to his/her expectations and potential discrepancy between the expectations and the treatment benefits , adverse events and patient's attitude towards these adverse events and his/her degree of motivation.
Use open-ended questions. Active listening and reflection techniques. Always encourage the patient.

How has the treatment been going since baseline?

How was the self-injection? Do you think you will be able to continue the self-injection?

Did you benefit from the treatment? How did you respond?

Have you had any side effects?

What did you do when they happened?

What do you think about biologics now?

The nurse must evoke situations of daily life that have not been spontaneously evoked with the patient to check if the acquired skills have been mobilized on this occasion: in particular: fever, infection, cough, nasopharyngitis, wound, dental care, contact with health professionals, etc.

12) Document 9 Intervention checklist (will remain in the center) filled out only for nurses who have not used the interview guide

The following items were discussed
Self-injection: realization/ motivation yes❑no❑
Benefits / Expectations / Patient feedback yes❑no❑
Adverse events /patient's attitide yes❑no❑
Everyday situations /patient's attitudes yes❑no❑

Anticipation of potential difficulties yes❑no❑

13) **<u>Document 10 intervention self-assessment</u>**

Type of interview carried out by IDE: free❑semi-structured❑

Does the patient perform the injections himself? yes❑no❑

The interview "IDE opinions-motivations" was carried out completely yes❑no❑

If not, it was difficult to address the following themes (multiple responses possible)

❑ Self-injection: realization/ motivation
❑ Benefits / Expectations / Patient feedback
❑ Adverse events /patient’s attitude
❑ Everyday situations /patient's attitudes
❑ Anticipation of potential ssible difficulties

14) Document 5 Safety checklist = the same as at BL with repeated messages. During this chesklist, the nurse will ask open-ended questions to see if the patient has acquired the knowledge and skills needed for biotherapy.
15) (document 7) Skills evaluation on the quality of its intervention or possible difficulties which will be sent to the Clinical Research Unit. The same as at baseline plus the following information:

Nurses’ Report to the doctor of situations requiring his intervention (will remain in the center)

❑ discontinuation of treatment
❑ non-compliance
❑ any event of which the doctor should have been informed by the patient and was not…………………
...

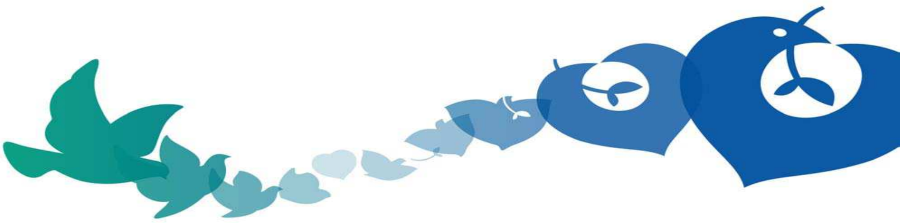

Efficacy of a nurse-led patient education intervention in promoting safety skills of patients with inflammatory arthritis treated with biologics.
BIOSAFE Study

This research is organized by Assistance Publique - Hôpitaux de Pa[illegible]
Département de la Recherche Clinique et du Développement
1 avenue Claude Vellefaux
75010 Paris

BIOSAFE

INFORMATION NOTE

Madam, Miss, Sir,

Doctor .... (name, first name), practicing at the hospital ...., proposes you to participate in a research concerning your disease. It is a study in current care in accordance with the law n°2004/806 of August 09, 2004, decree of March 9, 2007 of the public health code.

It is important to carefully read this note before making your decision; do not hesitate to ask for explanations.

If you decide to participate in this research, you will be asked for written consent.

1) Purpose of this research

The French National Health Authority (HAS) recommends the use of therapeutic patient education (TPE) in the management of chronic diseases such as chronic inflammatory arthritis (IA). More specifically, the French Society of Rheumatology (SFR) recommends TPE for rheumatoid arthritis and spondyloathritis.

Therapeutic patient education (TPE) aims to help patients acquire specific skills to better manage their chronic disease on a daily care. Although still underdeveloped, IA patients are the most frequently included in TPE programs in rheumatology in France.

To date, no study has evaluated the benefit of the therapeutic education approach on patient safety skills and the reduction of the risk of complications related to biologics.

This research concerns the efficacy of a nurse-led therapeutic education (TPE) for patients treated with biologics for chronic IA.

It aims to evaluate the impact of a medical consultation associated with a consultation with a nurse in comparison with a medical consultation alone, in the patients' acquisition of safety skills with regard to their treatment and their disease.

The strategy applied to each patient will be done by random drawing.

To answer the research question, it is planned to include 120 people with chronic inflammatory arthritis (rheumatoid arthritis, spondyloarthritis) who will start a biologic treatment in the rheumatology departments of 10 hospitals in France.

2) Research schedule

The research will last 18 months and your participation will be for 6 months.

After signing your consent, during the first visit, you will fill in questionnaires on your health status, then according to the random draw, you will benefit from:

- Either your usual medical consultation associated with a consultation with a nurse who will perform the TPE session at baseline and 3 months later,
- Or your usual medical consultation only, without nursing consultation

In all cases, you will be followed as usual by your rheumatologist.

At the end of 6 months, during the last protocol visit, you will fill in 5 questionnaires again and you will have the consultation with the nurse, if according to the random draw, the consultation with the nurse has not already done previously.

3)What are the benefits and constraints of your participation?

The expected benefit of this research is the improvement of safety skills regarding biologics in patients with chronic IA to better avoid complications. By participating in this research, you will not be asked to pay any additional costs. In addition, you will contribute to a better understanding of the role of the nurse, as a complement to medical consultations, in helping patients manage biologic treatment, particularly to avoid complications.

If you agree to participate, you will be expected to:

- Come to the appointments with the nurse according to the random draw and to the evaluation appointment at 6 months. If you are unable to attend, please contact the department as soon as possible.
- Inform the doctor of the research, of the use of any medication as well as of any event occurring during the research (hospitalization, pregnancy, etc.)

- Bring back the documents specific to the research
- Not to take part in another research project without your doctor's approval
- Be affiliated to a social security system or be a beneficiary of such a system

4) Potential risks of the research

This research is not a priori likely to cause any serious or non-serious adverse events. The expected serious and non-serious adverse events are those that may occur with the biologic treatment.

5) Destination of research samples

Non applicable for this study.

6) Destination of the data collected for the research

Within the research framework, in which the AP-HP proposes you to participate, a processing of your personal data will be implemented to allow the analysis of the research results with regard to the research objectives which has been presented to you.

To this end, your medical data, including your lifestyle data will be identified by a code number and your initials. These data may also be transmitted to the French health authorities under conditions that ensure their confidentiality.

If you wish to stop your participation without withdrawing your consent, the data collected prior to this stop will be used unless you do not wish it.

In accordance with law regulations and those of the National Data Protection Commission (Commission Nationale pour Informatique et Liberté (CNIL)) the research file has been authorized by the CNIL and you will have the right to access and rectify your personal data. You will be able to exert your rights with the help of your treating doctor in the context of the research. Only he/she is aware of your identity.

You also have the right to object to data transmission covered by professional secrecy that may be used and known in the context of this research.

7) Supervision of the research

The AP-HP has obtained the approval of the Comité de Protection des Personnes Ile-De-France XX for this research on 22/06/2016.

8) Your rights ?

Your participation in this research is entirely free and voluntary. Your decision will not affect the quality of care and treatment you are entitled to expect.

You will be able to ask for explanations on the progress of the research to the doctor who is treating you.

You may withdraw from the research at any time without justification, without any consequence on the continuation of your treatment or the quality of the care that will be provided to you, and without any consequence on the relationship with your doctor. At the end of this withdrawal, you can be followed by the same medical team.

In accordance with the regulations of the CNIL (National Data Protection Commission (Commission Nationale pour Informatique et Liberté), you have a right of access and rectification. You also have the right to object to the transmission of data covered by professional secrecy that may be used in the context of this research and to be processed. These rights can be exercised with the doctor in charge of the research who alone is aware of your identity. You may also access to all your medical data directly or through a doctor of your choice, in accordance with the provisions of article L 1111-7 of the Public Health Code.

Your medical file will remain confidential and can only be consulted under the responsibility of the doctor in charge of your treatment as well as by the health authorities and by persons duly mandated by the AP-HP for the research and subject to professional secrecy.

At the end of the research and after analysis of the data related to this research, you may be informed of the overall results through the doctor who is treating you in the framework of this research.

If you agree to participate in the research after having read all this information and discussed all aspects with your doctor, you will have to sign and date the informed consent form at the end of this document.

CONSENT FORM

I, the undersigned, Mrs., Miss, Mr. (last name, first name).. freely agree to participate in the research entitled "BIOSAFE" organized by the Assistance Publique - Hôpitaux de Paris and proposed to me by Doctor (last name, first name, telephone)...................... ...., physician in this research.

- I have read the information note version of 17/02/2016 [4 pages] explaining the objective of this research, the way it will be carried out and what my participation will involve,
- I will keep a copy of the information note and consent,
- I have received appropriate answers to all my questions,
- I have had adequate time to make my decision,
- I understand that my participation is free and that I may interrupt my participation at any time, without incurring any responsibility or prejudice to the quality of the care I will receive. I will then indicate to my treating doctor whether or not I wish the collected data to be used until the moment of my decision,
- I am aware that my participation can also be interrupted by the doctor if necessary,
- Before participating in this research, I have undergone a medical examination adapted to the research, the results of which have been communicated to me,
- I understand that in order to participate in this research, I must be affiliated to a social security system or be a beneficiary of such a system. I confirm that this is the case,
- I have been informed that my participation in this research will last 18 months and that this implies that I will not be able to consider participating in another interventional research, without informing my treating doctor of the research,
- My consent in no way relieves the doctor who is following me in the research nor the AP-HP of all their responsibilities and I retain all my rights guaranteed by law.

Signature of the person participating in the research

Last name First name :

Date: Signature:

Signature of the physician

Last Name First Name:

Date: Signature:

This document must be drawn up in 3 copies, the original of which must be kept for 15 years by the research coordinator, the second given to the person giving consent and the third sent to the AP-HP in a sealed envelope at the end of the research.

eAppendix 2. Final version of the protocol (16.07.2018).

ASSISTANCE PUBLIQUE HÔPITAUX DE PARIS

Efficacy of a nurse-led patient education intervention in promoting safety skills of patients with inflammatory arthritis treated with biologics.

BIOSAFE

Research in Routine Care

Version N°4 of 16/07/2018
Project Code: K151201 / BCR ID No.: 2016-A00332-49

Principal Investigator: Dr. BEAUVAIS Catherine
Rheumatology Department
Saint Antoine Hospital
Tel: + 33 1 49 28 25 20
Fax : + 33 1 49 28 25 13
Email: catherine.beauvais@aphp.fr

Scientific Director: Pr GOSSEC Laure
Rheumatology Department
Pitié Salpêtrière Hospital
Tel : + 33 1 42 16 00 00
Fax : + 33 1 42 17 79 55
Email: laure.gossec@aphp.fr

List of physicians/referrals from participating centers: see Appendix 1.

Associated teams :
Methodology and statistical analysis: Pr SIMON Tabassome, A. ROUSSEAU, Dr L. BERARD
Unit for Clinical Research Unit (UCR) of the Groupe hospitalier de l'Est Parisien (UCR- East)
Saint-Antoine Hospital
184 rue du Faubourg Saint-Antoine, 75571 Paris cedex 12, France
Phone: + 33 1 49 28 22 02 / Fax: + 33 1 49 28 28 13
Email: alexandra.rousseau@aphp.fr / laurence.berard@aphp.fr
Structure in charge of research follow-up: TOUATI Amel
Unit for Clinical Research UCR of the Groupe hospitalier de l'Est Parisien (UCR-East)
Saint-Antoine Hospital
184 rue du Faubourg Saint-Antoine, 75571 Paris cedex 12, France
Phone: + 33 1 49 28 22 02 / Fax: + 33 1 49 28 28 13
Email: amel.touati@aphp.fr

Study Manager: Aurélie ZINDJIRDJIAN
Tel : 01 44 84 17 64 / Fax : 01 44 84 17 01
Email: aurelie.zindjirdjian@aphp.fr
Delegation for Clinical Research and Innovation
Saint Louis Hospital 75010 PARIS

Page de SIGNATURE D'UN PROTOCOLE de recherche en soins courants par l'investigateur COORDONNATEUR et le représentant du GESTIONNAIRE

Code de la Recherche en soins courants : K151201, BIOSAFE

Titre : Efficacité d'une éducation thérapeutique par un(e) Infirmier (ère) par l'acquisition des compétences de sécurité vis-à-vis des biothérapies par les patients traités pour un rhumatisme inflammatoire chronique

Version N°4 du 16/07/2018

La recherche sera conduite conformément au protocole et aux dispositions législatives et réglementaires

Version N°4 of 16/07/2018

The research will be conducted in accordance with the protocol and the legislative and regulatory provisions in force.

The Coordinating Investigator :

Dr BEAUVAIS Catherine

Rheumatology Department Date: ……………/……….…… . .

Saint-Antoin e Signature Hospital :

Tel: 01 49 28 25 20

Email: catherine.beauvais@aphp.fr

The scientific leader :

Pr GOSSEC Laure

Rheumatology Department Date: ………/……….…….

Pitié-Salpêtrière Signature Hospital :

Tel : 01 42 16 00 00

Email: laure.gossec@aphp.fr

The Director of DRCI :

Mrs. Florence Favrel-Feuillade

Public Assistance - Paris Hospitals

Interregional Delegation for Clinical Research Date: ……………/……….…… . .

Saint Louis Hospital Signature :

75010 PARIS

The research received a favorable opinion from the CPP Ile de France VI dated June 28, 2016.

TABLE OF CONTENTS

1. ABSTRACT 57
2. SCIENTIFIC RATIONALE - RATIONALE FOR THE STUDY 59
2.1 Current state of research knowledge 59
2.2 Qualification of the research 59
2.2.1 Evidence that the medical strategies that are the subject of the research, the practical procedures and methods used in the research are consistent with current practice 59
2.2.2 Elements justifying that the specific monitoring methods added by the research entail only negligible risks and constraints........ ***Erreur ! Signet non défini.***
2.3 Recruitment potential 61
2.4 Statement that the research will be conducted in accordance with the protocol, good clinical practice and applicable laws and regulations. 61
3. OBJECTIVES AND JUDGEMENT CRITERIA OF THE RESEARCH 62
3.1 Primary outcome 62
3.2 Primary outcome measure 62
3.3 Secondary outcomes 62
4. SELECTION CRITERIA 62
4.1 Inclusion criteria 62
4.2 Exclusion criteria 63
5. RESEARCH DESIGN 63
5.1 Type of study 63
5.2 Expected number of participating centers 63
5.3 National/international research 63
5.4 Duration of research 63
5.5 Description of measures taken to reduce and avoid biases 63
5.5.1 The design 63
5.5.2 Blinding and methods implemented for its maintenance, procedure for unblinding 63
5.6 Rhumatology nurse training in patient education 63
5.7 Simultaneous participation in other research 64
6. PROGRESS OF THE RESEARCH 65
6.1 Scheme of the study 65
6.2 Experimental design – practical implementation 65
6.2.1 Selection visit 65
6.2.2 Inclusion visit (BL) 66
6.2.3 Research Follow-up Visit : 3 months 66
6.2.4 End of Research Visit 67
6.2.5 Summary table of patient follow-up 68
6.2.6 Examination and sample management 68
6.2.7 Building a biological collection 68
6.3 Rules for permanent or temporary cessation of research 69
6.3.1 Discontinuing an individual's participation in research 69
6.3.2 Discontinuing part or all of the research 69
6.3.3 Methods and timeframe for collecting these data 69
6.3.4 Methods of monitoring these people 69
7. AUTHORIZED AND PROHIBITED DRUGS AND TREATMENTS 69
8. DATA MANAGEMENT 69
8.1 Right of access to source data and documents 69
8.1.1 Data Access and Confidentiality 69
8.1.2 Source documents and data 70
8.2 Quality control and quality assurance 70

8.2.1 Guidelines for data collection......70
8.2.2 Quality control......70
8.2.3 Audit......71
8.3 Data Processing and Retention of Research Documents and Data......71
8.3.1 Data processing......71
8.3.2 Retention of Research Records and Data......71
9. STATISTICS......71
9.1 Justification of sample size......71
9.2 Description of the planned statistical methods, including the schedule of planned interim analyses.....72
9.3 Taking into account missing data......73
9.4 Analysis Populations......73
10. MANAGEMENT OF ADVERSE EVENTS......73
10.1 Adverse events (i.e., complications) related to medical procedures, combinations of procedures or strategies for prevention, diagnosis or treatment that are part of routine care (non-drug) excluding medications. .
10.1.1 Adverse events (i.e., complications) related to the care evaluated in the research study......74
10.1.2 Care-related adverse event (i.e., complication) reporting pathway......74
10.2 Adverse events with special follow-up by the manager......74
10.3 Adverse events related to the specific surveillance modalities added by the research......74
11. MODALITIES OF INFORMATION OF PERSONS......74
11.1 Information of persons......74
12. ETHICAL AND LEGAL ASPECTS......74
12.1 Declaration that the research will be conducted in accordance with the protocol and applicable laws and regulations.......74
12.2 Ethical assessment of the special monitoring methods provided for in the protocol......75
12.3 Legal requirements (Role of the manager, CPP, CCTIRS, CNIL)......75
12.3.1 Role of the Manager......75
12.3.2 Submission to the CPP......75
12.3.3 Notice from CCTIRS and authorization form to the CNIL......75
12.3.4 Substantial modification to the protocol......75
12.3.5 Final research report or publication......75
12.3.6 Data ownership......76
12.3.7 Publication Rules......76
13. BIBLIOGRAPHICAL REFERENCES......76
14. APPENDICES......79
14.1 Appendix 1 : List of participating centers and investigators in June 12, 2017......79
14.2 Appendix 2 : Questionnaires......80
14.2.1 BIOSECURE Questionnaire......80
14.2.2 SF 12......84
14.2.3 BMQ......
14.2 4 RAID: Rheumatoid Arthritis Impact of Disease......87
14.2.5 Arthritis helplessness index......89
14.2.6 Disease activity score DAS 28......90
14.2.7 BASDAI Bath Ankylosing Spondylitis Disease Activity Index......90
14.2.8 ASDAS......91
14.3 Appendix 3 : Nurse Education Booklet......**Erreur ! Signet non défini.**

15. ABSTRACT

Introduction: Chronic inflammatory rheumatic diseases (inflammatory arthritis [IA]) (rheumatoid arthritis (RA) and spondyloarthritis (SpA) are serious and disabling conditions that affect approximately 600,000 people in France. The prognosis of IA has been substantially improved by biologic disease-modifying anti-rheumatic drugs (bDMARDs), such as anti-TNF alpha, but these bDMARDs can have serious complications, particularly infections of the upper respiratory tract and pneumonia, with risk of tuberculosis and rare cases of opportunistic infections. The risk of serious infections is 5% per patient-year and is maximal in the first 6 months after the prescription of a first bDMARD. Therapeutic patient education (TPE) enables patients to acquire safety skills for managing infectious risks (e.g., stopping biologic therapy in case of fever). Skills acquisition is evaluated by the validated “BioSecure” questionnaire. Patient education on bDMARDs is mainly carried out by nurses and seemed to be effective in the acquisition of these skills in 2 uncontrolled studies. In 2010, a national survey of 677 patients showed that risk of giving wrong answers in the BioSecure questionnaire was 4-fold greater for patients who did not have TPE than those who had benefited from a consultation with a nurse or an educational approach (OR=3.8 95% CI: [1.68- 8.8]).

Hypotheses: Our hypothesis is that a face-to-face nurse-led patient education will allow for better acquisition of safety skills regarding bDMARDs at 6 months as compared with usual care (medical information during consultation at the time of bDMARD prescription).

Primary outcome: Acquisition at 6 months by IA patients of safety skills with regard to subcutaneously injected bDMARDs.

Secondary outcomes: Effect at 6 months of the nurse-led education on quality of life, disease activity, adherence, “coping” with the disease, psychological well-being, occurrence of serious infections related to treatment.

Primary outcome measurement: Response rate to the BioSecure validated questionnaire comprising 55 items, developed by the Patient education Group Section of the French Rheumatology Society, including safety skills concerning infections, vaccinations, and everyday life situations (travel, surgery, child conception etc.).

Secondary outcome measures: MMAS 4 Modified Morisky Adherence scale; Quality of life: SF-12; rate of severe infections defined as infections requiring hospitalization or intravenous antibiotics; coping with disease and psychological well-being (numeric rating scales) adapted from Rheumatoid Arthritis Impact (RAID) and Arthritis Helplessness Index (AHI); Disease Activity Score, ASDAS and BASDAI activity index.

Methods: Type of study and experimental design: Randomized open-label randomized controlled trial with blinded assessment.

The protocol will be proposed by the rheumatologist during a routine consultation when the decision is made to start bDMARDs. The self-administered questionnaire will be completed after receipt of non-opposition to the survey and before randomization.

Intervention group: usual consultation and information by the doctor + 2 TPE sessions by a trained nurse at a 3-month interval and follow-up by the treating rheumatologist. The first TPE session at baseline (BL), lasting a maximum of 1.5 hr, will include evaluation of the patient’s experience and knowledge, internal and external resources to deal with the disease and treatments, teaching of the subcutaneous (SC) self-injection, what to do in case of fever, and adjusting bDMARD treatment in risk situations. At 3 months, the second TPE session will include adaptation of educational messages to the patient’s feedback and

acquisitions. The TPE intervention will be standardized by consensus and a brochure will be provided based on the results of the BioSecure study.

Control group: usual consultation and information from the doctor and follow-up by the treating rheumatologist.

The primary outcome (BioSecure score) and self-administered questionnaire data will be assessed at the end of the study at 6 months in all patients during a consultation at the hospital by a health professional different from the nurse who has performed patient education. A TPE session will be offered at 6 months apart from the protocol for patients in the control group.

Legal characteristics of the project: research in routine care

Number of patients needed: 129

Inclusion criteria: Age 18 to 75 years,

Rheumatoid arthritis (ACR/EULAR criteria) or axial or peripheral spondyloarthritis (ASAS criteria) including psoriatic arthritis. Indication for a first subcutaneous bDMARD (according to the current French recommendations for IA management), patients bDMARDS naïve,

Ability to complete a questionnaire, collection of non-opposition, beneficiary of a social security.

Exclusion criteria:

Severe psychiatric disorders or cognitive impairment.

Total study duration: 21 months (15 months inclusion and 6 months patient participation)

Inclusion period: 15 months

Length of participation for one patient: 6 months

Number of participating centers: 11

Average number of inclusions per month per center: 1

16. SCIENTIFIC RATIONALE – RATIONALE FOR THE STUDY

16.1 Current state of knowledge with regard to research

Chronic inflammatory arthritis (IA) (rheumatoid arthritis [RA] and spondyloarthritis [SpA]) affect approximately 600,000 people in France. When insufficiently controlled by treatment, these painful and disabling conditions affect quality of life and ability to work, eliminating patients from employment. bDMARDs (biological disease-modifying anti-rheumatic drugs) are highly effective treatments for IA and are increasingly used. In 2010, an estimated 30% IA [1] patients in France were treated with bDMARDs, mainly with TNF alpha blockers administered in SC injections. The bDMARDs' treatment rate in IA population is certainly higher nowadays. However, bDMARDs are at risk of serious complications, particularly infections, such as pulmonary infections, tuberculosis and some cases of opportunistic infections [2]. The risk of infections related to bDMARDs is estimated to 2-folds risk vs conventional DMARDs [2]. The risk of severe infection is estimated at 5%/patient-years and is highest in the 6 months following the first bDMARD [3].

Therapeutic patient education (TPE) is recommended to help patients acquire specific skills to better manage their chronic disease in everyday life [4]. TPE is also recommended in the management of IA [5,6]. Among the required skills are safety skills aimed at preserving the patient's life by avoiding complications termed 'life-saving self-care skills' [4]. Safety skills can be assessed by the validated BioSecure questionnaire, a 55-item questionnaire assessing patients' skills in managing risk situations: fever, infections, vaccinations, travel, surgery, pregnancy [7]. The BioSecure questionnaire is also widely used in routine care in the management of IA. TPE on bDMARDs seems to be effective on safety skills in one uncontrolled (abstract) study [8], while another uncontrolled study showed results in favor of group TPE [9]. Moreover, in 2010 a national survey showed that the number of wrong answers to the BioSecure questionnaire was 4-folds higher in patients who did not benefit from TPE (at least a consultation by a nurse) (OR=3.8 IC95% [1.6-8.8]) [10,11]. In this 2010 study, only 30% of patients had received a nurse-led TPE and 11% had access to TPE sessions [12].

Since 2010, the number of nursing consultations dedicated to IA has increased (see below) but no study has evaluated the benefit of this approach on safety skills.

In this context, the aim of this single-blind, multicenter, randomized trial is to evaluate the impact of an education by a nurse on safety skills with regard to bDMARDs. Our hypothesis is that the education group will have better skills at 6 months than the control group.

16.2 Qualification of the research

This protocol is part of a research project aimed at evaluating routine care as defined by law n°2004-806 of August 9, 2004 relating to public health policy and by its application decree (n° 2006-477) of April 26, 2006. (Reference texts: articles L.1121-1, 2nd paragraph and R1121-3 of the Public Health Code).

16.2.1 Evidence that the medical strategies that are the subject of the research, the practical procedures and methods used in the research are consistent with current practice.

TPE is recommended in the management of chronic diseases [4] such as IA, and specifically by the French Rheumatology Society [5,6] for rheumatoid arthritis and spondyloarthritis. Although TPE is still insufficiently developed [10], IA patients are frequently included in TPE rheumatology programs in France. Indeed, of the 165 TPE programs authorized in rheumatology and listed in 2012 by Regional Health Agencies, 80 were targeted to IA patients [12]. However, evaluation of the efficacy of these programs has not been performed to date and is one of the barriers to the development of TPE for these patients.

The TPE nurse-led consultation usually lasts a maximum of 1.5 hours and follows the medical decision for a bDMARD and initial information by the doctor on benefits/risks of the treatment, the expected positive effects and the main adverse events. TPE is usually carried out by trained nurses.
The consultation by the nurse, usually done with a semi-structured interview schedule, uses a framework in accordance with the recommendations of the High Health authority:

- Evaluation of the patient's experience and knowledge of his/her illness.
- His/her internal and external resources to deal with the disease.
- Information and education on bDMARDs including teaching self-injections and safety warning signs.
- If the patient does not feel able to perform self-injection, the nurse will suggests that the doctor prescribes a nurse in private practice for the first injections.

In routine care, bDMARDs are prescribed by the rheumatologist during the consultation in hospital. The rheumatologist provides information on the treatment benefits/risks, on the expected positive effects and the main side effects and warning signs. The rheumatologist prescribes the first injections by a nurse if necessary.

After initiation of the bDMARD, the patient is routinely followed-up by the treaty physician at 3 months for evaluation of treatment efficacy according to EULAR (European Alliance of Associations for Rheumatology) response criteria. In case of efficacy, the treatment is continued until 6 months when a new evaluation is performed. In case of insufficient response or non-response, a switch to another bDMARD is provided at 3 months.

16.2.2 Evidence that the specific monitoring methods added by the research involve minimal risk and constraints.

As part of the proposed research, will be added :

- one TPE consultation by a nurse at baseline (BL) and at 3 months in the intervention group
- the filling-up in of self-administered questionnaires in both groups at BL and at 6 months.

All of the nursing consultations and self-administered questionnaires filling-up will be carried out during the usual consultations scheduled for these patients as part of the follow-up of their disease according to the recommendations of the French Rheumatology Society [5,6].

**Monitoring procedures in the usual care framework**	**Monitoring procedures <u>added by the research</u>** (Additional procedures compared to the usual care)
-Baseline : medical clinical examination	-In the intervention group: TPE consultation by a nurse at baseline and 3 months.

-3 months : clinical and biological monitoring examination with regard of bDMARDs by the treating rheumatologist<br>-6 months: clinical and biological monitoring examination with regard of bDMARDs by the treating rheumatologist<br>-	-In both groups: filling-in of self-administered questionnaires at baseline and 6 months.

Table 1: Specific monitoring modalities carried out as part of usual care / added by research

16.3 Recruitment potential

Last name, First name	Country, City, Hospital	Specialty	Recruitment / month	Total	**Center number**
Catherine Beauvais	France, Paris, CHU Saint Antoine	Rheumatology	*1*	*12*	**001**
Laure Gossec	France, Paris, CHU Pitié Salpétrière Hospital	Rheumatology	*1*	*12*	**002**
Françoise Fayet<br>Martin Soubrier	France, Clermont-Ferrand, CHU Gabriel Montpied Hospital	Rheumatology	*1*	*12*	**003**
Laurent Grange	France, Hopital Sud Echirolles, CHU Grenoble	Rheumatology	*0.5*	*6*	**004**
Anne-christine Rat	France, Nancy, CHU Brabois	Rheumatology	*0.5*	*6*	**005**
Yves Maugars<br>Marie Pierre Aubert	France, Nantes, CHU	Rheumatology	*1*	*12*	**006**
Aleth Perdriger	France, Rennes, CHRU Pontchaillou	Rheumatology	*1*	*12*	**007**
Sophie Pouplin	France, Rouen, CHU University Hospital of Rouen,	Rheumatology	*1*	*12*	**008**
Christelle Sordet.	France, Strasbourg, CHU Strasbourg	Rheumatology	*1*	*12*	**009**
Béatrice Pallot Prades	France, Saint Etienne, CHU Saint Etienne , Bellevue Hospital	Rheumatology	*1*	*12*	**010**
Isabelle Griffoul-Espitalier	France, Tours, CHRU Trousseau	Rheumatology	*1*	*12*	**011**
Total			11	120	

16.4 Statement that the research will be conducted in accordance with the protocol, good clinical practice and applicable laws and regulations.

I, the undersigned, Dr Catherine BEAUVAIS, certify that the research that I will coordinate, will be conducted in accordance with the protocol, the good clinical practices and the legislative and regulations in France.

17. OBJECTIVES AND OUTCOME CRITERIA OF THE RESEARCH

17.1 Primary outcome.

Evaluate the efficacy at 6 months of a nurse-led education session on the acquisition of safety skills by IA patients regarding bDMARDs.

17.2 Primary outcome measure

Safety skills measured at 6 months by the BioSecure validated questionnaire comprising 55 items, developed by the therapeutic education Section of the French Society of Rheumatology.
This questionnaire assesses safety skills related to infections, vaccinations, and everyday life situations (travel, surgery, child conception) [Appendix].

17.3 Secondary outcomes

3) Secondary outcomes
   - Efficacy of TPE with regard of to the official objectives of the High Health Authority that is quality of life
   - Severe infections related to treatment
   - “Coping” with the disease, psychological well-being
   - Correlations between gained skills and patient characteristics

4) Outcome measures
   - Quality of life: difference between baseline and 6 months of MOS-SF-12 questionnaire SF 12 [15]
   - Severe infections rate occurring within 6 months of initiation of bDMARDs, defined as infections requiring hospitalization or intravenous antibiotics.
   - Coping with disease: difference between baseline and 6 months in coping (from Rheumatoid Arthritis Impact of Disease [RAID]) measured by a numeric rating scale (NRS) [16] and the Arthritis Helplessness Index (AHI) [17].
   - Psychological well-being: difference between baseline and 6 months in psychological well-being (from RAID) measured by an NRS [16]
   - Association between responses to the BioSecure questionnaire and patient characteristics: sex, age, education level, disease duration, underlying disease (RA, axial SpA).
   - Opinions regarding the treatment evaluated by the Beliefs About Medicines Questionnaire (BMQ) [26].

18. SELECTION CRITERIA

18.1 Inclusion criteria

- Age from 18 to 75 years
- RA (fulfilling the 2010 American College of Rheumatology [ACR]/ European League Against Rheumatism [EULAR] classification criteria ACR/EULAR criteria) or axial or peripheral SpA (fulfilling the 2009 Assessment of SpondyloArthritis international Society classification [ASAS] criteria) [24,25]
- Indication for a first subcutaneous bDMARD (according to the usual French recommendations of care), therefore bDMARD-naïve
- Ability to complete a questionnaire
- Collection of non-opposition to participation in the study

- Beneficiary of social protection or an entitled person

18.2 Exclusion criteria

- Any condition that may affect understanding or adherence to treatment (chronic alcoholism, language barrier, severe psychiatric disorders)
- Intension for intravenous bDMARDs
- Already received therapeutic education about bDMARDs

19. RESEARCH DESIGN

19.1 Type of study

Randomized, multicenter, open-label, study with blinded evaluation of the primary outcome.

19.2 Expected number of participating centers

Multicentric. 11 participating centers.

19.3 National/international research

National Research

19.4 Duration of the research

Total study duration: 21 months
Inclusion period: 15 months
Participation duration for one patient: 6 months

19.5 Description of intended measures to reduce and avoid biases

19.5.1 Randomization

The unit of randomization will be the patient. The randomization will be carried out during the medical consultation, after the collection of the non-opposition and after the filling-in of the self-administered questionnaires, by Internet via the Cleanweb software.
Randomization will be stratified by center and balanced by blocks that will not be communicated to the investigating team. The ratio will be 1:1.
A randomization number will be assigned (consisting of 2 random letters and 6 random numbers) will be reported in the CRF (case report from).

19.5.2 Blinding and methods for binding maintenance, procedures for unblinding.

Due to the type of intervention, double-blinding will be impossible. However, patients in the intervention group will not be in contact with the education nurse during the 6-month assessment to ensure that the primary outcome is blinded to the randomization arm.

19.6 Rheumatology nurses training in patient education

In order to ensure that all nurses have the experience and qualifications required to conduct the TPE sessions in accordance with the High Health Authority and French Society of Rheumatology recommendations, it has been ensured that at least 1 nurse from 8/10 of the participating centers has a university degree in TPE. This degree in TPE for osteoarticular diseases (Université Pierre et Marie Curie

- Paris VI) was developed by the TPE Section of the French Society of Rheumatology. Other nurses will have a TPE training of at least 40 hours.
In addition, all investigating centers will prove an authorization to conduct TPE programs for chronic inflammatory rheumatic diseases, delivered by Regional Health Agencies.

This will ensure TPE standardization among the participating centers.

In addition, a semi-structured interview brochure will be provided to all nurses. This brochure is derived from the semi-structured schedule questionnaire called "de Rouen" developed by the Rheumatology Department of the Rouen University Hospital and widely used.

19.7 Simultaneous participation in other research

Participation in non-interventional patient research is possible.
There is no exclusion period for participation in intervention research after the end of participation in the Biosafe study.

20. PROGRESS OF THE RESEARCH

20.1 Scheme of the study

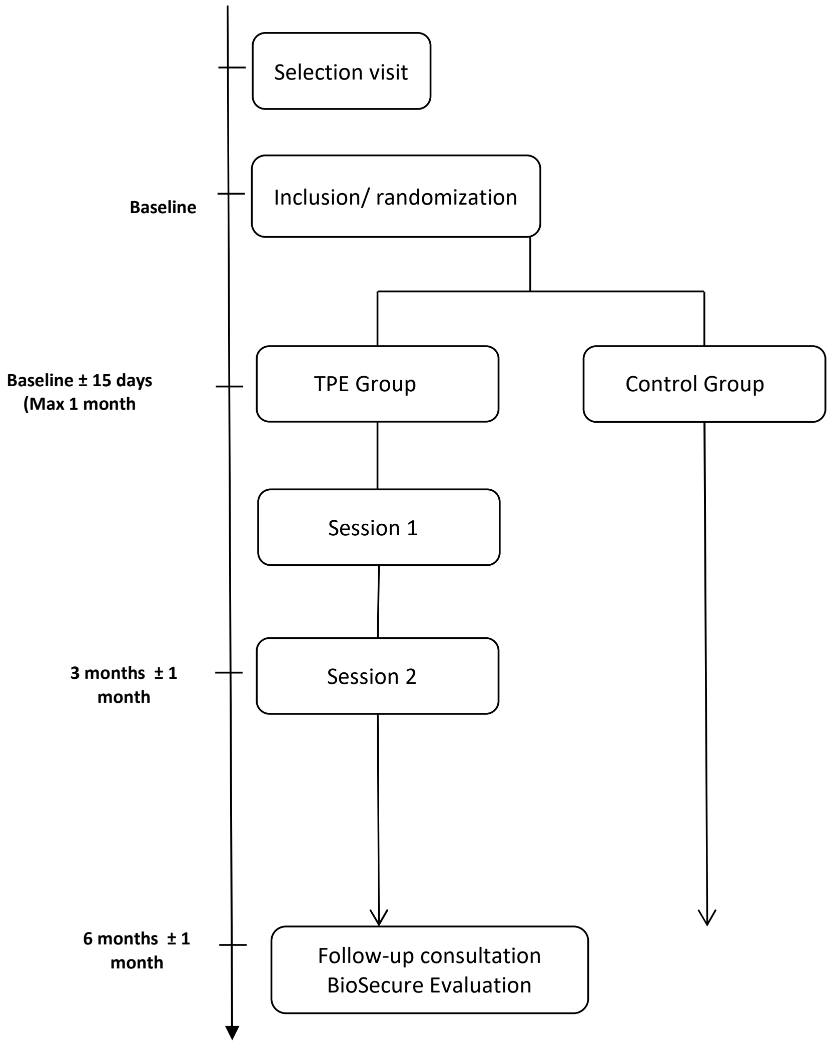

20.2 Experimental design - implementation

20.2.1 Selection visit

The pre-inclusion visit will take place during a medical consultation, i.e. either a regular consultation or a consultation during a day care hospitalization known as a "pre-biologic check-up" carried out by many investigating centers.

During the pre-inclusion visit, the physician will check the eligibility criteria (inclusion and exclusion criteria), propose to participate in the study and provide the written information note about the protocol. At the pre-inclusion visit, the physician will ensure that the health check-up for bDMARDS is scheduled for the bDMARD initiation at baseline. Otherwise, the physician will prescribe a biologic sample including at least Erythrocyte sedimentation rate and C reactive protein dosage for the baseline visit.

At baseline, Erythrocyte Sedimentation Rate and C reactive protein results will be collected, dating from less than 1 month. If not available, these dosages will be performed within 1 week, according to usual care before the initiation of a bDMARD.

20.2.2 Inclusion visit (Baseline)

The baseline inclusion visit will take place at the time of or after the pre-inclusion visit depending on the department's procedures and the patients' preferences.
In addition to the usual clinical examination, the inclusion visit will include:

- Information by the physician on bDMARD and safety according to standard care.
- Patient’s non-opposition after checking of the inclusion and exclusion criteria, particularly the absence of counterindications to bDMARDs according to the results of biological sample less than one month old.
- Patient completion of self-administered questionnaires

The patient will then be randomized (via the Cleanweb software) into one of 2 groups: Intervention Group or Control Group.

The rheumatologist will then prescribe, as usual, bDMARD and the usual biological follow-up of bDMARDs, systematically including Erythrocyte sedimentation rate and C reactive protein dosage at 6 months.

Depending on the patient's randomization group, information on bDMARD will be different.

- Intervention Group

The nurse's intervention at baseline will take place on the day of inclusion or within 2 weeks after the inclusion visit, maximum 4 weeks later.
The baseline education session will last a maximum of 1h30. It will be standardized between centers and will include the following:
-Patients’ evaluation using a semi-structured questionnaire and interview.
-Messages to deliver about the safety of bDMARDs and appropriate behaviors in everyday situations.
-Motivation for bDMARDs and potential difficulties
-Sub-cutaneous injection only according to the centers' procedures. If the nurse concludes that the patient will not be able to perform self-injections, he/she will suggest a prescription for a nurse in private practice.

- Control Group

There will be no nurse intervention. A prescription for a nurse in private practice will be made according to the physician's habits, which will be collected in the Case report form (CRF) at 3 months.

20.2.3 Research follow-up visit at 3 months

All patients will be seen at 3 months according to usual care recommendations including monitoring of potential adverse events.
In the intervention group a nursing interview will be conducted (TPE at 3 months).
The visit at 3 months will be performed 3 months (± 15 days) after the baseline visit.
Education session will be standardized between centers and will include the following

- Free collection of the patient's experience and difficulties in the past 3 months.
- Review and reassurance of security messages
- Motivation enhancing

The intervention will be face-to-face. However, in case of repeated refusal by the patient for a face-to-face interview and close to the 4-month deadline, the visit at 3 months will be done by telephone call with the patient's consent.
If possible, the interview at 3 months will be done by the same nurse as at baseline.

20.2.4 End-of-research visit

The 6-month visit will concern both groups. It will take place at 6 months (±1 month) after inclusion.
In addition to the usual-care clinical examination, it will include:
- Completion of self-administered questionnaires by all patients. The questionnaires will be given to the patient by a member of the team blinded to the patient's randomization arm, i.e. a health professional other than the nurse and doctor seen at baseline and 3 months; the health professional can be, for example, another nurse, another doctor, etc. A check that all the items in the Biosecure questionnaire have been fully completed will be done by the health professional collecting the questionnaire. If the patient does not meet the minimum safety skills at 6 months, another TPE session will be scheduled as part of the usual care.
- Collecting of other data will be done in the same way as well as self-administrated questionnaires.

At the end of this visit, the patient's participation in the study will end.

The participating centers will then offer, apart from the protocol, an education session on bDMARDs to patients in the control group and/or will at least assess the patient's knowledge on bDMARDs to offer this session to patients with insufficient knowledge. It will be possible to perform this session after the end-of-research visit, apart from the protocol, only after the 6-months assessment has been completed.

If the patient does not attend to the end-of-research visit, the missing data at 6 months will be collected by the investigators and will include the following:

- New appointment scheduled within 1 month
- Or sending questionnaires by mail
- And collecting of the C-reactive protein sample and DAS28 score from the treating rheumatologists because these data are available as part of usual-care follow-up

20.2.5 Summary table of patient follow-up

**Data collection**	**BL-1**	**BL**	**3 months**	**6 months**
**Visits**	**V0**	**V1**	**V2**	**V3**
	**SELECTION**	**INCLUSION Randomization**		**Evaluation**
**Protocol information**	R			
**Selection criteria**	R	R		
**Collection of the non-opposition form**		R		
**Randomization in both groups**		R		
<u>Socio-demographic data</u><br>Current treatment<br><br><u>Biological examination (usual care)</u><br>ESR and CRP		 UC<br>UC<br><br>UC		 <br><br><br><br>UC
<u>Clinical examination</u>		UC		UC
Severe infections		UC (within the last 2 years)		UC (since BL)
**<u>Self-administered questionnaires:</u>**<br>overall level of information<br><br><u>Disease Activity</u><br>Patient opinion on disease activity for DAS 28 and<br>ASDAS calculation<br>BASDAI for all SpA		 <br><br><br>R<br>R<br>R		 <br><br><br>R<br>R<br>R
**BioSecure: primary outcome**<br>bDMARDs Injection by the patient her/himself				R<br><br>R
Quality of Life SF12 (Short form)<br>Coping NRS<br>Psychological well-being NRS<br>AIH<br>BMQ		R<br>R<br>R<br>R<br>R		R<br>R<br>R<br>R<br>R

Abbreviations: BL, baseline; R, research ; US, Usual Care; ESR, erythrocyte sedimentation rate; CRP, C reactive protein; DAS28, Disease activity score 28 joints; ASDAS, ASAS-endorsed disease activity score ;BASDAI, Bath Ankylosing Spondylitis Disease Activity Index SF12, MOS SF12 ; NRS, numeric rating scale; AHI, Arthritis Helplessness Index; BMQ, Beliefs about medication questionnaire;

Table 2: Summary table of research timeline for the patient

20.2.6 Management of examinations and samples

The trial does not involve research-specific specimens; specimens collected for routine patient follow-up will be collected and routed through the usual channels at each center.

20.2.7 Building a biological collection

Not applicable

20.3 Rules for permanent or temporary cessation of research

20.3.1 Discontinuing an individual's participation in research

Any individual may withdraw prematurely his/her participation in the research at any time for whatever reasons.
The treating physician may temporarily or permanently discontinue a subject's participation in research for whatever reason in the best interests of the subject.
In case of the following:

- Temporary discontinuation from the research: the patient will continue the study, the physician following the patient will document the discontinuation reason.
- Definitive withdrawal from the research: the patient will continue the research follow-up, until his/her participation ends; the investigating center or the treating rheumatologist will document the termination reason.

20.3.2 Discontinuation of part or all of the research

The manager of AP-HP (Assistance publique hôpitaux de Paris) will reserve the right to permanently suspend inclusions, at any time, if the objectives are not met in terms of number of inclusions.

20.3.3 Methods and schedule for collecting data

The data collected will be analyzed

20.3.4 Methods of follow-up

Patients will continue follow-up according to the usual care by their treating rheumatologists.

21. AUTHORIZED AND PROHIBITED DRUGS AND TREATMENTS

The drugs authorized and prohibited in this research are the same as those used in the usual care for IA.

22. DATA MANAGEMENT

The data will be collected on a paper CRF in each center. Duplicates of the CRFs will be sent to the UCR-East as soon as the patient will have completed the study with an envelope provided to each center.
The CRFs will be sent grouped and the data will be entered by a Clinical Research Technician (CRT) in a CleanWeb Telemedicine database accessible via the internet and developed by a data manager of Unit for Clinical Research (UCR-East).

22.1 Access right to data and documents sources

22.1.1 Data Access and Confidentiality

The research manager will ensure that the protocol and the information form to persons included in the research state that:

- the documents and individual data are strictly necessary for the follow-up, quality control and audit of research aimed at evaluating routine care.

- these documents and data will be available only to persons individually mandated for this purpose by the research manager.

The research manager will also ensure that each person involved in the research has not objected to access to his/her individual data.

All research activities must obey strict rules of confidentiality. The persons mandated for the research are bound by professional secrecy, in particular under the conditions defined by articles 226-13 and 226-14 of the Penal Code, in the same way as the investigators themselves.

During and at the end of the research, individual collected data that will be transmitted to the manager by the investigators (or any other research collaborators) will be coded. In no case should they clearly show the name of the persons concerned, nor their address, nor any other information allowing direct identification.

22.1.2 Source documents and data

The self-administered questionnaires (including BioSafe) will be considered as source data as well as the patient's medical record.

22.2 Quality control and quality assurance

22.2.1 Guidelines for data collection

All information required by the protocol must be recorded into the CRFs. These data must be collected and recorded as soon as they are obtained, and transcribed into the CRF in an accurate, complete and legible way.
Each missing data must be explained. Erroneous data found in the CRF will be clearly crossed out and the new data will be copied, next to the crossed-out information, with initials, date and possibly a justification by the person conducting and monitoring the research or the authorized person who made the correction.

Paper CRF

All the information required by the protocol will be provided into the CRF and an explanation will be given by the physician for each missing data. Data will be transferred into the CRF as they are obtained, whether clinical or paraclinical.
The data will be copied neatly and legibly with a black ballpoint pen into the CRFs to facilitate duplication and computer input.
Incorrect data found in the CRF will be clearly crossed out and the new data will be copied with initials and date by the research team member who will make the correction.
Subjects' anonymity will be ensured by a code number and the subject's initials will be ensured in all documents needed by the research, or by deleting by appropriate means nominative data from source documents.
The computerized data on file will be declared to the French data protection authority (Commission Informatique et libertés (CNIL)) according to the current procedures.

22.2.2 Quality Control

The investigator will make the documents and individual data strictly needed for the monitoring, quality control and audit of this research available to the persons in charge of quality control and duly mandated by the research manager.

Beauvais C, *et al. RMD Open* 2022; 8:e001828. doi: 10.1136/rmdopen-2021-001828

The person(s) mandated by the research manager will regularly visit each center, during the research implementation, one or more times during the research according to the rhythm of inclusions and at the end of the research.

During these visits, the following items will be collected:

- Subjects' protection and safety,
- compliance with the research protocol and procedures defined therein and by the current regulatory legislation.
- data quality collected in the CRF: accuracy, missing data, data consistency with source documents (medical records, appointment books, original laboratory results, etc.),
- management of products and samples.

All visits will be monitored with a written report.

22.2.3 Audit

An audit will eventually be carried out at any time by persons mandated by the manager independently of the research investigators. Its purpose will is to ensure the research quality, the results validity and compliance with the current regulatory legislation.

Those conducting and monitoring the research will agree to comply with requirements from the manager and authorities and will agree with an audit or inspection.

The audit can happen at all stages of the research, from protocol development to results' publication and data classification used or produced by the research.

22.3 Data processing and conservation of documents and research data

22.3.1 Data processing

Clinical data will be collected at each visit by the patient's physician in a paper CRF.
The CRF duplicate and questionnaires' copy will be mailed to the UCR-East) for data entry. The data will be entered into a CleanWeb database by a Clinical Research Technician (CRT) in the URC-East.

Data management (creation of the database on CleanWeb and data control according to a predefined validation planning via the SAS software) will be carried out by a URC-Est data manager.

The patient will be identified by the inclusion center number, his/her inclusion number and initials. The patient reference will be: center number - center inclusion number - last name initial - first name initial (|__|__|__|-|__|__|__|__|__|__|__|-S-N). (Surname, Name)

A randomization number will be assigned and reported in the CRF (consisting of 2 random letters and 6 random numbers).

Data processing will be under the responsibility of the UCR-East.

22.3.2 Retention of Research Documents and Data

Routine care research data and documents will be stored at the end of the research by the investigator and the manager for 15 years.

23. STATISTICS

23.1 Justification of sample size

The national survey conducted in 2010 [11] found an average BioSecure score in the non-TPE group of 68.09±18.28. Patients who reported they had had TPE had a score of 75.66±14.20. In our study, we expect

a difference of 10 points or 15% increase in score. Indeed, patients in the national survey were already treated with bDMARDs whereas patients included in our study will be bDMARDs naïve, with inclusion taking place at the initiation of treatment. The expected scores in the control group will therefore be lower. Under the hypothesis of a 15% relative increase in score with TPE, with alpha=5%, beta=20% and 25% non-assessable patients, 129 subjects should be included .

23.2 Description of the planned statistical methods, including the schedule of planned interim analyses

No intermediate analysis will be planned.
The analysis will be performed after the database freezing and blinded to the randomization group.
The analysis will be conducted in intention to treat population. The primary outcome will then be analyzed in per protocol population.
The analysis will be performed with SAS V9.3 software.

Description of the baseline characteristics

The patients baseline characteristics will be described in total and by randomization group. Qualitative data will be described by frequencies and numbers, and quantitative variables will be described by their means and standard deviations, ranges or medians and interquartile ranges.

Analysis of the primary outcome:
The primary outcome will be the patient's BioSecure score [7] at 6 months. The BioSafe questionnaire consists of 24 competencies assessed in two parts, one part common to patients with intravenous (IV) and subcutaneous (SC) bDMARDs and the second part only for patients with SC bDMARD.
Part 1, includes 8 questions and 6 case scenarios.
Part 2 includes 1 question and 1 additional case.
Each correct answer is associated with 1 point.
The competency score is calculated as the sum of the points obtained, reported to 100.

The mean value of the BioSecure score at 6 months (with distinction between SC and IV patients) will be compared by a Student t-test or a non-parametric test if necessary.

Secondary analysis
Secondary outcomes
The criteria will quality of life assessed by the SF12 questionnaire at BL and 6 months.

- The MOS-SF-12 questionnaire [15] is derived from the SF36 questionnaire.

It includes 12 items divided into eight dimensions (physical function, physical limitation, physical pain, emotional limitation, mental health, vitality, social functioning, overall perceived health).
The Mental Quality of Life (MQL) score and the Physical Quality of Life (PQL) score are then calculated only if all 12 items have been completed. Scores range from 0 to 100.
The scores at BL and 6 months will be described by groups.
Each score differences between BL and 6 months will be calculated and compared between groups, by a linear regression taking into account the BL score value.

Other Secondary Criteria

- Severe infections, defined as infections requiring hospitalization and/or intravenous antibiotics.

Treatment-related severe infections rate occurring within 6 months after initiating bDMARDS will be described and compared between groups using an accurate Fisher's test.

- Coping with the disease and psychological well-being will be assessed by a NRS. The obtained score will therefore be an integer between 0 and 10.

Scores will be described at BL and 6 months.

The difference between BL and 6 months will be calculated and compared between groups by a linear regression with account for the initial value. If difference distribution does not follow a normal distribution, the percentage of change will be calculated and compared between groups by a nonparametric Wilcoxon-Man Whitney test.

- Opinions about treatment, evaluated by the BMQ belief about medicine questionnaire

- The value of the BioSecure score will be described according to the type of bDMARD received (IV, SC).
- The value of the BioSecure score will be described according to: gender, age, education level, disease duration and underlying disease (Rheumatoid Arthritis or Axial Spondyloarthritis). The score values will be compared by a t-test or a non-parametric test for the qualitative variables. The correlation between scores and age will be assessed either by calculating a Pearson or by Spearman correlation coefficient if applicable. A coding of age into classes will be considered.

These last two analyses will be performed on the population per protocol.

23.3 Consideration of missing data

If the BioSecure score cannot be calculated (due to not collected questionnaires or questionnaire missing data), the patient will be considered a failure regardless of the randomization group. The score will be replaced by the 25 percentile value, calculated on the total population.

23.4 Populations for analysis

The ITT (Intention To Treat) population will consist of all randomized patients with no major protocol deviation. The per-protocol population will consist of randomized patients who have fully participated in the study, with no protocol deviation and whose BioSecure total score will allow calculations.

24. MANAGEMENT OF ADVERSE EVENTS

24.1 Adverse events (i.e., complications) related to medical procedures, combinations of procedures or prevention strategies, diagnosis or treatment that are part of the routine care (excluding medication).

In the context of the research aimed at evaluating routine care, medical procedures and strategies which are the subject of the research, will be part of usual care and delivered in accordance with their indications. Potential adverse events are therefore those related to the patient's usual care (care-related) and do not require specific reporting by the research manager.

24.1.1 Adverse events (i.e. complications) related to the care evaluated in the research.

24.1.2 Circuit for reporting adverse events (i.e. complications) related to care

These events will follow the usual reporting circuit provided by the current regulations.
For example:

- Adverse reactions that may be drug-related should be reported to the Regional Pharmacovigilance Centre.

24.2 Adverse events with special follow-up by the manager

Not applicable

24.3 Adverse events related to special monitoring added by research

Monitoring added by research to assess routine care involve negligible risks and constraints for the research subject. Therefore, no procedures for managing severe adverse events are imposed by the research.

25. PERSONS INFORMATION METHODS

In accordance with ***Law No. 2004-806 of August 9, 2004 on public health policy,*** the investigator has an ***obligation to inform individuals*** prior to their participation in research aimed at evaluating routine care.

25.1 Information of the person.

In accordance with article R. 1121-3 of the Code for Public Health, information for persons who entering into research is needed by delivering a written form, submitted in advance to the Ethical Committee (Committee for the Protection of the Persons CPP).
The patient will be informed (with a written form) by the rheumatologist in usual care. After a potential reflection time, the non-opposition form will be collected during the inclusion visit.

When research will be completed, the person participating in the research will be informed of the research overall results in the way specified in the information brochure.

26. ETHICAL AND LEGAL ASPECTS

26.1 Declaration that the research will be conducted in accordance with the protocol, current laws and regulations.

The manager and the person(s) directing and supervising the research will commit to ensure that this research is carried out in accordance with the law n°2004-806 of August 9, 2004 relating to public health policy and the current regulations. (Articles L1121-1, 2° paragraph and R1121-3 of the Public Health Code).
Research recorded data will be subject to computerized processing in compliance with the modified law n°78-17 of January 6, 1978 relating to data processing, files and freedom.
The research will be conducted in accordance with this protocol.

26.2 Ethical evaluation of the specific monitoring methods provided for in the protocol

The specific monitoring methods added by the research have been examined for ethical evaluation by the Ethical Committee (Committee for the Protection of the Persons CPP) Ile de France VI. They entail only negligible risks and constraints for the persons involved in the research.

26.3 Legal requirements (Role of the manager, CPP, CCTIRS, CNIL)

26.3.1 Role of the manager

The Assistance Publique des Hôpitaux de Paris (AP-HP) will be the manager of this research and be represented by the Delegation for Research and Innovation (DRCI).

The so-called manager will take the initiative of this research, will ensure its management and will check that the funding is provided. The manager will submit the protocol for approbation to the Committee for the Protection of Persons (CPP).

In accordance with amended law n°78-17 of January 6, 1978, the manager will send an opinion request to the Consultative Committee on data collection in Health Research (Comité consultatif sur le traitement de l'information en matière de recherche dans le domaine de la Santé (CCTIRS) and an authorization request to the National Data Protection Commission (Commission Nationale pour Informatique et Liberté (CNIL)) for the data processing during the research.

26.3.2 Submission to the CPP

This research obtained the favorable decision of the Comité de Protection des Personnes CPP Ile de France IV (Pitié-salpétrière) on 22/06/2016.

The opinion of the above-mentioned committee was notified in the information form to the persons concerned.

26.3.3 Notice from CCTIRS and authorization from the CNIL

This research is subject to the modified law n°78-17 of January 6, 1978 relating to data collection, files and freedom.

Consequently, the data collected in the context of multi-center research needs to be referred for advice to the Consultative Committee on data collection in Health Research (CCTIRS) and for authorization to the National Data Protection commission (CNIL).

For monocentric research, only a regular declaration to the CNIL is necessary.

Information on the persons rights who will participate in the research is included in the information form for the patient: right of access and rectification, right to oppose the transmission of data covered by professional secrecy that may be used in the context of this research.

26.3.4 Substantial modification to the protocol

The investigator or coordinator will inform the Delegation for Clinical research and Innovation (DRCI) of any intended changes to the protocol. Any substantial change will be submitted for advice to the CPP by the research manager.

26.3.5 Final research report or publication

The final research report or publication will be submitted for comments to every participating centers. The final version will be sent to the manager as soon as possible after the effective research end.

26.3.6 Data ownership

AP-HP is the data owner and no use or transmission to a third party will be authorized without prior consent.

26.3.7 Publication rules

You must mention the AP-HP in the affiliations of the author(s) of the publications resulting from your research and mention the AP-HP Manager (DRCI).

3. ***Mention of AP-HP affiliation for projects managed by PA-HP***
   - *If an author has several affiliations, the order in which the institutions (AP-HP, University, INSERM...) are cited is not important.*
   - *Each of these affiliations must be identified by an address separated by a semicolon (;).*
   - *The AP-HP institution must appear under the abbreviation "**AP-HP**" first in the address followed precisely by **AP-HP**, hospital, department, city, postal code, France*
4. ***Mention of the AP-HP manager (DRCI)** in **the acknowledgments of the** manuscript*
   - *"The sponsor was Assistance Publique - Hôpitaux de Paris (Department of Clinical Research and Development)".*

This search is registered on the http://clinicaltrials.gov/ website under the n°NCT02855320

27. REFERENCES

1.Saraux A, Combe B, Blin P et al, Survey of the therapeutic management of rheumatoid arthritis in France: the OPALE study. Clin Exp Rheumatol, 2010, 28(3):325-32. Epub 2010 Jun 23

2.Singh JA, Cameron C, Noorbaloochi S et al, Risk of serious infection in biological treatment of patients with rheumatoid arthritis: a systematic review and meta-analysis. Lancet, 2015 May 11. pii: S0140-6736(14)61704-9. doi: 10.1016/S0140-6736(14)61704-9. [Epub ahead of print]

3.Dixon WG, Symmons DP, Lunt M et al Serious infection following anti-tumor necrosis factor alpha therapy in patients with rheumatoid arthritis: lessons from interpreting data from observational studies. Arthritis Rheum. 2007 Sep;56(9):2896-904.

4.http//www.has.fr. Structuring a therapeutic patient education (TPE) program in the field of chronic diseases [Therapeutic patient education (TPE) Definition, goals, and organization]. Recommendations Haute Autorité de Santé 2007.

5.Gaujoux-Viala C, Gossec L, Cantagrel A et al, Recommandations of 81(4):287-97.the French Society of Rheumatology for managing rheumatoid arthritis. Joint Bone spine, 2014, 81(4):287-97.

6.Wendling D, Lukas C, Paccou et al, Recommendations of the French Society for Rheumatology (SFR) on the everyday management of patients with spondyloarthritis. Joint Bone Spine. 2014 Jan;81(1):6-14. doi: 10.1016/j.jbspin.2013.12.002. Epub 2014 Jan 10

7.Gossec L, Fautrel B, Flipon É, Lecoq d'André F, Marguerie L, Nataf H, Pallot Prades B, Piperno M, Poilverd RM, Rat AC, Sadji F, Sordet C, Thevenot C, Beauvais C. Safety of biologics: elaboration and validation of a questionnaire assessing patients' self-care safety skills: the BioSecure questionnaire. An initiative of the French Rheumatology Society Therapeutic Education section. Joint Bone Spine. 2013 Oct;80(5):471-6. doi: 10.1016/j.jbspin.2012.11.009. Epub 2013 Aug 20.

8.L. Pfeffer, A. Janoly-Dumenil, A. Donjon. Effects of an educational program on the safety knowledge and skills of patients with rheumatoid arthritis treated by biologics. Ann Rheum Dis 2013;72(Suppls3):150

9.Berard L , J.-F. Menard JF , J. Benichou J , T. Lequerre T , Vittecoq O , S. Pouplin S Effectiveness of collective educational intervention in the management of sub cutaneous biological agents in patients with inflammatory rheumatism. Ann Rheum Dis 2013;72(Suppls3):355

10.Orefice D, Beauvais C, Gossec L et al Cross-sectional study of self-care safety skills in 677 patients on biodrugs for inflammatory joint disease. Joint Bone Spine. 2014 Dec;81(6):502-7. doi: 10.1016/j.jbspin.2014.04.010. Epub 2014 Jun 23.

11.Rat AC, Fautrel B, Flipon E, Gossec L, Marguerie L, Nataf H, Pallot Prades B, Poilvert RM, Royant V, Sadji F, Sordet C, Thevenot C, Beauvais C. "Factors associated with knowledge and safety skills of arthritis patients receiving biologics: a survey of 677 patients". Soumis à publication à Rheumatology.

12.Beauvais C, Perdriger A. Diversity and territorial distribution of therapeutic education programs for osteoarticular diseases: a census of regional health agencies. Rev Rhum, 2012, 79, A301

14. 15. J N Burdine, M R Felix, A L Abel et al The SF-12 as a population health measure: an exploratory examination of potential for application. Health Serv Res. 2000 Oct; 35(4): 885-904.

16. Gossec L, Dougados M, Rincheval N et al. Elaboration of the preliminary Rheumatoid Arthritis Impact of Disease (RAID) score: a EULAR initiative. Ann Rheum Dis. 2009 Nov;68(11):1680-5. doi: 10.1136/ard.2008.100271. Epub 2008 Dec 3.

17. Nicassio PM, Wallston KA, Callahan LF, Herbert M, Pincus T: The measurement of helplessness in rheumatoid arthritis. The development of the arthritis helplessness index. J Rheumatol 1985, 12:462-467

18.L. Prevoo, M.A. van't Hof, H.H. Kuper, et al.Modified disease activity scores that include twenty-eight-joint counts. Development and validation in a prospective longitudinal study of patients with rheumatoid arthritis.Arthritis Rheum, 38 (1995), pp. 44-48

19. Fransen J, van Riel PL. The Disease Activity Score and the EULAR response criteria. Clin Exp Rheumatol. 2005 t;23(5 Suppl 39):S93-9

20. Machado P, Landewé R, Lie E et al Ankylosing Spondylitis Disease Activity Score (ASDAS): defining cut-off values for disease activity states and improvement scores. Assessment of SpondyloArthritis international Society. Ann Rheum Dis. 2011 Jan;70(1):47-53. doi: 10.1136/ard.2010.138594. Epub 2010 Nov 10

21.Garrett S, Jenkinson T, Kennedy LG et al A new approach to defining disease status in ankylosing spondylitis: the Bath Ankylosing Spondylitis Disease Activity Index. J Rheumatol. 1994 Dec;21(12):2286-91.

22. Lukas C, Landewé R, Sieper J et al Development of an ASAS-endorsed disease activity score (ASDAS) in patients with ankylosing spondylitis. Ann Rheum Dis. 2009 Jan;68:18-24. doi: 10.1136/ard.2008.094870. Epub 2008 Jul 14.

23. Aletaha D, Neogi T, Silman AJ et al, 2010 rheumatoid arthritis classification criteria: an American College of Rheumatology/European League Against Rheumatism collaborative initiative. Arthritis Rheum. 2010;62:2569–2581

24. Rudwaleit M, van der Heijde D, Landewé R et al, The development of Assessment of SpondyloArthritis international Society classification criteria for axial spondyloarthritis (part II): validation and final selection. Ann Rheum Dis. 2009 Jun;68(6):777-83. doi: 10.1136/ard.2009.108233. Epub 2009 Mar 17

25. Rudwaleit M1, van der Heijde D, Landewé R et al The Assessment of SpondyloArthritis International Society classification criteria for peripheral spondyloarthritis and for spondyloarthritis in general. Ann Rheum Dis. 2011 Jan;70(1):25-31. doi: 10.1136/ard.2010.133645. Epub 2010 Nov 24.

26. Neame R, Hammond A. Beliefs about medications: a questionnaire survey of people with rheumatoid arthritis. Rheumatology 2005 ;44(6):762-7. Epub 2005 Mar 1.

28. APPENDICES

28.1 Appendix 1: List of participating centers in June 12, 2017

N°	DEPARTMENT	FULL NAME AND ADDRESS	INVESTIGATORS	CONTACT DETAILS
**001**	Rheumatology	CHU Saint Antoine, 184, rue Faubourg Saint Antoine, 75012 Paris, France	Catherine Beauvais	Tel: 01 49 28 25 20 Email: catherine.beauvais@aphp.fr
**002**	Rheumatology	CHU Pitié Salpétrière, 47-83 Boulevard de l'Hôpital, 75013 Paris.	Laure Gossec	Tel: 01 42 16 00 00 Email: laure.gossec@aphp.fr
**003**	Rheumatology	CHU Clermont Ferrand Hospital Gabriel Montpied 58 rue Montalembert. 63003 Clermont-Ferrand.	Martin Soubrier (IP) Françoise Fayet	Tel: 04 73 75 07 50 Email: ideetp@chu-clermontferrand.fr msoubrier@chu-clermontferrand.fr
**004**	Rheumatology	CHU Grenoble Hospital South, Avenue de Kimberley CS 90338 - 38434 CHIPS	Laurent Grange	Tel: 04 76 76 75 75 Email: LGrange@chu-grenoble.fr
**005**	Rheumatology	CHU Brabois, rue du Morvan 54511 VANDOEUVRE LES NANCY.	Anne Christine Rat	Tel: 03 83 85 11 88 Email: ac.rat@chu-nancy.fr
**006**	Rheumatology	CHU Rouen, 1 rue de Germont 76031 Rouen, France	Sophie Pouplin	Tel: 02 32 88 89 90 Email: Sophie.Pouplin@chu-rouen.fr
**007**	Rheumatology	CHU Nantes Hôtel Dieu, Place Alexis Ricordeau 44093 Nantes cedex 1	Yves Maugars (IP)	Tel: 02.40.08.33.33 Email: yves.maugars@chu-nantes.fr

**008**	Rheumatology	University Hospitals Strasbourg, 1 Av Moliere 67098 Strasbourg Cedex, France	Christelle Sordet	Tel: 03 88 11 67 68<br>Email: Christelle.SORDET@chru-strasbourg.fr
**009**	Rheumatology	CHRU Pontchaillou, 2 rue Henri Le Guilloux 35033 RENNES	Aleth Perdriger	Tel : 02 99 28 43 21<br>Email: aleth.perdriger@chu-rennes.fr
**010**	Rheumatology	CHU Saint Etienne Hospital of Bellevue	Béatrice Pallot Prades	Email: beatrice.pallotprades@chu-st-etienne.fr
**011**	Rheumatology	CHRU Trousseau 37044 Cedex Towers 9	Isabelle Griffoul-Espitalier	Tel: 02 47 47 59 17<br>Email: i.griffoul@chu-tours.fr

28.2 Appendix 2: Questionnaires

28.2.1 BioSecure Questionnaire

This questionnaire contains important information about your knowledge of the biologic treatment you take for your arthritis. Please answer the following questions, even if you feel they do not concern you. For each question, please tick the answer which best corresponds to what you think or feel Thank you

Your name: ……………………………………………………………………

Today's date: …………………………………………….

Questions.

1. What is your current biologic treatment? *Please tick one answer only*

- ☐ Enbrel (etanercept)
- ☐ Humira (adalimumab)
- ☐ Remicade (infliximab)
- ☐ Mabthera (rituximab)
- ☐ Orencia (abatacept)
- ☐ RoActemra or Actemra (tocilizumab)
- ☐ Cimzia (certolizumab)
- ☐ Other ……………………………
- ☐ I don't know

2. I can stop my biologic treatment if my arthritis is completely under control (in remission). *Please tick one answer only.*

Yes □ No □ I don't know □

3. Infections are more common during biologic treatment. *Please tick one answer only.*

Yes □ No □ I don't know □

4. Among the following situations, which ones require special precautions or a change of your biologic treatment? *Please tick one answer for each situation.*

	Yes	No	I don't know
4.1. Drinking milk	□	□	□
4.2. Foreign travel	□	□	□
4.3. Having an operation	□	□	□
4.4. Running	□	□	□
4.5. Having a tooth extraction	□	□	□
4.6. Drinking a glass of wine	□	□	□
4.7. Eating organic food	□	□	□
4.8. Planning having a baby	□	□	□

5. Who do I need to tell about my biologic treatment? *Please tick one answer for each person.*

	Yes	No	I don't know
5.1. My doctor (general practitioner)	□	□	□
5.2. My employer	□	□	□
5.3. My dentist	□	□	□
5.4. The anesthetist in case of surgery	□	□	□
5.5. My fitness instructor or sports coach	□	□	□
5.6. The lifeguard at the swimming pool	□	□	□
5.7. My bank manager.	□	□	□

6. When taking a biologic, all vaccinations should be avoided. *Please tick one answer only.*

true □ *false*□ *I don't know*□

7. When using biologics, a woman must use effective contraception. *Please tick one answer only.*

true □ *false*□ *I don't know*□

8. Which of the following situations lead to special precautions or to modifications in the management of biologic treatment? *Please tick the right answer for each situation.*

	Yes	No	I don't know
8.1. High temperature / fever	□	□	□
8.2. Frequent need of urinating	□	□	□
8.3. A sprained ankle	□	□	□
8.4. A cough	□	□	□
8.5. Out of breath for no apparent reason	□	□	□
8.6. Constipation	□	□	□
8.7. Have a burning sensation while urinating	□	□	□
8.8. A weight gain of 3 kilograms (6 pounds)	□	□	□
8.9. A weight loss of 1 kilogram (2 pounds)	□	□	□

Clinical situations

Biologic treatment may be given by sub-cutaneous injections at home or infusions at hospital. The following situations refer to both administration type. Please answer the questions by referring to your own situation.

Case no.1

Cathy is receiving biologic treatment for rheumatoid arthritis. During the Christmas holidays, her husband and daughter have fever with a temperature of 38°5 C (101 degrees Fahrenheit), they have a cough and a runny nose. Their doctor gave them some treatment but said they did not need antibiotics since it was only a viral infection. One week later, Cathy develops the same symptoms: fever, a cough and a runny nose. Which of Cathy's decisions (below) do you agree with? *(several possibilities)*

		Yes	No	I don't know
1	Cathy takes the treatment which was prescribed for her husband, since it was effective for him.	□	□	□
2	She waits a few days before contacting the doctor because her husband and daughter recovered in a few days.	□	□	□
3	She has her biologic treatment (injection or infusion) because it's only a virus.	□	□	□

Case no. 2

Paul's arthritis is treated with biologics. The day before his biologic treatment (injection or infusion) he has bronchitis with a barking cough and a temperature of 38°5 C (101 degrees Farhenheit). His doctor prescribes antibiotics which Paul starts the same evening. Paul decides he should not have his biologic treatment (not perform the injection or tell the infusion centre not to perform the infusion). Which of the following statements do you agree with? *(several possibilities)*

		True	False	I don't know
1	Paul was right not to take his biologic treatment.			
2	Paul was right to start antibiotics as soon as possible.			
3	If Paul has bronchitis again, he will know which antibiotics he can take in case his doctor is not available.			
4	Paul can have his biologic treatment tomorrow if he starts the antibiotics today.			
5	Paul was right to call his doctor.			

Case no. 3

This summer, Christine went on holiday with her family. During her stay, she rested a lot and her arthritis was much better. She only needed anti-inflammatory drugs for a few days and she decided not to carry on with her biologic

therapy (injection or infusion). When she came back, one of her friends asks her "Why should you start your biologic again, as you no longer have pain?"

Which of Christine's answers (below) do you agree with? *(several possibilities)*

		Yes	No	I don't know
1	I will start my biologic again.	□	□	□
2	If my arthritis has not been painful for 3 weeks, it probably means that it is cured.	□	□	□

Case no. 4

In October, Jane, aged 53, learns that the flu vaccine is now available. She has been treated by biologics for 6 months. She is not sure whether or not she should have the flu vaccine. With which of Jane's opinions (below) do you agree with? *(several possibilities)*

		Yes	No	I don't know
1	I will get the flu vaccine.	□	□	□
2	I am more likely to have a reaction to the flu jab due to my biologic treatment.	□	□	□
3	I have to avoid the flu jab because of my biologic treatment.	□	□	□
4	I will talk about it with my doctor.	□	□	□

Case no. 5

Bill is treated by biologics for arthritis; he likes gardening. Bill has cut his index finger while planting a rosebush. Which of the following statements (below) do you agree with? *(several possibilities)*

		Yes	No	I don't know
1	The wound needs to be cleansed and dressed straight away.	□	□	□
2	The wound is more likely to go septic because of the biologic therapy.	□	□	□
3	Bill must take antibiotics straight away.	□	□	□
4	Bill can have the tetanus vaccine, even though he is treated with a biologic.	□	□	□

Case no.6

Sarah is treated with biologics. She has an appointment with a surgeon to plan cataract surgery. The surgeon proposes to operate in 10 days.

Which of the following statements (below) do you agree with? *(several possibilities)*

		Yes	No	I don't know
1	The surgery should definitely be avoided.	□	□	□
2	Sarah agrees with the scheduled date for the operation, the sooner the better.	□	□	□
3	Sarah refuses the scheduled date because she needs to think about stopping her biologic therapy first.	□	□	□
4	Sarah informs the surgeon about her biologic therapy.	□	□	□
5	Sarah informs the anesthetist about her biologic therapy.	□	□	□

If you have subcutaneous biologic therapy, please answer the following questions:

Question 9

Please tick one answer only.

The biologic treatment must be stored:

- in the refrigerator □
- in the freezer □
- at room temperature □
- I don't know □

Case no.7

Alice is about to do her biologics sub cutaneous injection. Which of Alice's actions was incorrect? *Tick one only.*

- □ She gets the biologic out and waits a little before injection
- □ She disinfects the skin
- □ She injects herself in the abdomen or the thigh
- □ She puts the used syringe and needle in the wastebin.

2. In your opinion, is there anything Alice has forgotten to do? *(Only one sentence)*

Thank you for filling in this questionnaire.

28.2.2 SF 12

The following questions ask for your views about your health. Your answers will help monitor the condition of your health and to know how well you are able to do your usual activities. Answer all of the following questions by following the instructions you have been given. If you are unsure, please give the best answer you can.

1.In general, would you say your health is:
(mark one answer only)

- Excellent ....❑
- Very good...❑
- Good...❑
- Fair ....❑
- Poor ....❑

2.The following is a list of activities you might do during a typical day. For each of these, indicate whether your health now limits you in these activities. (mark one answer only per line).

	Limited a lot	Limited a little	Not limited at all
A.Moderate physical activities such as moving a table, pushing a vacuum cleaner, bowling	❑	❑	❑
B.Climbing several flights of stairs	❑	❑	❑

3.During the past 4 weeks, due to your physical condition, (mark one answer only per line)

	All the time	Most of the time	Some of the time	A little of the time	None of the time
A.Have you accomplished less than you would like?	❑	❑	❑	❑	❑
B.Have you had to stop doing certain things?	❑	❑	❑	❑	❑

4.During the past 4 weeks, due to your emotional state (such as feeling sad, nervous or depressed) (mark one answer only per line)

	All the time	Most of the time	Some of the time	A little of the time	None of the time
A.Have you accomplished less than you would like?	❑	❑	❑	❑	❑
B.have you had difficulties in doing what you had to do with as much care and attention as usual?	❑	❑	❑	❑	❑

5.During the past 4 weeks, how much did your physical pain interfere with your work or housework? (mark one answer only)

- Not at all ....❑
- A little bit...❑

Beauvais C, *et al. RMD Open* 2022; 8:e001828. doi: 10.1136/rmdopen-2021-001828

- Moderately ....❑
- Quite a bit ....❑
- Extremely ....❑

6.The following questions are related to how you have felt over the past 4 weeks. For each question, please indicate the response you feel is most appropriate.
During the past 4 weeks, have there been times when: (mark one answer only per line)

	All the time	Most of the time	Some of the time	A little of the time	None of the time
A.Have you felt calm & peaceful?	❑	❑	❑	❑	❑
B.Have you felt full of energy?	❑	❑	❑	❑	❑
C.Have you felt down-hearted and blue?	❑	❑	❑	❑	❑

7.During the past 4 weeks, how much of the time has your physical health or emotional problems interfered with your social activities and relationships with others (family, friends, etc.)? (mark one answer only)

- All the time...❑
- Most of the time ....❑
- From time to time ....❑
- Rarely ....❑
-Never ……………………………………….. ❑

28.2.3 BMQ

I would like to ask you about your personal views about medicines prescribed for your arthritis. These are statements other people have made about their arthritis medication. Please indicate the extent to which you agree or disagree with them by placing a cross in the appropriate box. Please only cross one box per question.

	Strongly agree	Agree	Uncertain	Disagree	Strongly disagree
My health, at present, depends on my medicines					
My life would be impossible without my medicines					
Without my medicines I would become very ill					
My health in the future will depend on my medicines					
My medicines protect me from becoming worse					
Having to take medicines worries me					

I sometimes worry about the long-term effects of my medicines					
My medicines are a mystery to me					
My medicines disrupt my life					
I sometimes worry about becoming too dependent on my medicines					

28.2.4 RAID: Rheumatoid Arthritis Impact of Disease

3. Pain
Circle the number that best describes the pain you felt due to your rheumatoid arthritis during the last week:

None 0 1 2 3 4 5 6 7 8 9 10 Extreme

4. Functional disability assessment
Circle the number that best describes the difficulty you had in doing daily physical activities due to your rheumatoid arthritis during the last week.

No difficulty 0 1 2 3 4 5 6 7 8 9 10 Extreme difficulty

3. Fatigue

Circle the number that best describes how much fatigue you felt due to your rheumatoid arthritis during the last week.

No fatigue 0 1 2 3 4 5 6 7 8 9 10 Totally exhausted

4. Sleep
Circle the number that best describes the sleep difficulties (i.e., resting at night) you felt due to your rheumatoid arthritis during the last week.

No difficulty 0 1 2 3 4 5 6 7 8 9 10 Extreme difficulty

5. Physical well-being

Considering your arthritis overall, how would you rate your level of physical well-being during the past week? Circle the number that best describes your level of physical well-being.

Very good	0	1	2	3	4	5	6	7	8	9	10	Very bad

6. Emotional well-being

Considering your arthritis overall, how would you rate your level of emotional well-being during the past week?

Circle the number that best describes your level of emotional well-being.

Very good	0	1	2	3	4	5	6	7	8	9	10	Very bad

7. Coping

Considering your arthritis overall, how well did you cope (manage, deal, make do) with your disease during the last week?

Very well	0	1	2	3	4	5	6	7	8	9	10	Very poorly

28.2.5 Arthritis helplessness index

	Strongly disagree	Disagree	Agree	Strongly agree
My condition is controlling my life	❑	❑	❑	❑
No matter what I do, or no matter hard I try, I just can't seem to get relief from my pain.	❑	❑	❑	❑
I am coping effectively with my condition	❑	❑	❑	❑
I would feel helpless if I couldn't rely on other people for help with my condition	❑	❑	❑	❑
It seems as though fate and other factors beyond my control affect my condition	❑	❑	❑	❑

28.2.6 Disease activity score DAS 28

Number of tender joints NAD (0-28)
Number of swollen joints NAG (0-28)
Patient global assessment 0-10 (question1)
Erythrocyte sedimentation rate (mm)
C reactive protein) mg/ml

Question1 : Considering all the ways your arthritis has affected you, how active do you feel your arthritis is … ?

28.2.7 BASDAI Bath Ankylosing Spondylitis Disease Activity Index

Please place a mark on each line below to indicate your answer to each question relating to the past week

1.How would you describe the overall level of fatigue/tiredness you have experienced?

None 0 1 2 3 4 5 6 7 8 9 10 Very severe

2.How would you describe the overall level of AS neck, back or hip pain you have had?

None 0 1 2 3 4 5 6 7 8 9 10 Very severe

3.How would you describe the overall level of pain/swelling in joints other than neck, back or hips you have had?”

None 0 1 2 3 4 5 6 7 8 9 10 Very severe

4.How would you describe the overall level of discomfort you have had from any areas tender to touch or pressure?

None 0 1 2 3 4 5 6 7 8 9 10 Very severe

5.How would you describe the overall level of morning stiffness you have had from the time you wake up?

None 0 1 2 3 4 5 6 7 8 9 10 Very severe

6.How long does your morning stiffness last from the time you wake up?

0 hrs---------------------½----------------------1--------------------½----------------2 or more

28.2.8 ASDAS

Please place a mark on each line below to indicate your answer to each question relating to the past week
1.How would you describe the overall level of AS neck, back or hip pain you have had?

None	0	1	2	3	4	5	6	7	8	9	10	Very severe

2.How long does your morning stiffness last from the time you wake up?
1 hrs---------------------½----------------------1--------------------½----------------2 or more

3.How would you describe the overall level of pain/swelling in joints other than neck, back or hips you have had?"

None	0	1	2	3	4	5	6	7	8	9	10	Very severe

4. How active was your spondylitis on average during the last week?

Not at all	0	1	2	3	4	5	6	7	8	9	10	Very much
