# Supplementary material for: Efficacy of a nurse-led patient education intervention in promoting safety skills of patients with inflammatory arthritis treated with biologics: a multicentre randomised clinical trial
Source: RMD Open. 2022 Mar 16;8(1):e001828. doi: 10.1136/rmdopen-2021-001828 (PMC8928395; doi:10.1136/rmdopen-2021-001828)
Supplement: Supplementary data [file rmdopen-2021-001828supp003.pdf]

Supplementary Material 3 : Statistical analysis plan

1 FINAL STATISTICAL ANLYSIS PLAN.

1.1 Description of the statistical methods.

The analysis will be performed with SAS V9.3 software.
No intermediate analysis will be planned. The analysis will be performed after the database freezing.
The analysis will be conducted in intention to treat population.
The patients baseline (BL) characteristics will be described in total and by randomization group.
The education self-assessment collected by the nurse in the education booklet will be described at BL and at 3 months.
Qualitative data will be described by frequencies and numbers, and quantitative variables will be described by their means and standard deviations, ranges or medians and interquartile ranges. The number of patients who will switch from a SC biologic to a IV biologic treatment after BL will be described in total and by randomization group.

The analysis will be conducted in intention to treat population. The primary outcome will then be analyzed in per protocol population.

The tests will be performed at the 5% threshold except in the case of multiple tests for which a Bonferroni correction will be applied.

1.1.1 Analysis of the primary outcome:

The primary outcome will be the patient's BioSecure score at 6 months. The BioSafe questionnaire consists of 24 competencies assessed in two parts, one part common to patients with intravenous (IV) and subcutaneous (SC) bDMARDs and the second part only for patients with SC bDMARD.
Part 1, includes 8 questions and 6 case scenarios.
Part 2 includes 1 question and 1 additional case.
Each correct answer is associated with 1 point. A missing data will be considered a false response.
The competency score is calculated as the sum of the points obtained, reported to 100.

The mean value of the BioSecure score at 6 months (with distinction between SC and IV patients) will be compared by a Student t-test or a non-parametric test if necessary.

Sensitivity analysis

- Primary outcome analysis in the intention treat population on the available data at 6 months.
- Primary outcome analysis in the per protocol population.

1.1.2. Analysis of secondary outcomes

- The criteria will be quality of life assessed by the SF12 questionnaire at BL and 6 months and

- The MOS-SF-12 questionnaire is derived from the SF36 questionnaire.

It includes 12 items divided into eight dimensions (physical function, physical limitation, physical pain, emotional limitation, mental health, vitality, social functioning, overall perceived health).
The Mental Quality of Life (MQL) score and the Physical Quality of Life (PQL) score are then calculated only if all 12 items have been completed. Scores range from 0 to 100.
The scores at BL and 6 months will be described by groups.
Each score differences between BL and 6 months will be calculated and compared between groups, by a linear regression taking into account the BL score value. If the distribution of the difference does not follow a normal distribution, the percentage of variation will be calculated and compared between groups by a non-parametric Wilcoxon-Man Whitney test.

- Severe infections, defined as infections requiring hospitalization and/or intravenous antibiotics.

Treatment-related severe infections rate occurring within 6 months after initiating bDMARDS will be described and compared between groups using an accurate Fisher's test.

- Coping with the disease and psychological well-being will be assessed by a NRS. The obtained score will therefore be an integer between 0 and 10. The Arthritis Helplessness index (AHI) will be assessed at BL and 6 months, score integer between 5 and 20.

Scores will be described at BL and 6 months.
The difference between BL and 6 months will be calculated and compared between groups by a linear regression with account for the initial value. If difference distribution does not follow a normal distribution, the percentage of change will be calculated and compared between groups by a nonparametric Wilcoxon-Man Whitney test.

- Opinions about treatment will be evaluated by the BMQ (Beliefs about medicine questionnaire). It includes 10 items divided into 2 dimensions : necessity scale and concerns scale. The scores are integer between 5 and 25. Scores will be described at BL and 6 months. The difference between BL and 6 months will be calculated and compared between groups by a Student t-test or a non-parametric test if necessary.
- Disease activity will be evaluated by the DAS, ASDAS and BASDAI activity scores. The difference between BL and 6 months will be calculated and compared between groups by a Student t-test or a non-parametric test if necessary.

1.1.3. Exploratory analysis.

The value of the BioSecure score will be described according to: sex, age, education level, disease duration and underlying disease (Rheumatoid Arthritis or Axial Spondyloarthritis or peripherical Spondyloarthritis including psoriatic arthritis)). The score values will be compared by a t-test or a non-parametric test for the qualitative variables. The correlation between scores and age will be assessed either by calculating a Pearson or by Spearman correlation coefficient if applicable. A coding of age into classes will be considered.

These last two analyses will be performed on the population per protocol according to available data.

To evaluate whether the effect of the intervention on the BioSecure score at 6 months varies according to the patient's BL level of information on treatments (≤7 vs > 7), the interaction between these 2 variables will be tested by a linear regression model.

1.2 Consideration of missing data.

If the BioSecure score cannot be calculated (due to not collected questionnaires or questionnaire missing data), the patient will be considered a failure regardless of the randomization group. The score will be replaced by the 25 percentile value, calculated on the total population.

1.3 Population for analysis.

The ITT (Intention To Treat) population will consist of all randomized patients with indication to a first SC biologic treatment who consented to the research.

The per-protocol population will consist of randomized patients who have fully participated in the study, with no protocol deviation.
Deviation to the protocol will be

- non-compliance with selection criteria
- Primary outcome not available (total BioSecure score)
- Non-compliance with the treatment allocated by the random assignment (at least one education session is required for patients in the intervention group)
- Non- compliance with the time limit between BL and the 6 month visit (visit performed less than 4 months or more than 9 months after inclusion).

2 MAJOR CHANGES TO THE ORIGINAL ANALYSIS

- BioSecure score at 6 months

- Each correct answer is associated with 1 point. A missing data will be considered a false response.
  The skill score is calculated as the sum of the points obtained. It is then multiplied by 1.82 to relate to base 100.
  If the BIOSECURE score is missing (no items filled in), the value of the score is imputed by the value of the 25th percentile, calculated on the ITTm population with available data.

- No patients were switched from SC to IV biotherapy after BL, so analysis of the rate of patients on IV biotherapy was not performed.

- The questions of the BioSecure questionnaire were described. The proportion of correct answers to each question was described for each group. The difference between the 2 proportions was presented with its 95% confidence interval was calculated with its continuity-corrected Wald 95% confidence interval (CI).

- The safety skills in the BioSecure questionnaire were gathered by key sub-scores related to 6 domains: infections, dental care and surgery, vaccinations, child conception, adherence-related behaviors and drug storage/cold chain preservation. The difference in the proportion of good responses in the 6 key domains of the BioSecure questionnaire was calculated with its continuity-corrected Wald 95% confidence interval (CI).

- Quality of life.

The SF12-v2 summary scores are calculated according to the scoring method proposed by QualyMetric.
Standardization of subscales was performed using means and standard deviations from the 1998 US general population. Given the small number of questions asked in the SF12 questionnaire, when one or more items were missing, scores were not replaced.

The questionnaire used to assess quality of life was the SF12-v2. However, one of the response modalities to question 2 (2a and 2b) was modified by error ("Uncertain" instead of "Not limited at all). Therefore, a corrective questionnaire was implemented during the study. For patients who completed the incorrect questionnaire at BL and 6 months, the response to question 2 was considered unavailable. Consequently, the SF12 scores were not calculated for these patients.

- Severe infections within 6 months (1=Yes/0=No)

The item is score 1 if :

- the patient was hospitalized (in any department) for a severe infection between BL and 6 months.
- the patient received intravenous antibiotic therapy without being hospitalized for a severe infection between BL and 6 months.

- AHI score

The AHI subscale consists of 5 items. Responses to the items are assessed by a 4-point Likert scale (1 = strongly disagree, 2 = disagree, 3 = agree, 4 = strongly agree).
For question 3, the response scale is reversed.
The total score is calculated as the sum of the scores obtained for the 5 items.
When one or more items were missing, the score was not replaced.

- BMQ

The BMQ questionnaire consists of 10 items divided into 2 dimensions: the necessity scale and the concerns scale. Responses to the items are evaluated by a 5-point Likert scale (1 = strongly agree, 2 = agree, 3 = not sure, 4 = disagree, 5 = strongly disagree).
The necessity scale is calculated as the sum of the scores obtained for the first 5 items (questions 1 to 5).
The concern scale is calculated as the sum of the scores obtained on the last 5 items (questions 6 to 10).
Scores range from 5 to 25.
When one or more items were missing, scores were not replaced.

Beauvais C, *et al. RMD Open* 2022; 8:e001828. doi: 10.1136/rmdopen-2021-001828
