# Supplementary material for: Efficacy of a nurse-led patient education intervention in promoting safety skills of patients with inflammatory arthritis treated with biologics: a multicentre randomised clinical trial
Source: RMD Open. 2022 Mar 16;8(1):e001828. doi: 10.1136/rmdopen-2021-001828 (PMC8928395; doi:10.1136/rmdopen-2021-001828)
Supplement: Supplementary data [file rmdopen-2021-001828supp002.pdf]

Supplementary material 4 : additional results

eAppendix 1. Cormorbidities
eAppendix 2. Proportion of correct answers to the relevant safety items of the Biosecure questionnaire at 6 months
eAppendix 3. Nurse self-assessment of intervention

eAppendix 1. Cormorbidities [a]

Variable	Control group (n=63)		Intervention group (n=64)	
	N [b]	No. (%)	n[b]	No. (%)
Arthritis (other than IA)	59	11 (18.6)	64	16 (25.0)
Osteoporosis	60	4 (6.7)	63	2 (3.2)
Asthma	60	7 (11.7)	62	2 (3.2)
Chronic obstructive pulmonary disease, acquired respiratory distress syndrome, or emphysema	60	1 (1.7)	63	2 (3.2)
Angina	60	1 (1.7)	63	0 (0)
Congestive heart failure (or heart disease)	60	2 (3.3)	63	1 (1.6)
Heart attack (myocardial infarct)	60	0 (0)	64	2 (3.1)
Neurological disease (such as multiple sclerosis or Parkinson's)	60	0 (0)	63	0 (0)
Stroke or transischemic attack	60	0 (0)	63	1 (1.6)
Peripheral vascular disease	60	0 (0)	63	1 (1.6)
Diabetes types I and II	61	3 (4.9)	63	0 (0)
Upper gastrointestinal disease (ulcer, hernia, reflux)	60	7 (11.7)	63	3 (4.8)
Depression	60	4 (6.7)	63	6 (9.5)
Anxiety or panic disorder	58	9 (15.5)	63	12 (19.0)
Visual impairment (such as cataracts, glaucoma, macular degeneration)	60	8 (13.3)	63	4 (6.3)
Hearing impairment (very hard of hearing, even with hearing aids)	60	2 (3.3)	63	3 (4.8)
Degenerative disc disease (back disease, spinal stenosis, or severe chronic back pain)	60	15 (25.0)	64	20 (31.3)
Number of comorbidities (0-17)	57		61	
0		20 (35.1)		28 (45.9)
1		18 (31.6)		18 (29.5)
2		13 (22.8)		6 (9.8)
≥3		6 (10.5)		9 (14.8)
Number of comorbidities (0-17), median [IQR]	57	1.0 [0.0–2.0]	61	1.0 [0.0–1.0]

[a] from the Groll Score (Groll DL, To T, Bombardier C, Wright JG. The development of a comorbidity index with physical function as the outcome. J Clin Epidemiol 2005;58:595-602.). [b]Number of available data
Abbreviation: IQR, interquartile range

eAppendix 2. Proportion of correct answers to the relevant safety items of the Biosecure questionnaire at 6 months

Questions	Control group (n=63), No. (%)	Intervention group (n=64), No. (%)	Absolute difference IG-CG (95% CI)	Corresponding sub-scores
	**n [a] =59**	**n [a] =59**		
1. What is your current biologic treatment?	58 (98.3)	54 (91.5)	-6.8 (-16.3 to 2.7)	GK [b]
2. I can stop my biologic treatment if my arthritis is completely under control (in remission).	27 (45.8)	45 (76.3)	30.5 (12.1 to 48.9)	Adherence
3. Infections are more common during biologic treatment.	38 (64.4)	43 (72.9)	8.5 (-9.9 to 26.8)	Infection
4. Among the following situations, which ones require special precautions or a change of your biologic treatment?				
4.2. Foreign travel	37 (62.7)	44 (74.6)	11.9 (-6.4 to 30.2)	Storage
4.3. Having an operation	49 (83.1)	55 (93.2)	10.2 (-3.0 to 23.4)	Surgery
4.5. Having a tooth extraction	49 (83.1)	54 (91.5)	8.5 (-5.1 to 22.1)	Dental care
4.8. Planning a baby	33 (55.9)	38 (64.4)	8.5 (-10.8 to 27.8)	Conception
5. Who do I need to tell about my biologic treatment?				
5.1. My doctor (general practitioner)	58 (98.3)	59 (100)	1.7 (-3.3 to 6.7)	GK [b]
5.3. My dentist	55 (93.2)	56 (94.9)	1.7 (-8.5 to 11.9)	Dental care
5.4. The anesthetist in case of surgery	56 (94.9)	58 (98.3)	3.4 (-4.8 to 11.6)	Surgery
6. When taking a biologic, all vaccinations should be avoided.	48 (81.4)	51 (86.4)	5.1 (-9.8 to 20.0)	Vaccines
7. When using biologics, a woman must use effective contraception.	22 (37.3)	24 (40.7)	3.4 (-15.9 to 22.7)	Conception
8. Which of the following situations lead to special precautions or to modifications in the management of biologic treatment?				
8.1. High temperature/fever	55 (93.2)	58 (98.3)	5.1 (-3.8 to 14.0)	Infection
8.2. Frequent need to urinate	26 (44.1)	33 (55.9)	11.9 (-7.7 to 31.5)	Infection
8.4. A cough	44 (74.6)	49 (83.1)	8.5 (-7.9 to 24.8)	Infection
8.5. Out of breath for no apparent reason	35 (59.3)	39 (66.1)	6.8 (-12.3 to 25.9)	Infection
8.7. Have a burning sensation while urinating	38 (64.4)	49 (83.1)	18.6 (1.4 to 35.9)	Infection
9. Case no. 1. Fever. Cathy				
9.1 Cathy takes the treatment prescribed for her husband, since it was effective for him.	54 (91.5)	55 (93.2)	1.7 (-9.6 to 13.0)	Infection
9.2 She waits a few days before contacting the doctor because her husband and daughter recovered in a few days.	42 (71.2)	43 (72.9)	1.7 (-16.2 to 19.6)	Infection
9.3 She has her biologic treatment (injection or infusion) because it's only a virus.	39 (66.1)	49 (83.1)	16.9 (-0.2 to 34.1)	Infection
10. Case no. 2.Bronchitis. Paul				
10.1 Paul was right not to take his biologic treatment.	51 (86.4)	55 (93.2)	6.8 (-5.8 to 19.3)	Infection
10.2 Paul was right to start antibiotics as soon as possible.	52 (88.1)	54 (91.5)	3.4 (-9.2 to 16.0)	Infection
10.3 If Paul has bronchitis again, he will know which antibiotics he can take in case his doctor is not available.	46 (78.0)	54 (91.5)	13.6 (-0.9 to 28.0)	Infection
10.4 Paul can have his biologic treatment tomorrow if he starts the antibiotics today	44 (74.6)	47 (79.7)	5.1 (-11.7 to 21.9)	Infection
10.5 Paul was right to call his doctor	59 (100)	56 (94.9)	-5.1 (-12.4 to 2.2)	Infection
11. Case no. 3. Stopping biologics. Christine				
11.1 I will start my biologic again.	44 (74.6)	50 (84.7)	10.2 (-5.9 to 26.3)	Adherence

11.2 If my arthritis has not been painful for 3 weeks, it probably means that it is cured.	53 (89.8)	52 (88.1)	-1.7 (-14.7 to 11.3)	Adherence
12. Case no. 4. Flu vaccination.				
12.1 I will get the flu vaccine.	40 (67.8)	54 (91.5)	23.7 (8.2 to 39.3)	Vaccines
12.2 I am more likely to have a reaction to the flu jab due to my biologic treatment.	34 (57.6)	36 (61.0)	3.4 (-16.0 to 22.8)	Vaccines
12.3 I have to avoid the flu vaccine because of my biologic treatment.	49 (83.1)	50 (84.7)	1.7 (-13.3 to 16.6)	Vaccines
13. Case no. 5. Wounds.				
13.1 The wound needs to be cleansed and dressed straight away	59 (100)	59 (100)	.	Infection
13.2 The wound is more likely to go septic because of the biologic therapy.	46 (78.0)	50 (84.7)	6.8 (-8.9 to 22.5)	Infection
13.3 Bill must take antibiotics straight away.	36 (61.0)	40 (67.8)	6.8 (-12.1 to 25.7)	Infection
13.4 Bill can have the tetanus vaccine, even though he is treated with a biologic.	30 (50.8)	42 (71.2)	20.3 (1.4 to 39.2)	Vaccines
14. Case no. 6				Surgery
14. 1 The surgery should definitely be avoided.	40 (67.8)	50 (84.7)	16.9 (0.2 to 33.7)	Surgery
14.2 Sarah agrees with the scheduled date for the operation, the sooner the better.	32 (54.2)	40 (67.8)	13.6 (-5.6 to 32.7)	Surgery
14.3 Sarah refuses the scheduled date because she needs to think about stopping her biologic therapy first.	44 (74.6)	48 (81.4)	6.8 (-9.8 to 23.4)	Surgery
14.4 Sarah informs the surgeon about her biologic therapy.	59 (100)	56 (94.9)	-5.1 (-12.4 to 2.2)	Surgery
14.5 Sarah informs the anesthetist about her biologic therapy.	58 (98.3)	56 (94.9)	-3.4 (-11.6 to 4.8)	Surgery
15. Conservation.	56 (94.9)	57 (96.6)	1.7 (-7.3 to 10.7)	Storage
16. Case no. 7				Infection
16.1. Aseptia	52 (88.1)	56 (94.9)	6.8 (-4.9 to 18.4)	Infection

[a] Number of available data. [b] GK: general knowledge (not attributed in sub scores).
Responses to the last question of the Biosecure questionnaire are not presented because of a too small number of responses.
Sub-scores: adherence-related sub-score includes questions 2;11.1;11.1. Infections sub-score includes questions 3; 8.1;8.2;8.4;8.5;8.7;9.1-9.3;10.1-10.5;13.1-13.3;16.1. Vaccination sub-score includes questions 6;12.2;12.3;13.4. Surgery & dental care sub-score includes questions 4.3-4.5;5.3;5.4;14.1-14.5. child conception includes questions 4.8;7. Storage & cold chain maintenance sub-score includes questions 4.2;15.
The decoy not relevant questions 4.1;4.4;4.6;4.7;5.2;5.5;5.6;8.3;8.6;8.8;8.9 are not presented.
Abbreviations: IG, intervention group; CG, control group

eAppendix 3. Nurse self-assessment of intervention

	**Baseline (n=64)**				**At 3 months (n=59)**			
	**No. [a]**	**No. (%)**			**No. [a]**	**No. (%)**		
Interview with the nurse	64				54			
Free-style		31 (48.4)				24 (44.4)		
Semi-structured		33 (51.6)				30 (55.6)		
Discussion on expectations, fears, motivations fully carried out	64	64 (100)			54	53 (98.1)		
Education session fully carried out	63	62 (98.4)			54	53 (98.1)		
Education session: skills		**Discussed**		**Acquired**		**Discussed**		**Acquired**
Name the bDMARD	64	63 (98.4)	63	55 (87.3)	54	53 (98.1)	53	51 (96.2)

Fever, infections, antibiotics	64	64 (100)	64	47 (73.4)	54	53 (98.1)	54	49 (90.7)
Vaccination	64	63 (98.4)	64	45 (70.3)	54	50 (92.6)	54	45 (83.3)
Dental care	64	64 (100)	64	44 (68.8)	54	53 (98.1)	54	49 (90.7)
Surgery	64	64 (100)	64	48 (75.0)	54	52 (96.3)	54	44 (81.5)
Planning pregnancy	61	48 (78.7)	53	38 (71.7)	48	30 (62.5)	41	32 (78.0)
Who to call	64	64 (100)	64	55 (85.9)	53	51 (96.2)	53	51 (96.2)
Follow-up	64	64 (100)	64	52 (81.3)	54	53 (98.1)	53	49 (92.5)
Self-injection, asepsis	64	62 (96.9)	62	50 (80.6)	54	53 (98.1)	54	47 (87.0)
Travel	64	63 (98.4)	63	42 (66.7)	54	48 (88.9)	54	42 (77.8)
Storage, cold-chain	64	64 (100)	64	57 (89.1)	54	53 (98.1)	54	51 (94.4)
At the end of the interview, nurse's advice								
Self-injection by the patient themself	63	48 (76.2)			53	44 (83.0)		
Doctor's prescription for the injection by a nurse	62	16 (25.8)			53	7 (13.2)		

[a] Number of available data.

The information booklet was provided by 61/63 nurses (96.8%). At 6 months, 85.5 % and 91.4% patients in the control and intervention groups reported that they had received an information leaflet at baseline and 73.6% and 80.7% considered themselves able to self-inject.
At 6 months, 93.2% and 86.4% of the intervention and control groups were still receiving bDMARDs.
